# Supplementary material for: Loss of CTLH component MAEA impairs DNA repair and replication and leads to developmental delay
Source: EMBO Mol Med. 2025 Dec 19;18(2):492–513. doi: 10.1038/s44321-025-00352-x (PMC12905269; doi:10.1038/s44321-025-00352-x)
Supplement: Supplementary file 2 — Table EV2 [file 44321_2025_352_MOESM2_ESM.docx]

**Table EV2. Clinically-observed features of DIADEM.**
