# Supplementary material for: Loss of CTLH component MAEA impairs DNA repair and replication and leads to developmental delay
Source: EMBO Mol Med. 2025 Dec 19;18(2):492–513. doi: 10.1038/s44321-025-00352-x (PMC12905269; doi:10.1038/s44321-025-00352-x)
Supplement: Supplementary file 8 — Appendix [file 44321_2025_352_MOESM8_ESM.pdf]

# Appendix Table S1. Details of statistical tests and outcomes

| Figure | Test used | Outcomes |
|---|---|---|
| 3A | Ordinary one-way ANOVA | siLuc vs. siMAEA-1 p = 0.000000000000003, vs. siMAEA-2 p = 0.000000000000004, vs. siMAEA-3 p = 0.000000000000003, vs. siMAEA-4 p = 0.000000000000003, vs. siRMND5A-1 p = 0.000000000000003, vs. siRMND5A-2 p = 0.000000000000003, vs. siRMND5A-3 p = 0.000000000000003 |
| 3B | Two-tailed Kruskal-Wallis | WT vs. MAEA KO siLuc: DMSO p = 0.0000000001, Camptothecin p = 0.0035027002, WT vs. MAEA KO siCtIP: DMSO p < 0.9999999999 , Camptothecin p < 0.9999999999 |
| 3D | Two-tailed Kruskal-Wallis | WT vs. MAEA KO: DMSO p = 0.0000000000005, Camptothecin p = 0.1447262356453 |
| 3G | Two-tailed Kruskal-Wallis | WT vs. MAEA KO: DMSO p < 0.000000000000001, Camptothecin p < 0.000000000000001 |
| 4G | Two-tailed Kruskal-Wallis | WT vs. eGFP Only: Untreated p = 0.711507342444096, Camptothecin p < 0.000000000000001 |
| 5A | Two-tailed Kruskal-Wallis | WT vs.: E349K p < 0.000000000000001, M396R p < 0.000000000000001, 3S p < 0.000000000000001, eGFP Only p < 0.000000000000001 |
| 5B | Ordinary one-way ANOVA | WT vs.: E349K p = 0.0128, M396R p = 0.0384, 3S p = 0.0168, eGFP Only p = 0.0128 |
| 5C | Ordinary one-way ANOVA | WT vs.: E349K p = 0.000017, M396R p = 0.053762, 3S p = 0.000012, eGFP Only p = 0.000009 |
| 5D | Two-tailed Kruskal-Wallis | WT vs.: E349K p < 0.000000000000001, M396R p = 0.000000000000004, 3S p < 0.000000000000001, eGFP Only p < 0.000000000000001 |
| 5E | Two-tailed Kruskal-Wallis | WT vs.: E349K p < 0.000000000000001, M396R p = 0.0000000000027, 3S p < 0.000000000000001, eGFP Only p < 0.000000000000001 |
| 5F | Two-tailed Kruskal-Wallis | WT vs.: P1 p < 0.000000000000001, P2 p < 0.000000000000001, P4 p = 0.189974582060879 |
| EV3B | Two-tailed Kruskal-Wallis | WT vs. MAEA KO siLuc: DMSO p = 0.0000001, Camptothecin p = 0.4629661, WT vs. MAEA KO siCtIP: DMSO p = 0.0577132, Camptothecin p = 0.9483452 |
| EV4B | Two-tailed Kruskal-Wallis | WT vs. MAEA KO DMSO p = 4.1848e-064, Camptothecin p = 1.5778e-041 to 5 sf |
| EV4D | Two-tailed Kruskal-Wallis | WT vs. MAEA KO DMSO p = 1.9385e-022, Camptothecin p = 3.2226e-125 to 5 sf |
