# Supplementary figures and images for: Loss of CTLH component MAEA impairs DNA repair and replication and leads to developmental delay (part 1 of 2)
Source: EMBO Mol Med. 2025 Dec 19;18(2):492–513. doi: 10.1038/s44321-025-00352-x (PMC12905269; doi:10.1038/s44321-025-00352-x)

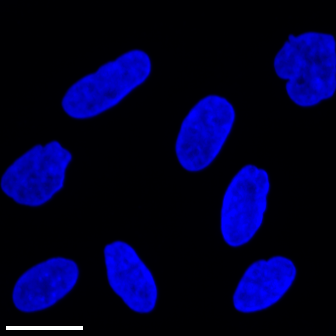

Supplement: Supplementary file 13 — Source data Fig. 3 [file 44321_2025_352_MOESM13_ESM.zip › EMM-2025-21907-V2_SourceDataFig3/3E/WT_DMSO_dapi.png]

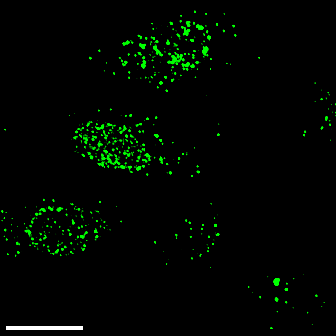

Supplement: Supplementary file 13 — Source data Fig. 3 [file 44321_2025_352_MOESM13_ESM.zip › EMM-2025-21907-V2_SourceDataFig3/3E/KO_CPT_BrdU.png]

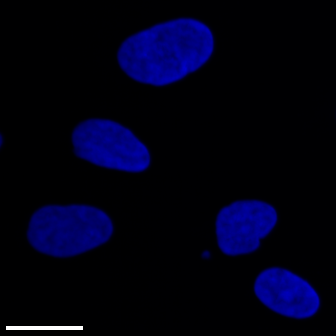

Supplement: Supplementary file 13 — Source data Fig. 3 [file 44321_2025_352_MOESM13_ESM.zip › EMM-2025-21907-V2_SourceDataFig3/3E/KO_CPT_dapi.png]

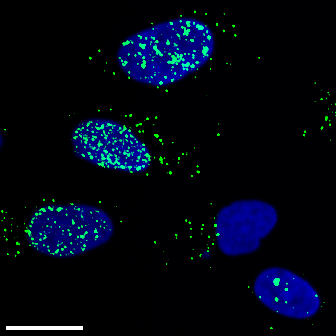

Supplement: Supplementary file 13 — Source data Fig. 3 [file 44321_2025_352_MOESM13_ESM.zip › EMM-2025-21907-V2_SourceDataFig3/3E/KO_CPT_merge.png]

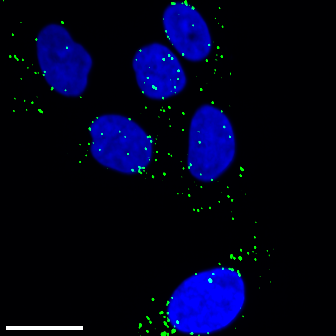

Supplement: Supplementary file 13 — Source data Fig. 3 [file 44321_2025_352_MOESM13_ESM.zip › EMM-2025-21907-V2_SourceDataFig3/3E/KO_DMSO_merge.png]

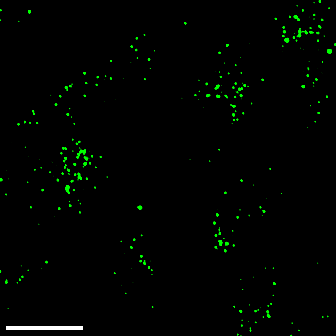

Supplement: Supplementary file 13 — Source data Fig. 3 [file 44321_2025_352_MOESM13_ESM.zip › EMM-2025-21907-V2_SourceDataFig3/3E/WT_DMSO_brdu.png]

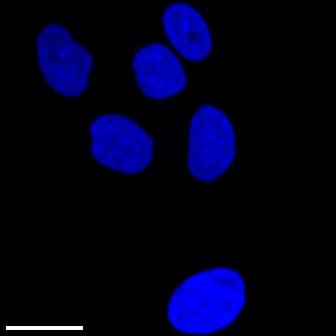

Supplement: Supplementary file 13 — Source data Fig. 3 [file 44321_2025_352_MOESM13_ESM.zip › EMM-2025-21907-V2_SourceDataFig3/3E/KO_DMSO_dapi.png]

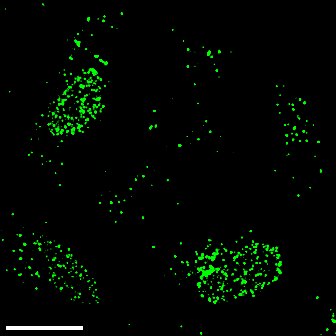

Supplement: Supplementary file 13 — Source data Fig. 3 [file 44321_2025_352_MOESM13_ESM.zip › EMM-2025-21907-V2_SourceDataFig3/3E/WT_CPT_brdu.png]

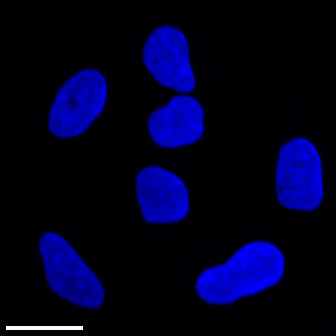

Supplement: Supplementary file 13 — Source data Fig. 3 [file 44321_2025_352_MOESM13_ESM.zip › EMM-2025-21907-V2_SourceDataFig3/3E/WT_CPT_dapi.png]

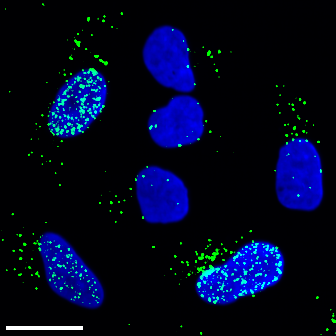

Supplement: Supplementary file 13 — Source data Fig. 3 [file 44321_2025_352_MOESM13_ESM.zip › EMM-2025-21907-V2_SourceDataFig3/3E/WT_CPT_merge.png]

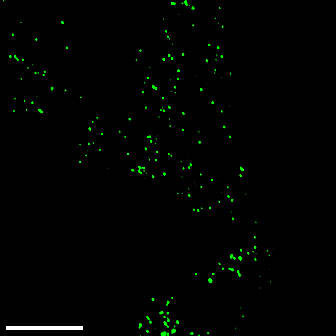

Supplement: Supplementary file 13 — Source data Fig. 3 [file 44321_2025_352_MOESM13_ESM.zip › EMM-2025-21907-V2_SourceDataFig3/3E/KO_DMSO_brdu.png]

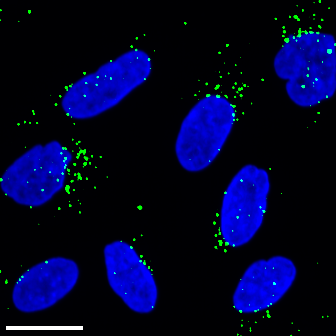

Supplement: Supplementary file 13 — Source data Fig. 3 [file 44321_2025_352_MOESM13_ESM.zip › EMM-2025-21907-V2_SourceDataFig3/3E/WT_DMSO_merge.png]

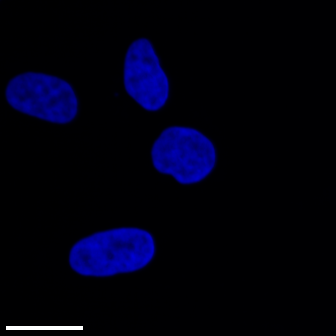

Supplement: Supplementary file 13 — Source data Fig. 3 [file 44321_2025_352_MOESM13_ESM.zip › EMM-2025-21907-V2_SourceDataFig3/3C/WT_DMSO_DAPI.png]

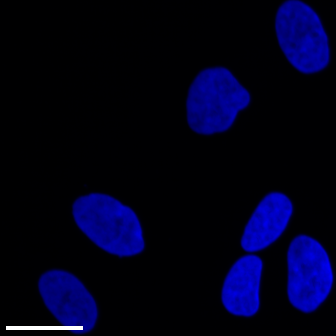

Supplement: Supplementary file 13 — Source data Fig. 3 [file 44321_2025_352_MOESM13_ESM.zip › EMM-2025-21907-V2_SourceDataFig3/3C/KO_CPT_DAPI.png]

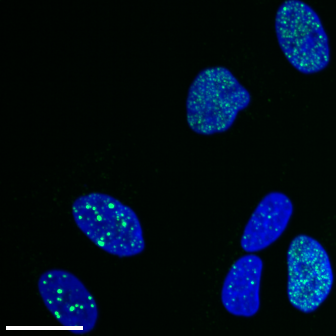

Supplement: Supplementary file 13 — Source data Fig. 3 [file 44321_2025_352_MOESM13_ESM.zip › EMM-2025-21907-V2_SourceDataFig3/3C/KO_CPT_merge.png]

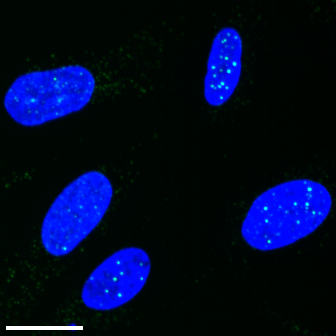

Supplement: Supplementary file 13 — Source data Fig. 3 [file 44321_2025_352_MOESM13_ESM.zip › EMM-2025-21907-V2_SourceDataFig3/3C/KO_DMSO_merge.png]

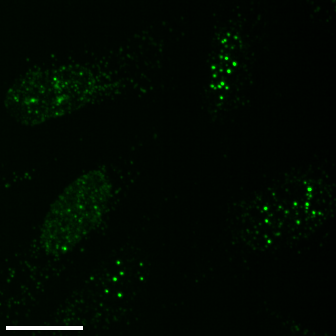

Supplement: Supplementary file 13 — Source data Fig. 3 [file 44321_2025_352_MOESM13_ESM.zip › EMM-2025-21907-V2_SourceDataFig3/3C/KO_DMSO_RPA.png]

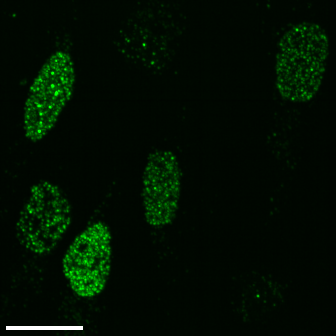

Supplement: Supplementary file 13 — Source data Fig. 3 [file 44321_2025_352_MOESM13_ESM.zip › EMM-2025-21907-V2_SourceDataFig3/3C/WT_CPT_RPA.png]

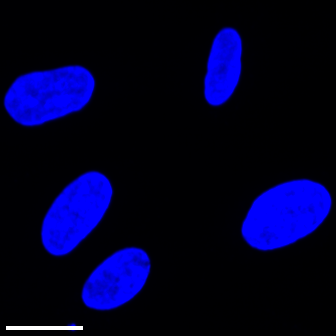

Supplement: Supplementary file 13 — Source data Fig. 3 [file 44321_2025_352_MOESM13_ESM.zip › EMM-2025-21907-V2_SourceDataFig3/3C/KO_DMSO_DAPI.png]

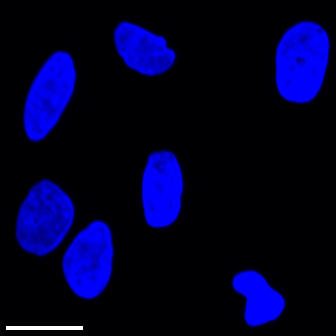

Supplement: Supplementary file 13 — Source data Fig. 3 [file 44321_2025_352_MOESM13_ESM.zip › EMM-2025-21907-V2_SourceDataFig3/3C/WT_CPT_DAPI.png]

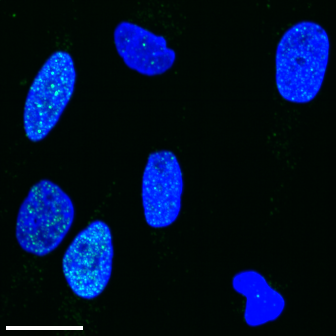

Supplement: Supplementary file 13 — Source data Fig. 3 [file 44321_2025_352_MOESM13_ESM.zip › EMM-2025-21907-V2_SourceDataFig3/3C/WT_CPT_merge.png]

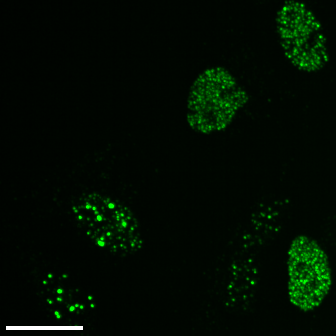

Supplement: Supplementary file 13 — Source data Fig. 3 [file 44321_2025_352_MOESM13_ESM.zip › EMM-2025-21907-V2_SourceDataFig3/3C/KO_CPT_RPA.png]

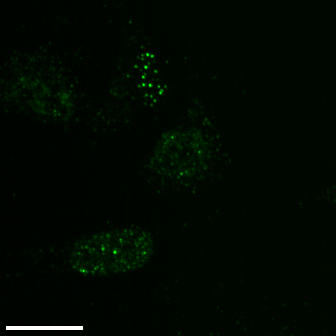

Supplement: Supplementary file 13 — Source data Fig. 3 [file 44321_2025_352_MOESM13_ESM.zip › EMM-2025-21907-V2_SourceDataFig3/3C/WT_DMSO_RPA.png]

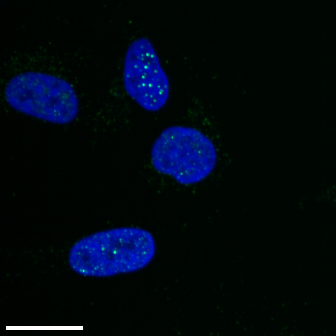

Supplement: Supplementary file 13 — Source data Fig. 3 [file 44321_2025_352_MOESM13_ESM.zip › EMM-2025-21907-V2_SourceDataFig3/3C/WT_DMSO_Merge.png]

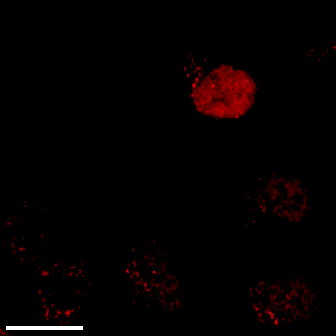

Supplement: Supplementary file 13 — Source data Fig. 3 [file 44321_2025_352_MOESM13_ESM.zip › EMM-2025-21907-V2_SourceDataFig3/3F/WT_CPT_pcna-cenpf.png]

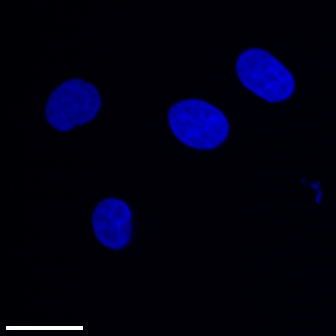

Supplement: Supplementary file 13 — Source data Fig. 3 [file 44321_2025_352_MOESM13_ESM.zip › EMM-2025-21907-V2_SourceDataFig3/3F/WT_DMSO_dapi.png]

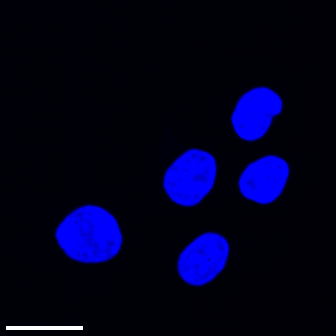

Supplement: Supplementary file 13 — Source data Fig. 3 [file 44321_2025_352_MOESM13_ESM.zip › EMM-2025-21907-V2_SourceDataFig3/3F/KO_CPT_DAPI.png]

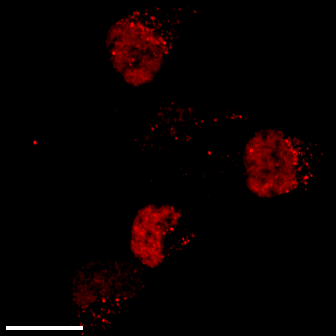

Supplement: Supplementary file 13 — Source data Fig. 3 [file 44321_2025_352_MOESM13_ESM.zip › EMM-2025-21907-V2_SourceDataFig3/3F/KO_DMSO_PCNA-CENPF.png]

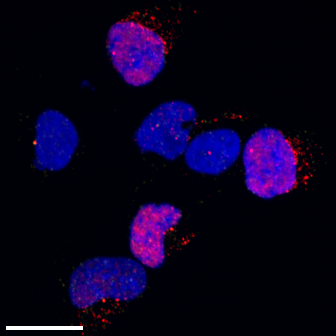

Supplement: Supplementary file 13 — Source data Fig. 3 [file 44321_2025_352_MOESM13_ESM.zip › EMM-2025-21907-V2_SourceDataFig3/3F/KO_DMSO_merge.png]

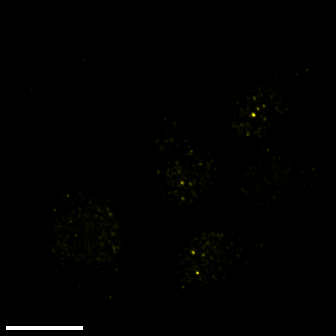

Supplement: Supplementary file 13 — Source data Fig. 3 [file 44321_2025_352_MOESM13_ESM.zip › EMM-2025-21907-V2_SourceDataFig3/3F/KO_CPT_RAD51.png]

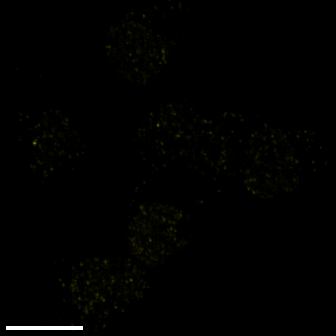

Supplement: Supplementary file 13 — Source data Fig. 3 [file 44321_2025_352_MOESM13_ESM.zip › EMM-2025-21907-V2_SourceDataFig3/3F/KO_DMSO_RAD51.png]

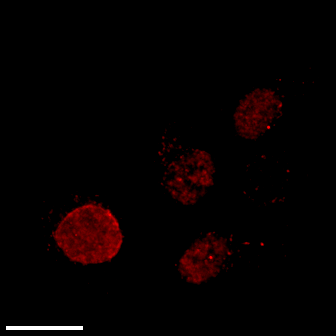

Supplement: Supplementary file 13 — Source data Fig. 3 [file 44321_2025_352_MOESM13_ESM.zip › EMM-2025-21907-V2_SourceDataFig3/3F/KO_CPT_pcna-cenpf.png]

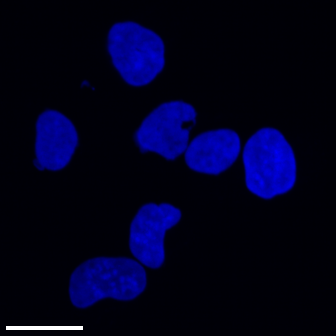

Supplement: Supplementary file 13 — Source data Fig. 3 [file 44321_2025_352_MOESM13_ESM.zip › EMM-2025-21907-V2_SourceDataFig3/3F/KO_DMSO_dapi.png]

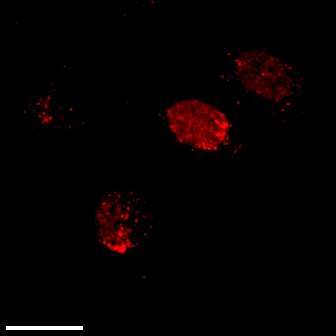

Supplement: Supplementary file 13 — Source data Fig. 3 [file 44321_2025_352_MOESM13_ESM.zip › EMM-2025-21907-V2_SourceDataFig3/3F/WT_DMSO_pcna-cenpf.png]

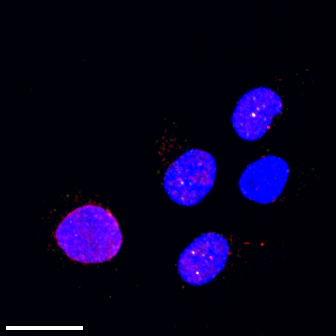

Supplement: Supplementary file 13 — Source data Fig. 3 [file 44321_2025_352_MOESM13_ESM.zip › EMM-2025-21907-V2_SourceDataFig3/3F/KO_CPT_MEGE.png]

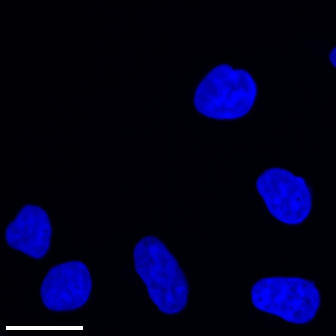

Supplement: Supplementary file 13 — Source data Fig. 3 [file 44321_2025_352_MOESM13_ESM.zip › EMM-2025-21907-V2_SourceDataFig3/3F/WT_CPT_dapi.png]

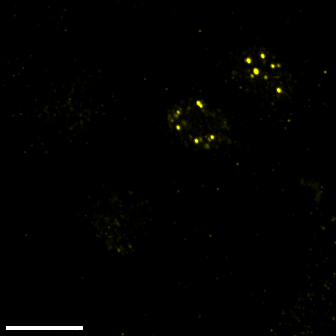

Supplement: Supplementary file 13 — Source data Fig. 3 [file 44321_2025_352_MOESM13_ESM.zip › EMM-2025-21907-V2_SourceDataFig3/3F/WT_DMSO_RAD51.png]

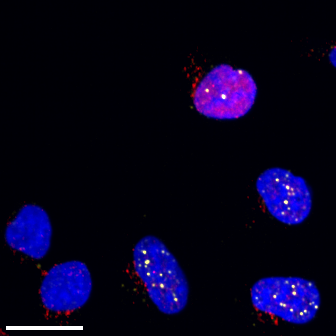

Supplement: Supplementary file 13 — Source data Fig. 3 [file 44321_2025_352_MOESM13_ESM.zip › EMM-2025-21907-V2_SourceDataFig3/3F/WT_CPT_merge.png]

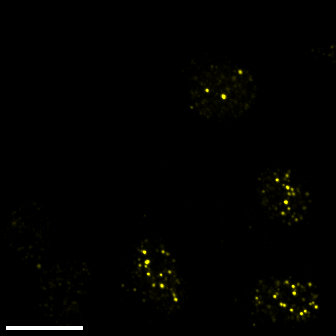

Supplement: Supplementary file 13 — Source data Fig. 3 [file 44321_2025_352_MOESM13_ESM.zip › EMM-2025-21907-V2_SourceDataFig3/3F/WT_CPT_RAD51.png]

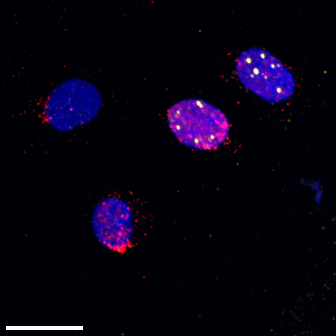

Supplement: Supplementary file 13 — Source data Fig. 3 [file 44321_2025_352_MOESM13_ESM.zip › EMM-2025-21907-V2_SourceDataFig3/3F/WT_DMSO_merge.png]

WT

MAEA KO

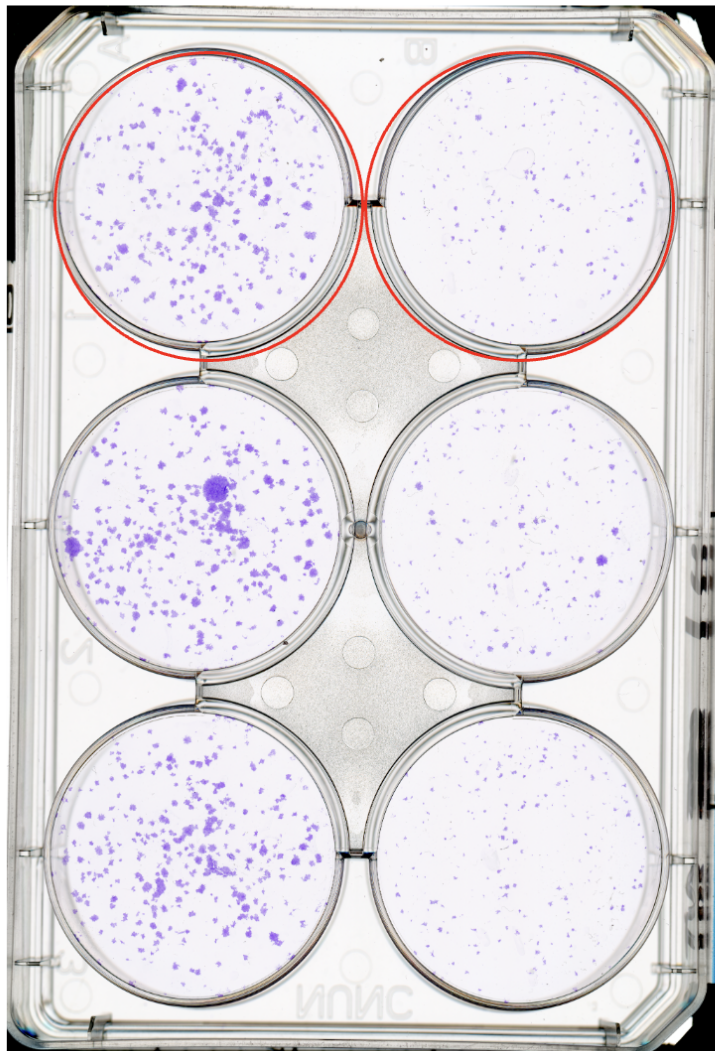

WT

MAEA HM

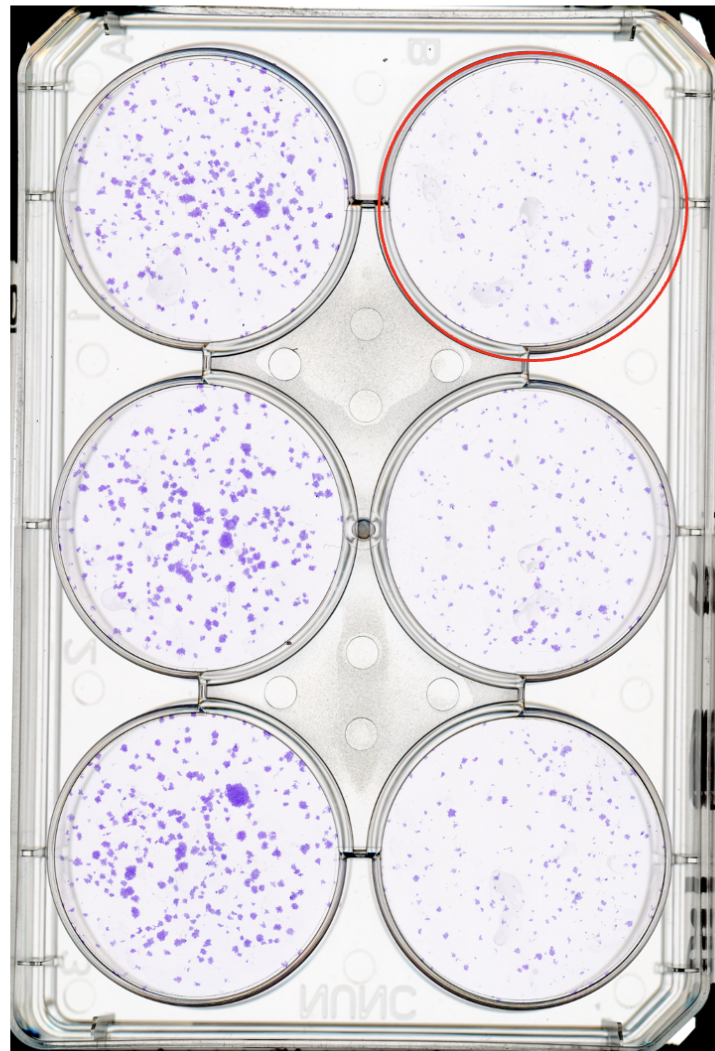

Supplement: Supplementary file 16 — Figure EV1 Source Data [file 44321_2025_352_MOESM16_ESM.zip › EMM-2025-21907-V2_SourceDataFigEV1/EV1E/EV1E_colonies.pdf]

# U2OS

siLuc siMAEA

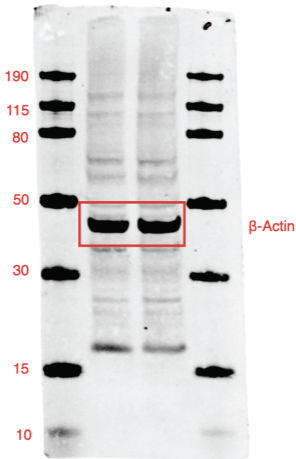

Supplement: Supplementary file 16 — Figure EV1 Source Data [file 44321_2025_352_MOESM16_ESM.zip › EMM-2025-21907-V2_SourceDataFigEV1/EV1G/EV1G_actin.pdf]

# U2OS

siLuc siMAEA

190

115

80

50

30

25

15

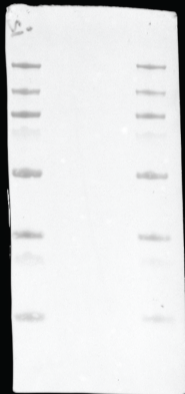

Supplement: Supplementary file 16 — Figure EV1 Source Data [file 44321_2025_352_MOESM16_ESM.zip › EMM-2025-21907-V2_SourceDataFigEV1/EV1G/EV1G_ladders.pdf]

# U2OS

siLuc siMAEA

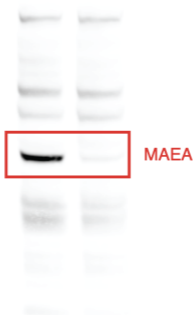

Supplement: Supplementary file 16 — Figure EV1 Source Data [file 44321_2025_352_MOESM16_ESM.zip › EMM-2025-21907-V2_SourceDataFigEV1/EV1G/EV1G_maea.pdf]

WT MAEA KO  
MAEA HM

MAEA

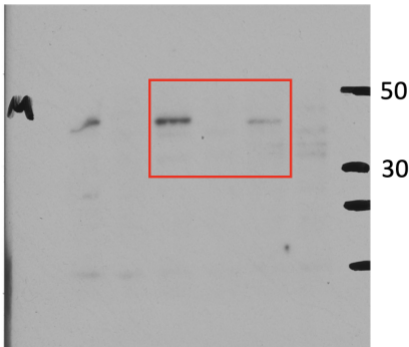

Supplement: Supplementary file 16 — Figure EV1 Source Data [file 44321_2025_352_MOESM16_ESM.zip › EMM-2025-21907-V2_SourceDataFigEV1/EV1A/EV1A_maea.pdf]

WT MAEA KO  
MAEA HM

TOP1

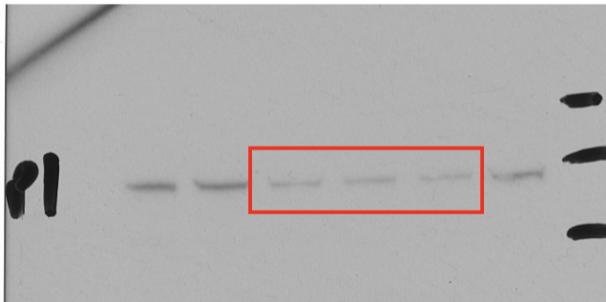

115

Supplement: Supplementary file 16 — Figure EV1 Source Data [file 44321_2025_352_MOESM16_ESM.zip › EMM-2025-21907-V2_SourceDataFigEV1/EV1A/EV1A_TOP1.pdf]

WT

MAEA KO

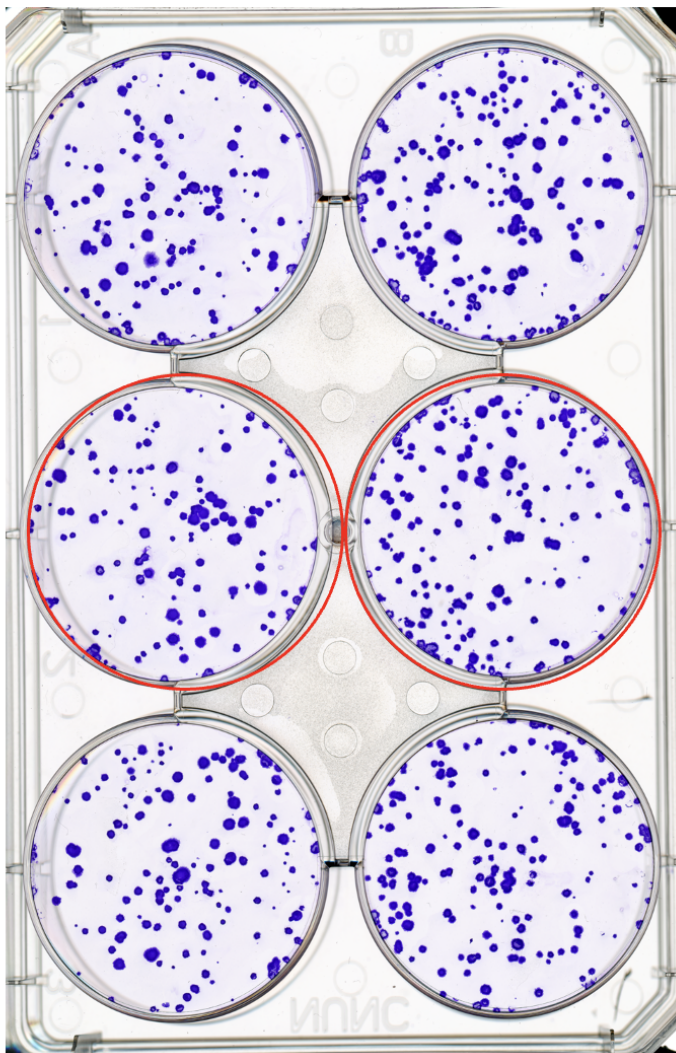

Supplement: Supplementary file 16 — Figure EV1 Source Data [file 44321_2025_352_MOESM16_ESM.zip › EMM-2025-21907-V2_SourceDataFigEV1/EV1F/EV1F_colonies.pdf]

Replicate 1

Replicate 2

Replicate 3

siLuc

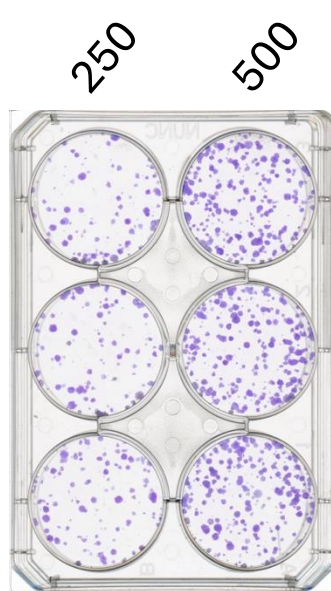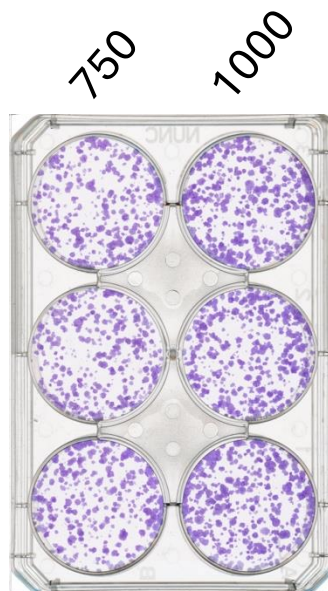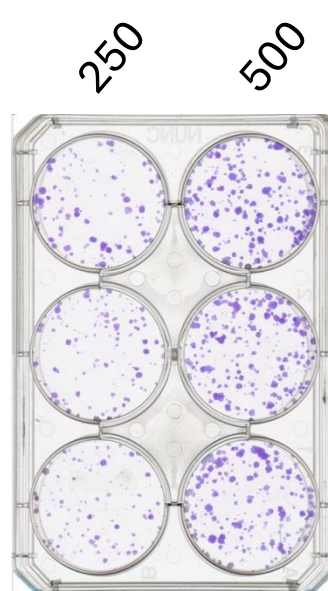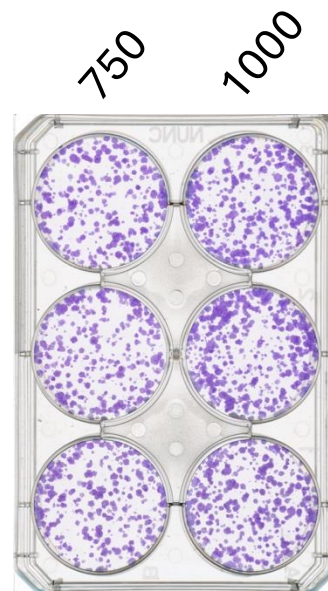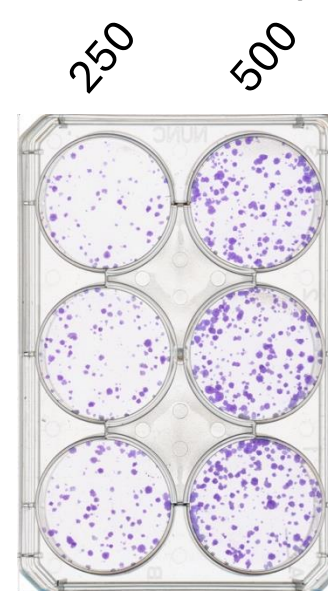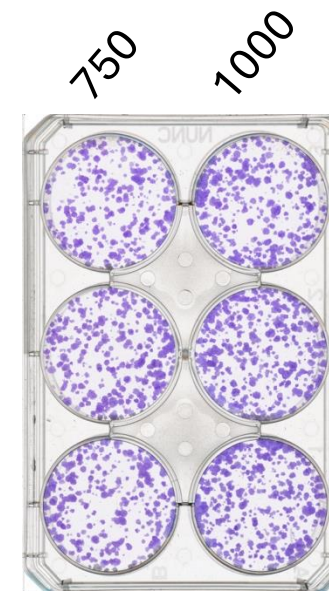

siMAEA

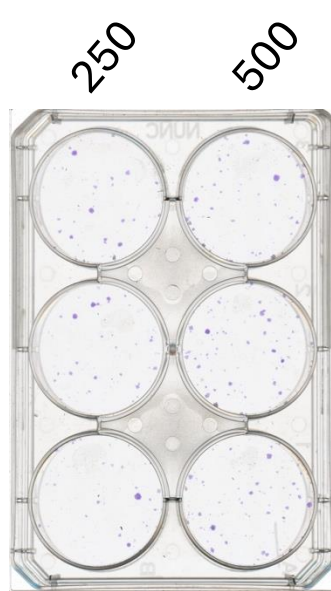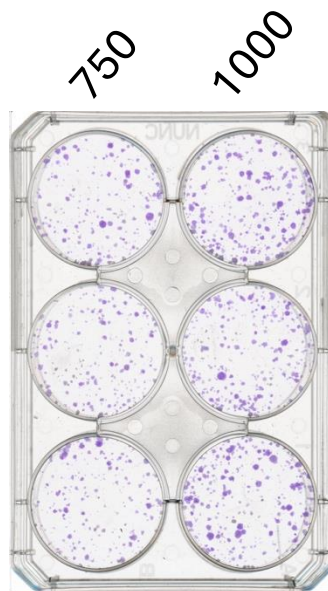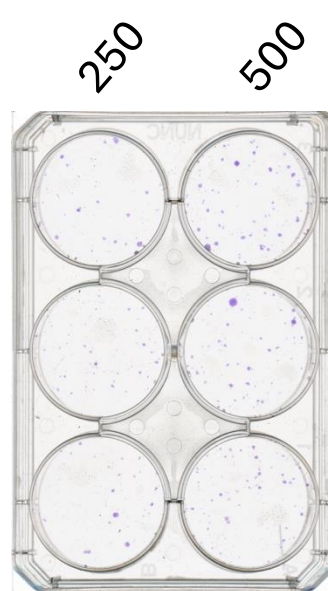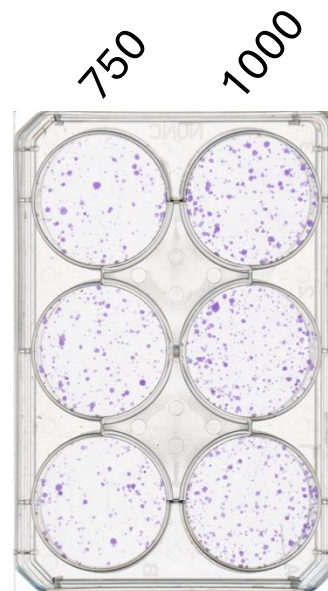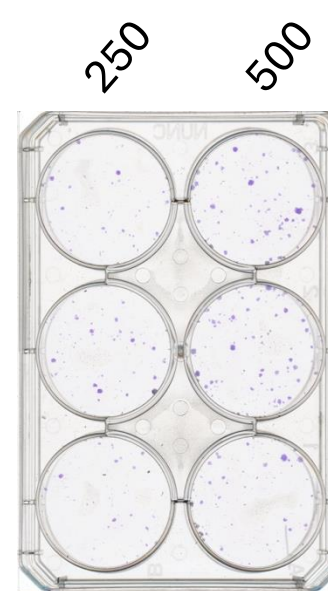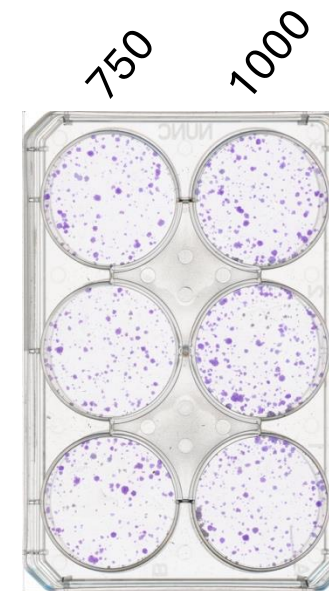

Supplement: Supplementary file 16 — Figure EV1 Source Data [file 44321_2025_352_MOESM16_ESM.zip › EMM-2025-21907-V2_SourceDataFigEV1/EV1H/EV1H.pdf]

MAEA KO + eGFP-

---

WT MAEA KO  
WT 1S 2S 3S E349K M396R eGFP only

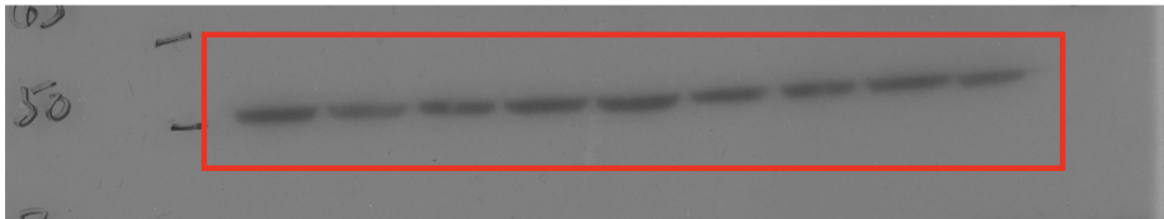

Tubulin

Supplement: Supplementary file 17 — Figure EV2 Source Data [file 44321_2025_352_MOESM17_ESM.zip › EMM-2025-21907-V2_SourceDataFigEV2/EV2B/EV2B_tubulin.pdf]

Original

MAEA KO + eGFP-

eGFP only  
M396R  
E349K  
3S  
2S  
1S  
WT  
MAEA KO  
WT

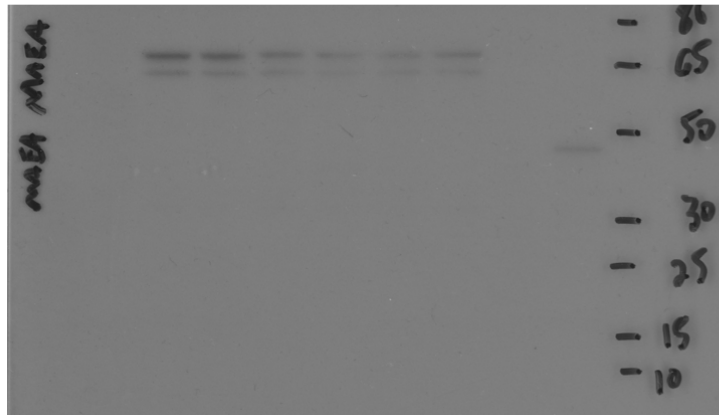

→ flipped for figure

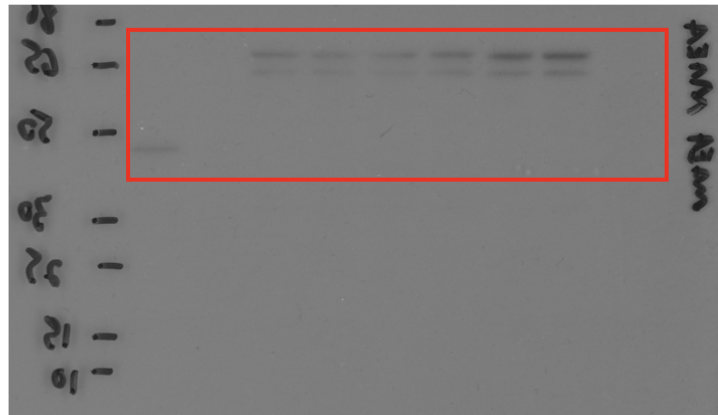

Supplement: Supplementary file 17 — Figure EV2 Source Data [file 44321_2025_352_MOESM17_ESM.zip › EMM-2025-21907-V2_SourceDataFigEV2/EV2B/EV2B_maea.pdf]

MAEA KO + eGFP-

---

WT MAEA KO  
WT 1S 2S 3S E349K M396R eGFP only

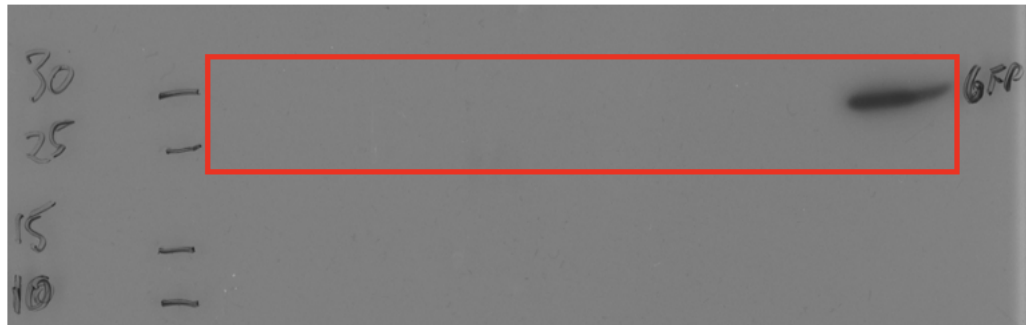

GFP

Supplement: Supplementary file 17 — Figure EV2 Source Data [file 44321_2025_352_MOESM17_ESM.zip › EMM-2025-21907-V2_SourceDataFigEV2/EV2B/EV2B_gfp.pdf]

U2OS  
WT KO

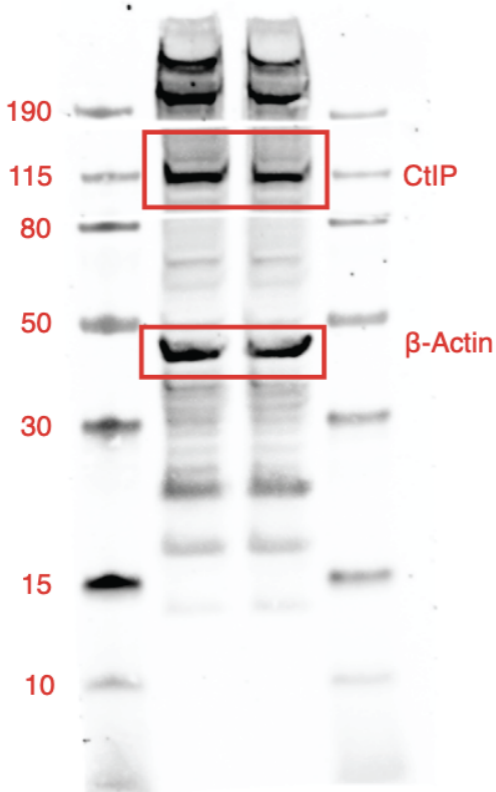

Supplement: Supplementary file 18 — Figure EV3 Source Data [file 44321_2025_352_MOESM18_ESM.zip › EMM-2025-21907-V2_SourceDataFigEV3/EV3F/EV3F_CtIP-Actin-set4.pdf]

U2OS  
WT KO

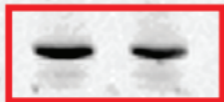

BRCA2

Supplement: Supplementary file 18 — Figure EV3 Source Data [file 44321_2025_352_MOESM18_ESM.zip › EMM-2025-21907-V2_SourceDataFigEV3/EV3F/EV3F_BRCA2-set2.pdf]

# U2OS

## WT KO

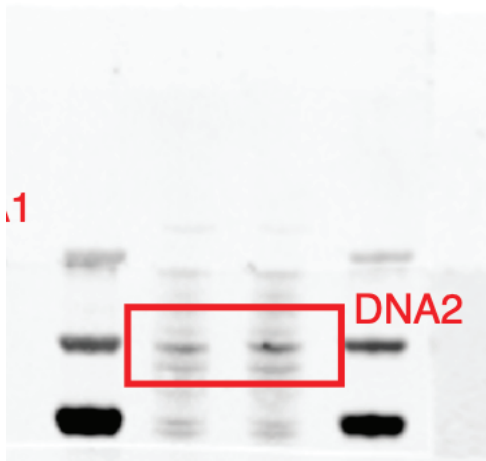

Supplement: Supplementary file 18 — Figure EV3 Source Data [file 44321_2025_352_MOESM18_ESM.zip › EMM-2025-21907-V2_SourceDataFigEV3/EV3F/EV3F_DNA2-set2.pdf]

U2OS  
WT KO

U2OS  
WT KO

U2OS  
WT KO

Set 1

Set 2

Set 3

190

115

80

50

30

15

10

BRCA2

$\beta$ -Actin

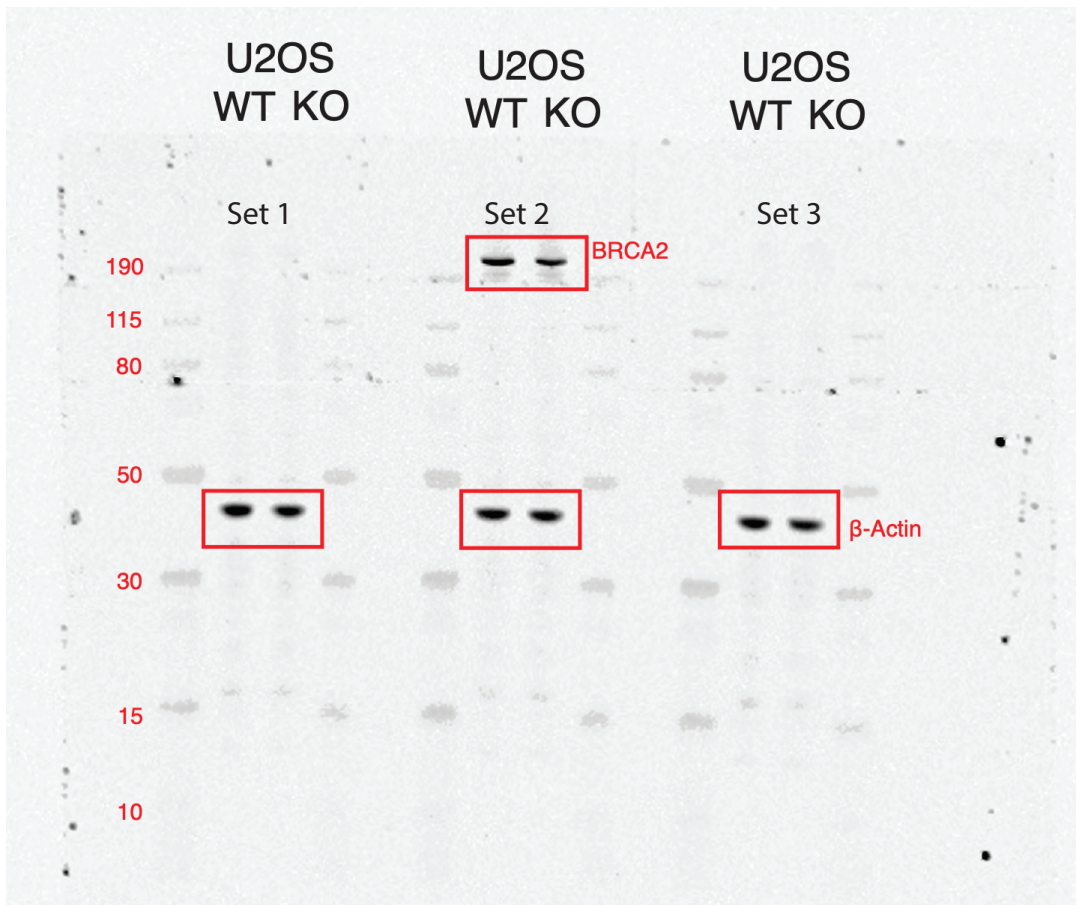

Supplement: Supplementary file 18 — Figure EV3 Source Data [file 44321_2025_352_MOESM18_ESM.zip › EMM-2025-21907-V2_SourceDataFigEV3/EV3F/EV3F_Actin-set1-2-3.pdf]

U2OS  
WT KO

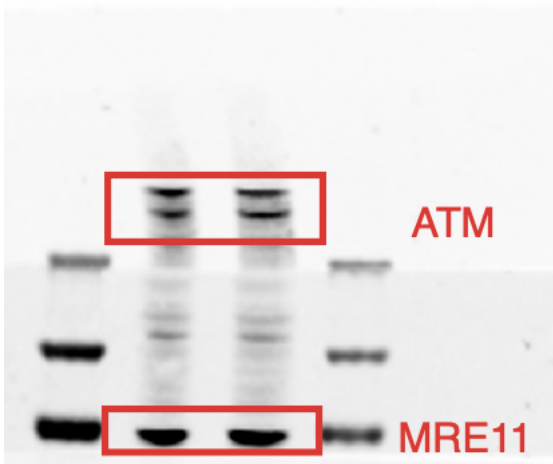

Supplement: Supplementary file 18 — Figure EV3 Source Data [file 44321_2025_352_MOESM18_ESM.zip › EMM-2025-21907-V2_SourceDataFigEV3/EV3F/EV3F_ATMplusMRE11-set3.pdf]

# U2OS WT KO

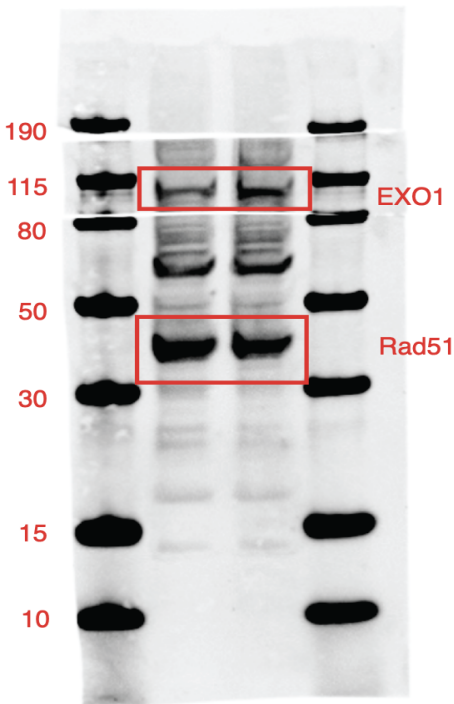

Supplement: Supplementary file 18 — Figure EV3 Source Data [file 44321_2025_352_MOESM18_ESM.zip › EMM-2025-21907-V2_SourceDataFigEV3/EV3F/EV3F_EXO1-RAD51-set4.pdf]

U2OS  
WT KO

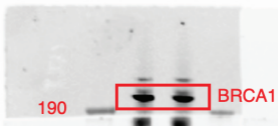

Supplement: Supplementary file 18 — Figure EV3 Source Data [file 44321_2025_352_MOESM18_ESM.zip › EMM-2025-21907-V2_SourceDataFigEV3/EV3F/EV3F_BRCA1-set1.pdf]

Original

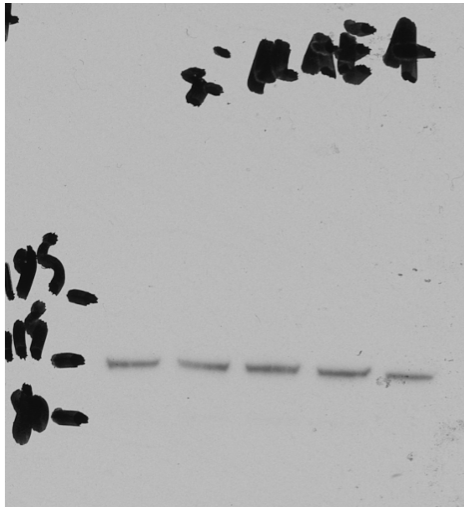

-> rev. orientation for fig:

siLuc  
siMAEA-1  
siMAEA-2  
siMAEA-3  
siMAEA-4

TOP1

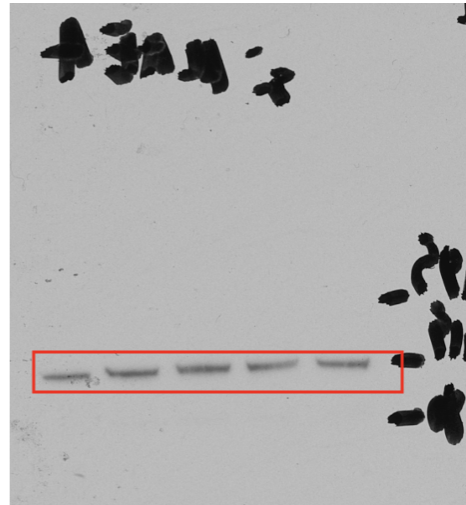

Supplement: Supplementary file 18 — Figure EV3 Source Data [file 44321_2025_352_MOESM18_ESM.zip › EMM-2025-21907-V2_SourceDataFigEV3/EV3A/EV3A_toppanel_TOP1.pdf]

Original

-> rev. orientation for fig:

siLuc

siRMND5A-1

siRMND5A-2

siRMND5A-3

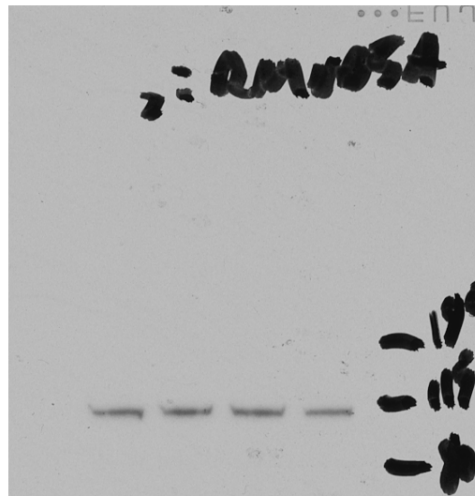

TOP1

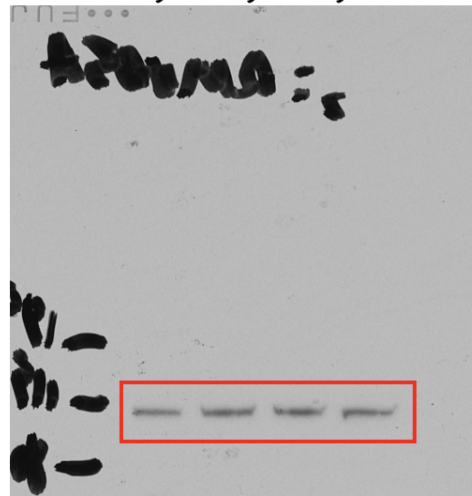

115

Supplement: Supplementary file 18 — Figure EV3 Source Data [file 44321_2025_352_MOESM18_ESM.zip › EMM-2025-21907-V2_SourceDataFigEV3/EV3A/EV3A_bottompanel_TOP1.pdf]

Original

-> rev. orientation for fig:

siLuc

siMAEA-1

siMAEA-2

siMAEA-3

siMAEA-4

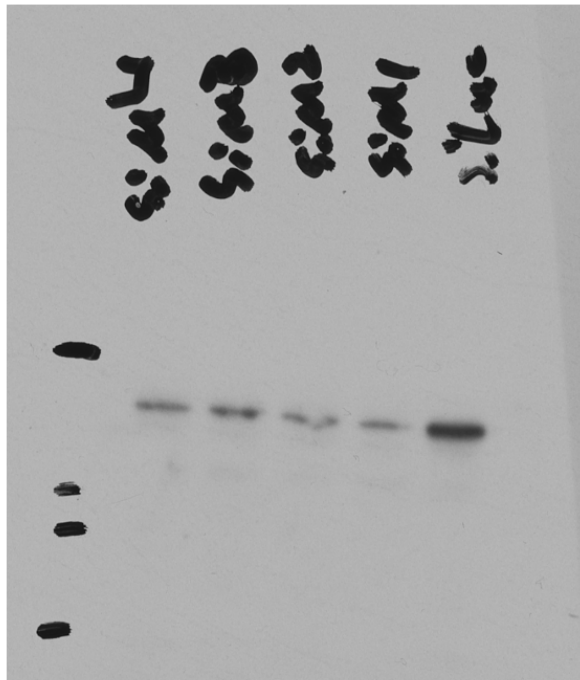

MAEA

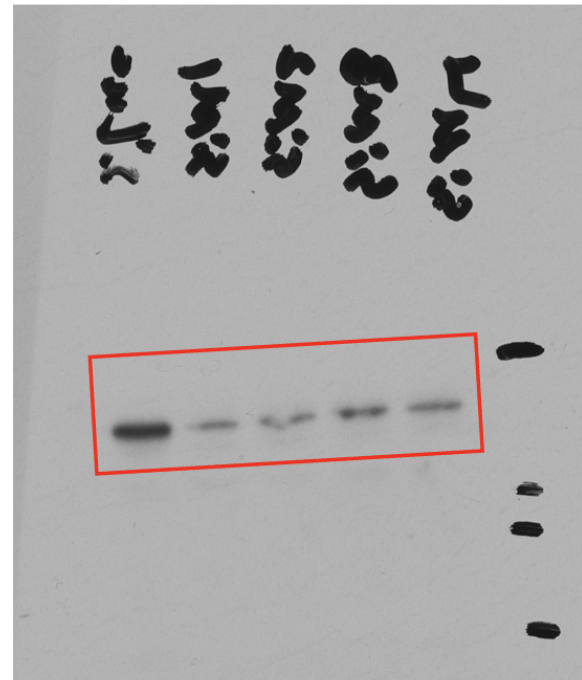

Supplement: Supplementary file 18 — Figure EV3 Source Data [file 44321_2025_352_MOESM18_ESM.zip › EMM-2025-21907-V2_SourceDataFigEV3/EV3A/EV3A_toppanel_MAEA.pdf]

Original

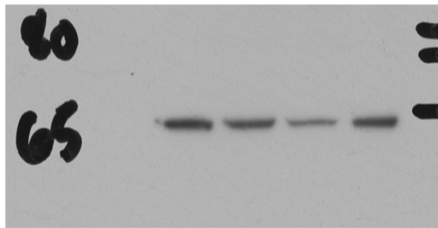

-> rev. orientation for fig:

siLuc  
siRMND5A-1  
siRMND5A-2  
siRMND5A-3

LAMIN B1

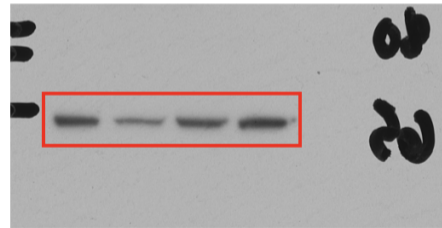

65

Supplement: Supplementary file 18 — Figure EV3 Source Data [file 44321_2025_352_MOESM18_ESM.zip › EMM-2025-21907-V2_SourceDataFigEV3/EV3A/EV3A_bottompanel_LaminB1.pdf]

Original

-> rev. orientation for fig:

siLuc  
siRMND5A-1  
siRMND5A-2  
siRMND5A-3

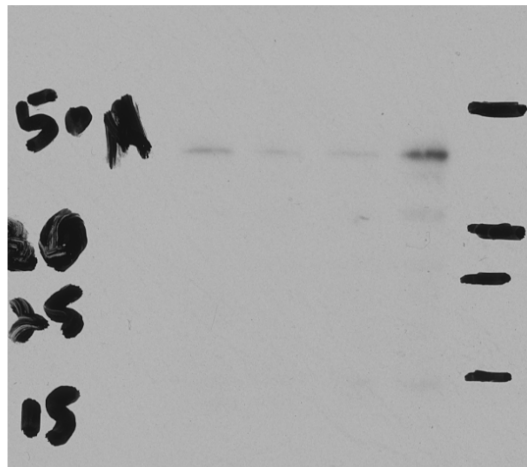

RMND5A

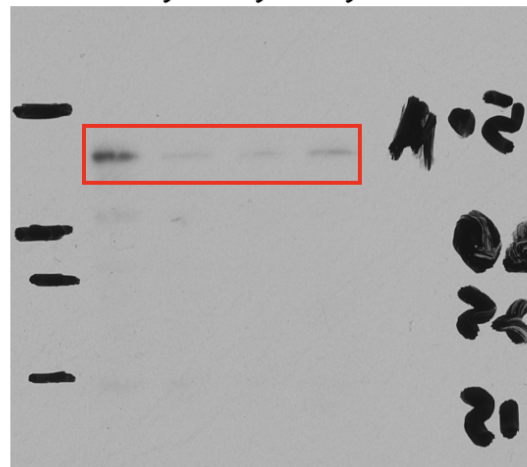

50

Supplement: Supplementary file 18 — Figure EV3 Source Data [file 44321_2025_352_MOESM18_ESM.zip › EMM-2025-21907-V2_SourceDataFigEV3/EV3A/EV3A_bottompanel_RMND5A.pdf]

Original

-> rev. orientation for fig:

siLuc

siMAEA-1

siMAEA-2

siMAEA-3

siMAEA-4

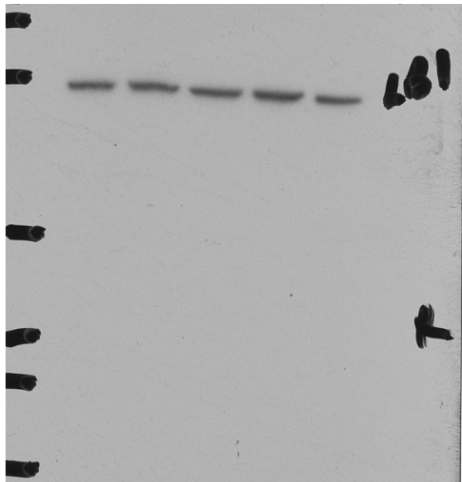

LAMIN B1

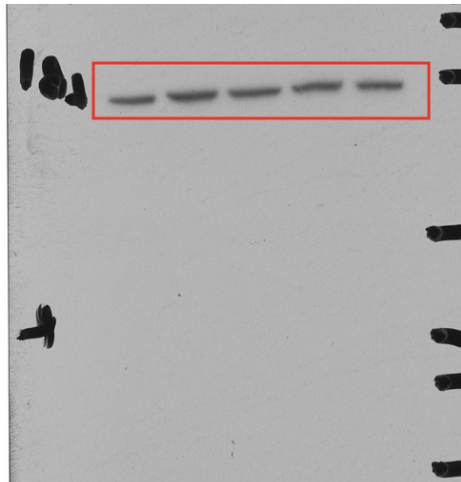

65

Supplement: Supplementary file 18 — Figure EV3 Source Data [file 44321_2025_352_MOESM18_ESM.zip › EMM-2025-21907-V2_SourceDataFigEV3/EV3A/EV3A_toppanel_LaminB1.pdf]

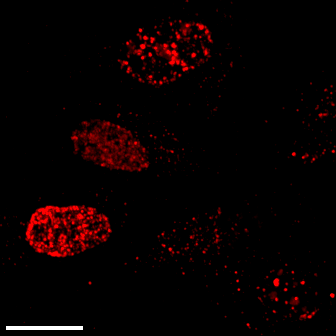

Supplement: Supplementary file 18 — Figure EV3 Source Data [file 44321_2025_352_MOESM18_ESM.zip › EMM-2025-21907-V2_SourceDataFigEV3/EV3D/KO_CPT_gH2AX.png]

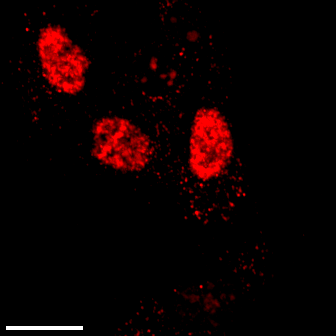

Supplement: Supplementary file 18 — Figure EV3 Source Data [file 44321_2025_352_MOESM18_ESM.zip › EMM-2025-21907-V2_SourceDataFigEV3/EV3D/KO_DMSO_gH2AX.png]

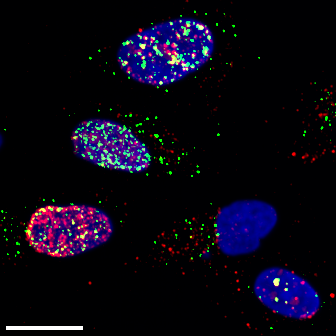

Supplement: Supplementary file 18 — Figure EV3 Source Data [file 44321_2025_352_MOESM18_ESM.zip › EMM-2025-21907-V2_SourceDataFigEV3/EV3D/KO_CPT_merge.png]

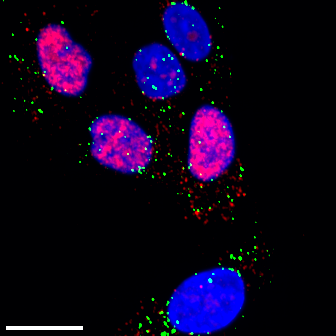

Supplement: Supplementary file 18 — Figure EV3 Source Data [file 44321_2025_352_MOESM18_ESM.zip › EMM-2025-21907-V2_SourceDataFigEV3/EV3D/KO_DMSO_merge.png]

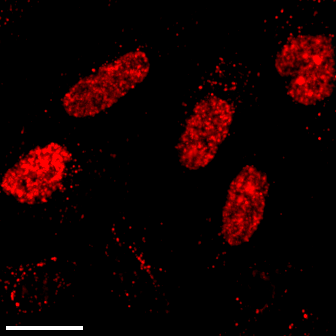

Supplement: Supplementary file 18 — Figure EV3 Source Data [file 44321_2025_352_MOESM18_ESM.zip › EMM-2025-21907-V2_SourceDataFigEV3/EV3D/WT_DMSO_gH2AX.png]

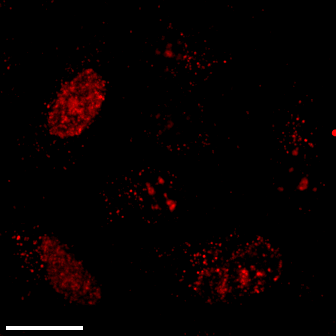

Supplement: Supplementary file 18 — Figure EV3 Source Data [file 44321_2025_352_MOESM18_ESM.zip › EMM-2025-21907-V2_SourceDataFigEV3/EV3D/WT_CPT_gH2AX.png]

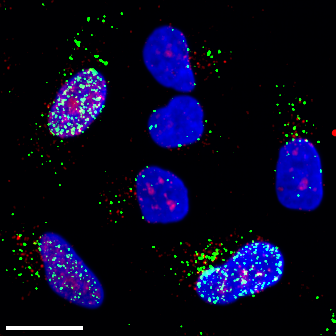

Supplement: Supplementary file 18 — Figure EV3 Source Data [file 44321_2025_352_MOESM18_ESM.zip › EMM-2025-21907-V2_SourceDataFigEV3/EV3D/WT_CPT_merge.png]

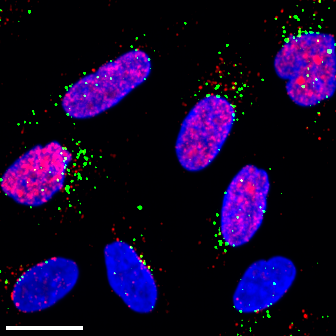

Supplement: Supplementary file 18 — Figure EV3 Source Data [file 44321_2025_352_MOESM18_ESM.zip › EMM-2025-21907-V2_SourceDataFigEV3/EV3D/WT_DMSO_merge.png]

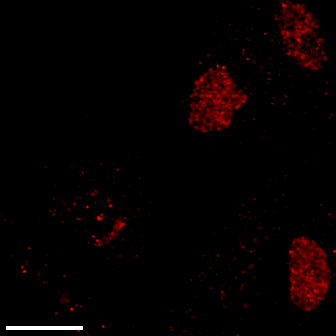

Supplement: Supplementary file 18 — Figure EV3 Source Data [file 44321_2025_352_MOESM18_ESM.zip › EMM-2025-21907-V2_SourceDataFigEV3/EV3C/KO_CPT_gH2AX.png]

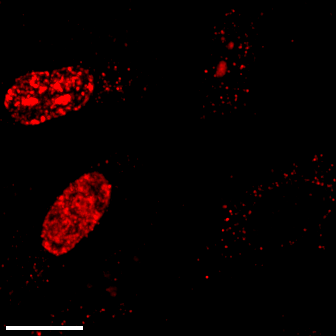

Supplement: Supplementary file 18 — Figure EV3 Source Data [file 44321_2025_352_MOESM18_ESM.zip › EMM-2025-21907-V2_SourceDataFigEV3/EV3C/KO_DMSO_gH2AX.png]

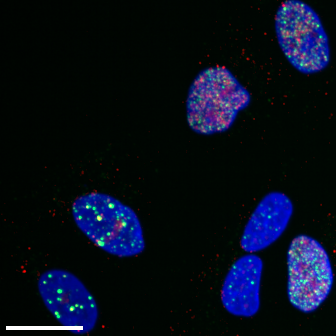

Supplement: Supplementary file 18 — Figure EV3 Source Data [file 44321_2025_352_MOESM18_ESM.zip › EMM-2025-21907-V2_SourceDataFigEV3/EV3C/KO_CPT_merge.png]

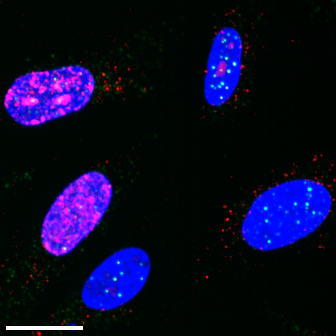

Supplement: Supplementary file 18 — Figure EV3 Source Data [file 44321_2025_352_MOESM18_ESM.zip › EMM-2025-21907-V2_SourceDataFigEV3/EV3C/KO_DMSO_merge.png]

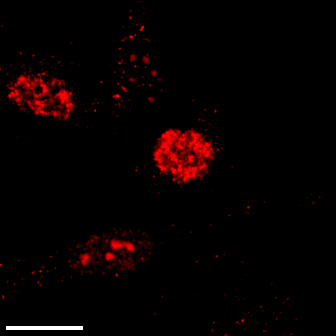

Supplement: Supplementary file 18 — Figure EV3 Source Data [file 44321_2025_352_MOESM18_ESM.zip › EMM-2025-21907-V2_SourceDataFigEV3/EV3C/WT_DMSO_gH2AX.png]

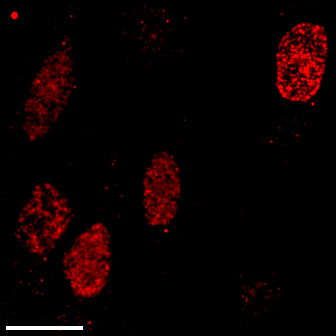

Supplement: Supplementary file 18 — Figure EV3 Source Data [file 44321_2025_352_MOESM18_ESM.zip › EMM-2025-21907-V2_SourceDataFigEV3/EV3C/WT_CPT_gH2AX.png]

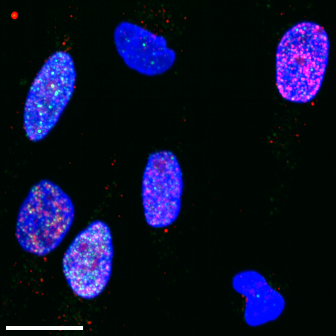

Supplement: Supplementary file 18 — Figure EV3 Source Data [file 44321_2025_352_MOESM18_ESM.zip › EMM-2025-21907-V2_SourceDataFigEV3/EV3C/WT_CPT_merge.png]

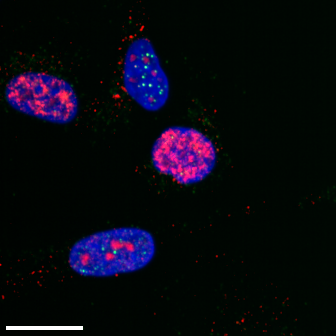

Supplement: Supplementary file 18 — Figure EV3 Source Data [file 44321_2025_352_MOESM18_ESM.zip › EMM-2025-21907-V2_SourceDataFigEV3/EV3C/WT_DMSO_merge.png]

WT

MAEA KO

RAD51

50

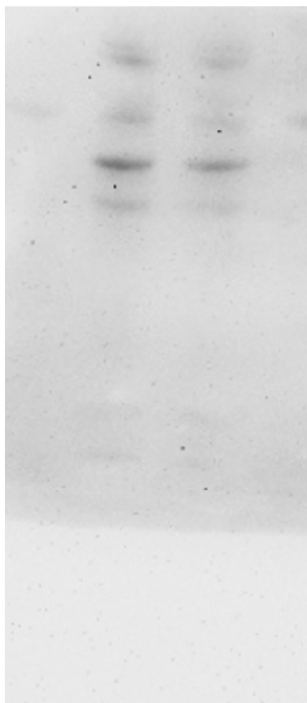

Supplement: Supplementary file 18 — Figure EV3 Source Data [file 44321_2025_352_MOESM18_ESM.zip › EMM-2025-21907-V2_SourceDataFigEV3/EV3E/EV3E_RAD51.pdf]

WT

MAEA KO

Vinculin

115

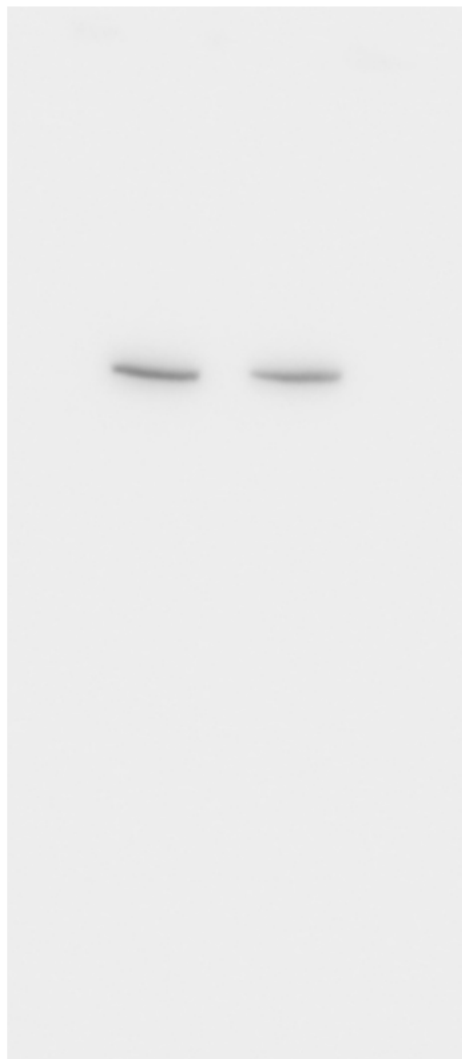

Supplement: Supplementary file 18 — Figure EV3 Source Data [file 44321_2025_352_MOESM18_ESM.zip › EMM-2025-21907-V2_SourceDataFigEV3/EV3E/EV3E_vinculin.pdf]

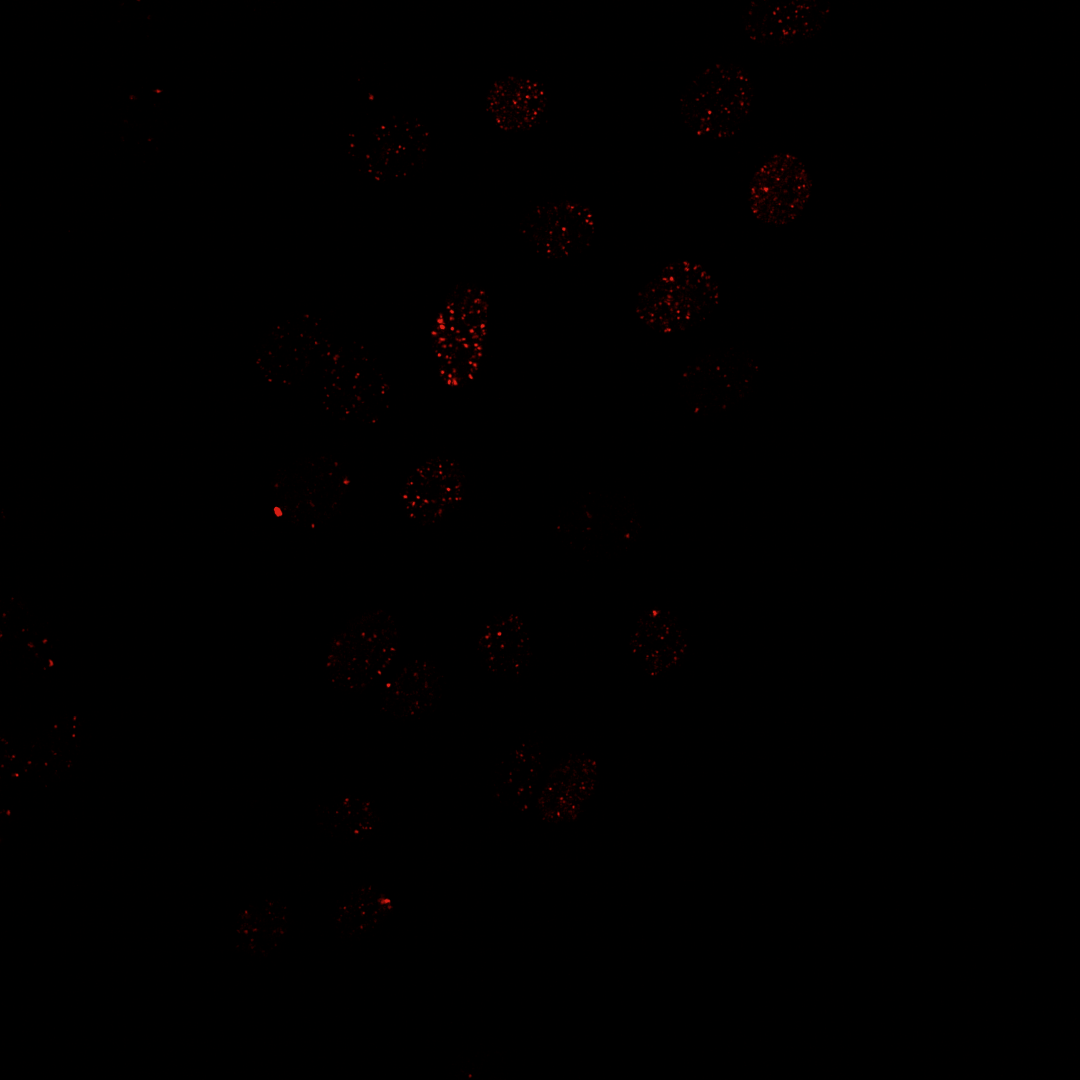

Supplement: Supplementary file 19 — Figure EV4 Source Data [file 44321_2025_352_MOESM19_ESM.zip › EMM-2025-21907-V2_SourceDataFigEV4/EV4F/MAEA E349K gH2AX UT.png]

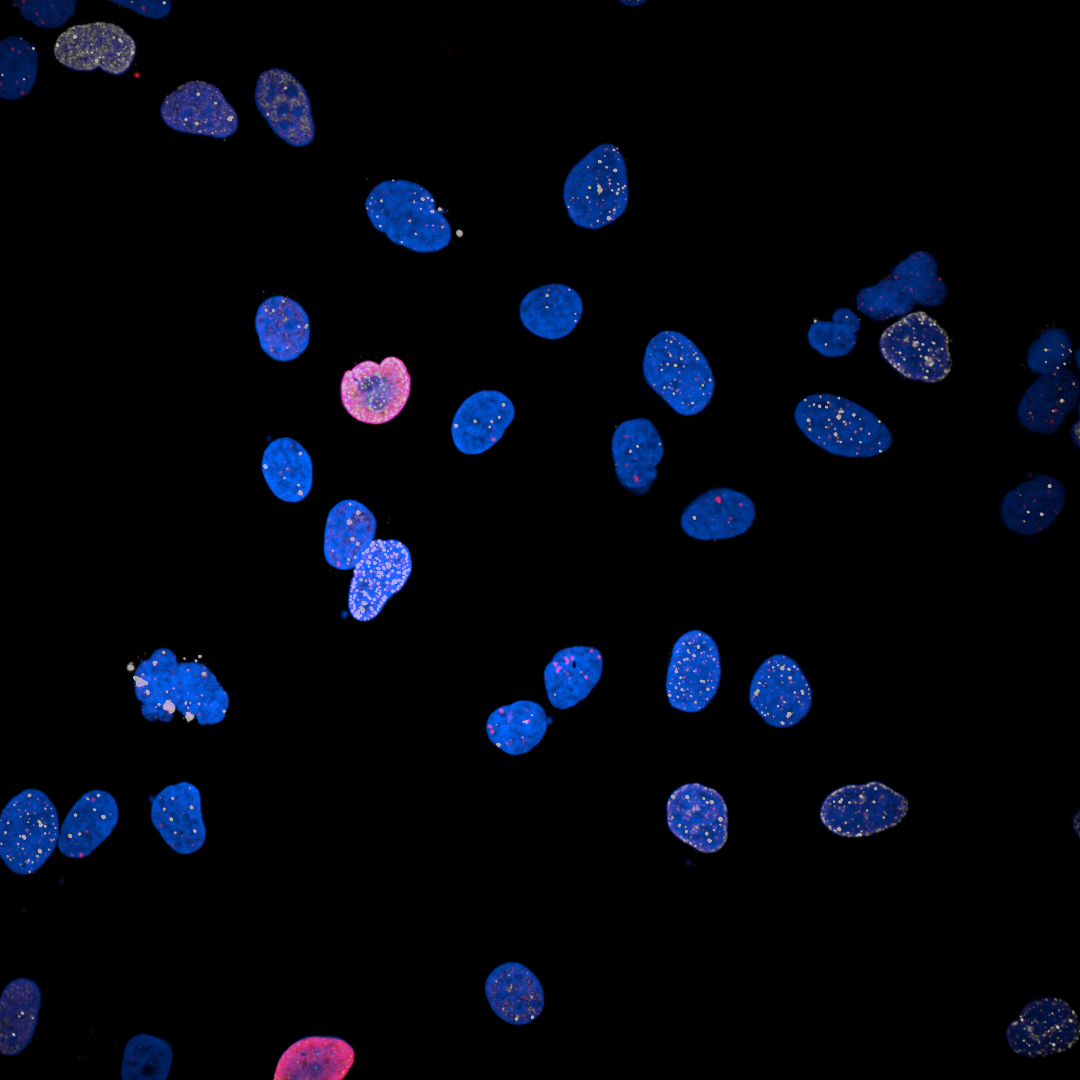

Supplement: Supplementary file 19 — Figure EV4 Source Data [file 44321_2025_352_MOESM19_ESM.zip › EMM-2025-21907-V2_SourceDataFigEV4/EV4F/GFP only merged ATRi.png]

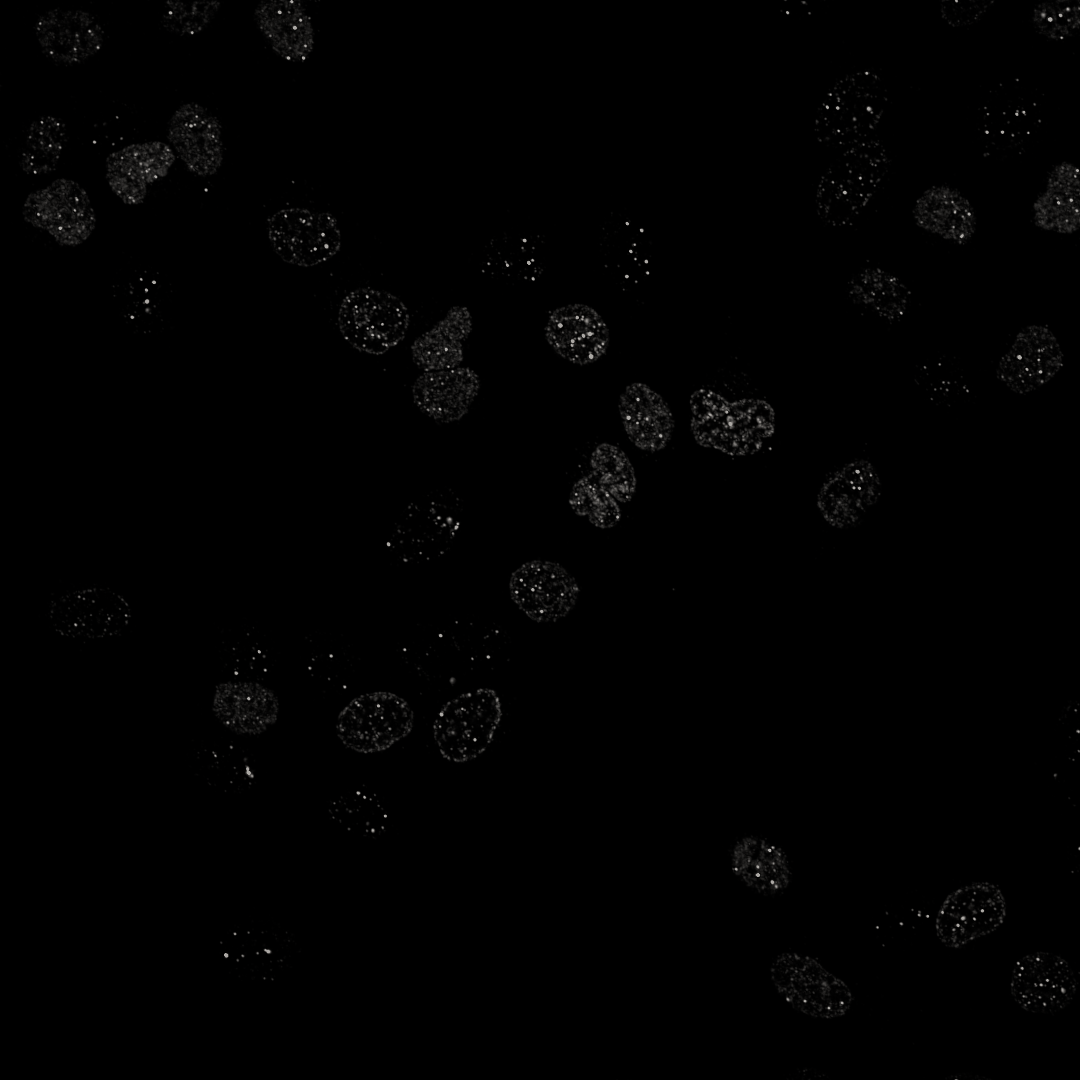

Supplement: Supplementary file 19 — Figure EV4 Source Data [file 44321_2025_352_MOESM19_ESM.zip › EMM-2025-21907-V2_SourceDataFigEV4/EV4F/WT RPA UT.png]

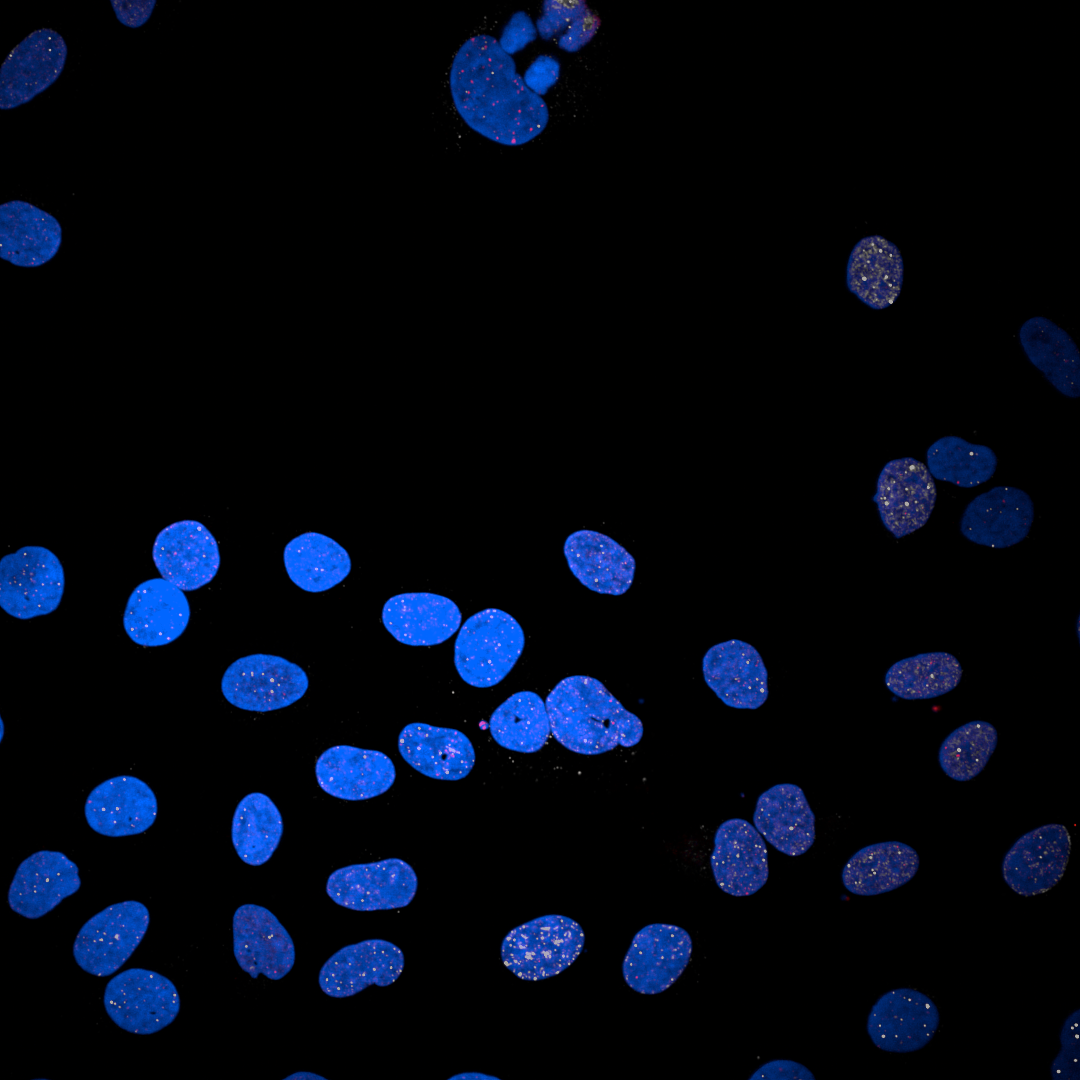

Supplement: Supplementary file 19 — Figure EV4 Source Data [file 44321_2025_352_MOESM19_ESM.zip › EMM-2025-21907-V2_SourceDataFigEV4/EV4F/GFP only merged UT.png]

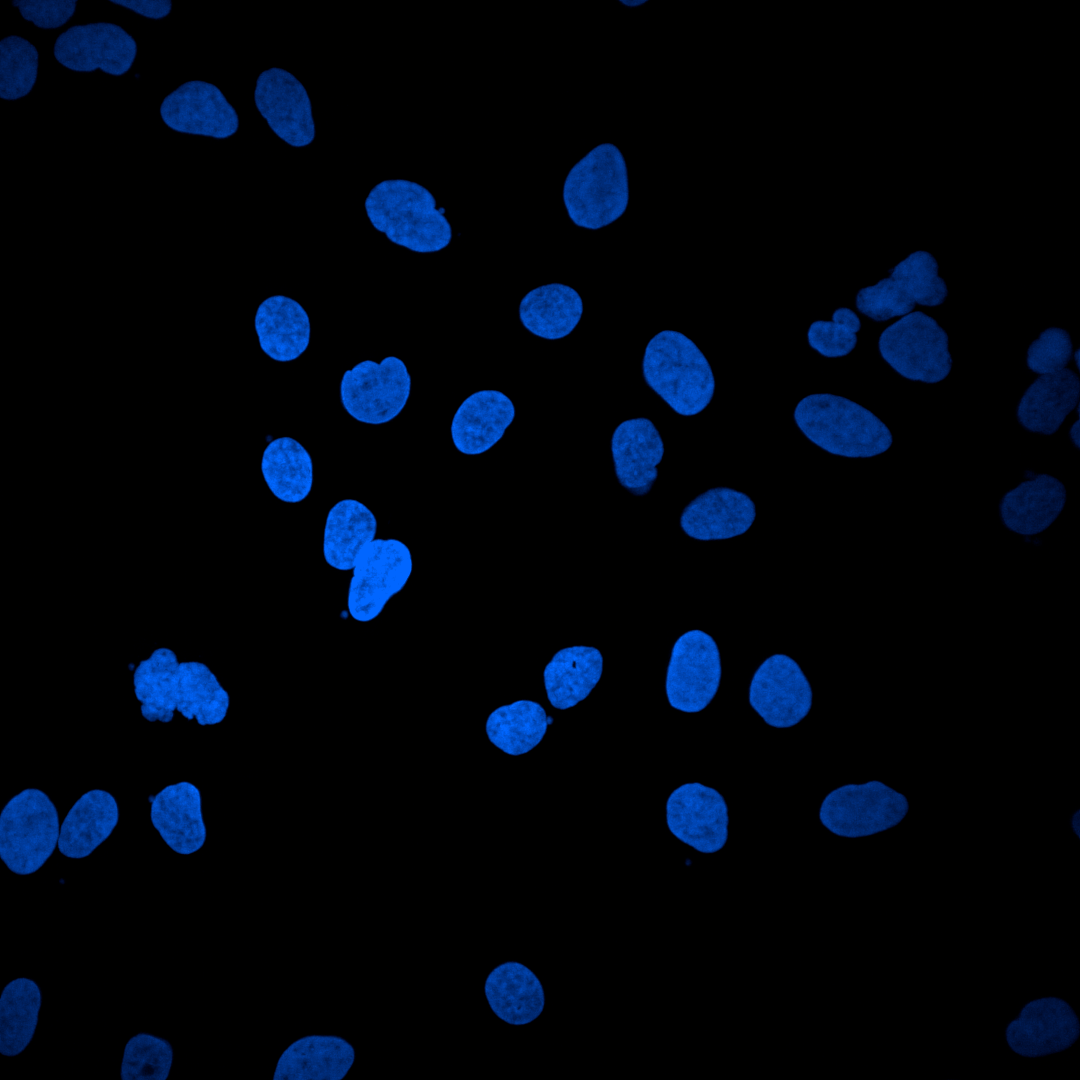

Supplement: Supplementary file 19 — Figure EV4 Source Data [file 44321_2025_352_MOESM19_ESM.zip › EMM-2025-21907-V2_SourceDataFigEV4/EV4F/GFP only DAPI ATRi.png]

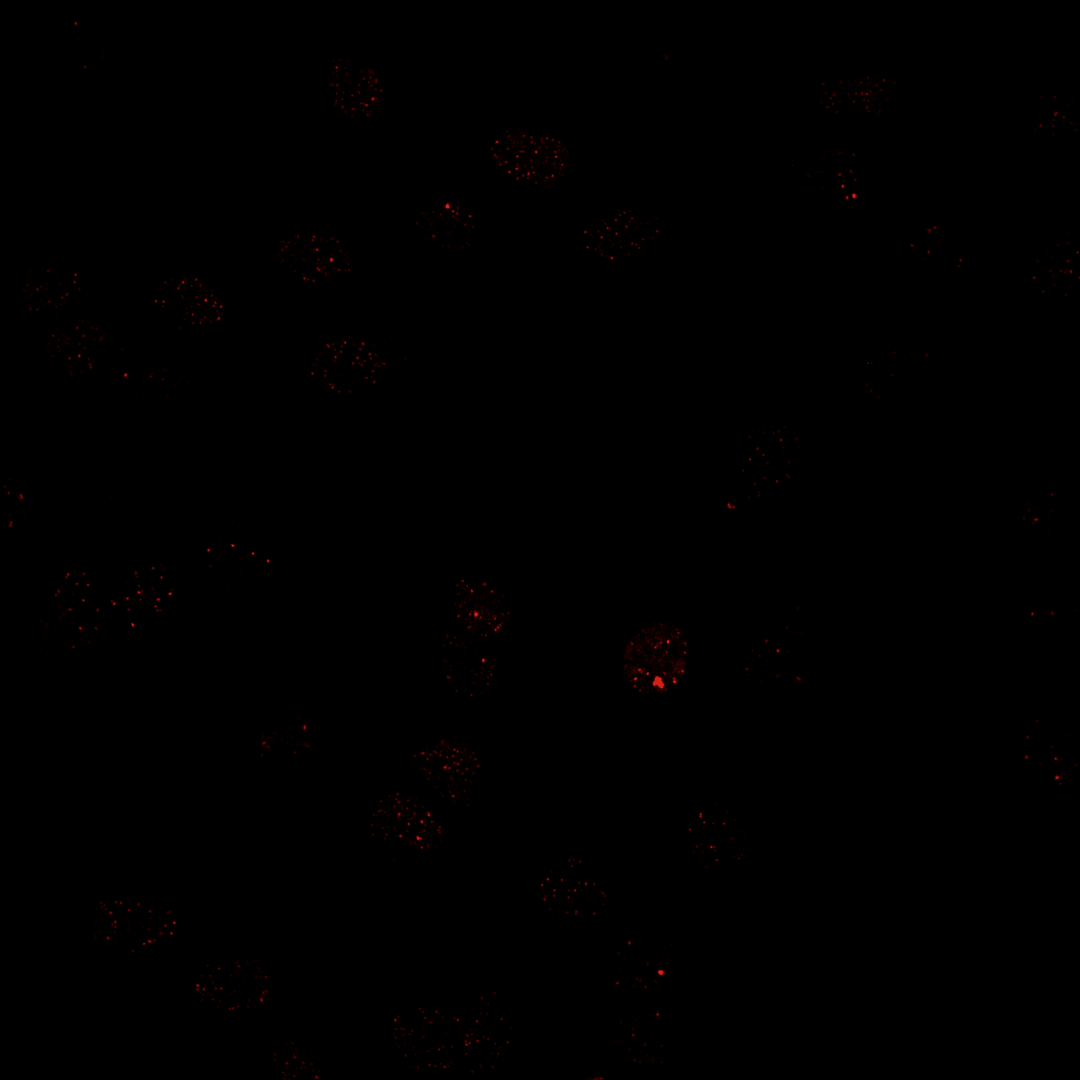

Supplement: Supplementary file 19 — Figure EV4 Source Data [file 44321_2025_352_MOESM19_ESM.zip › EMM-2025-21907-V2_SourceDataFigEV4/EV4F/MAEA M396R gH2AX UT.png]

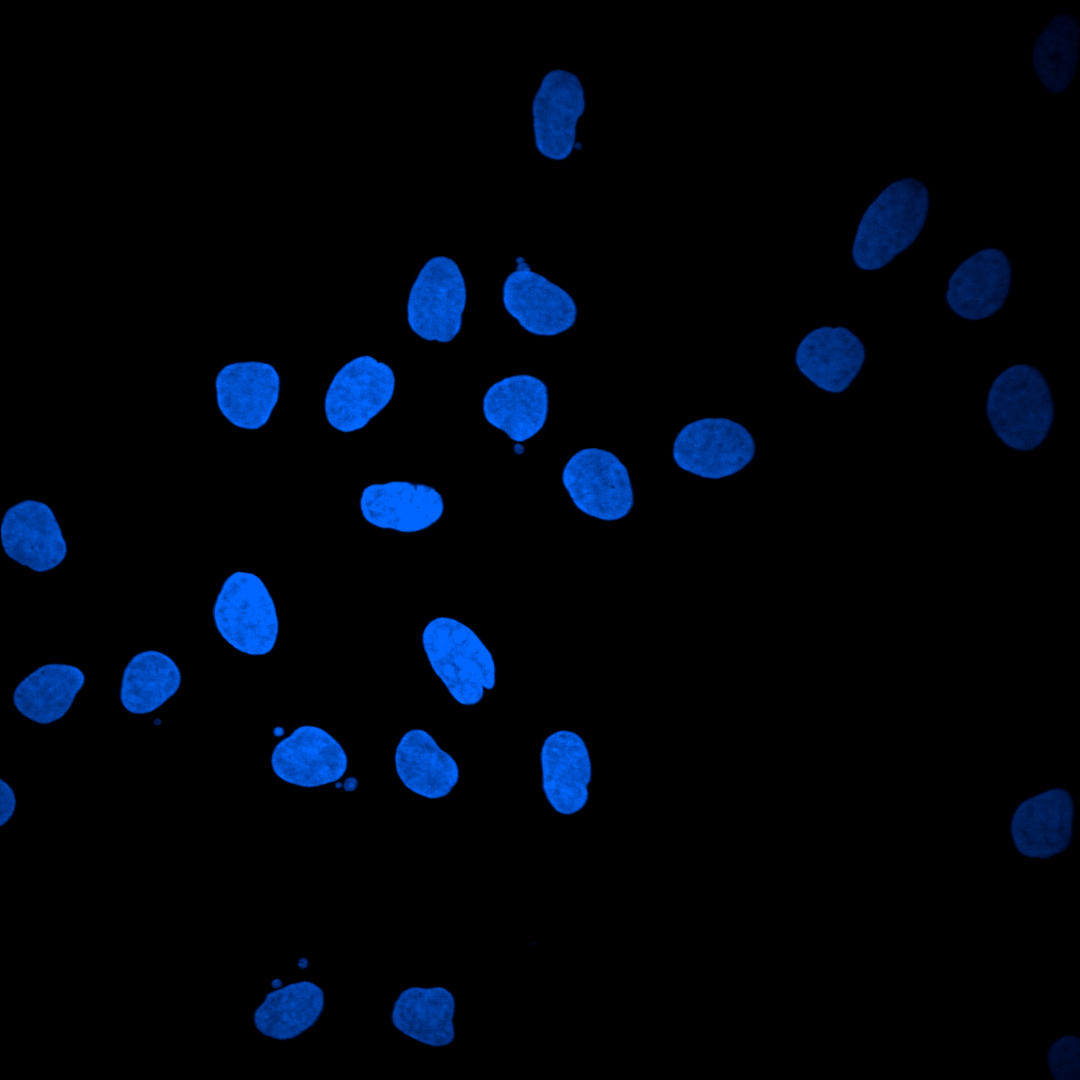

Supplement: Supplementary file 19 — Figure EV4 Source Data [file 44321_2025_352_MOESM19_ESM.zip › EMM-2025-21907-V2_SourceDataFigEV4/EV4F/MAEA M396R DAPI ATRi.png]

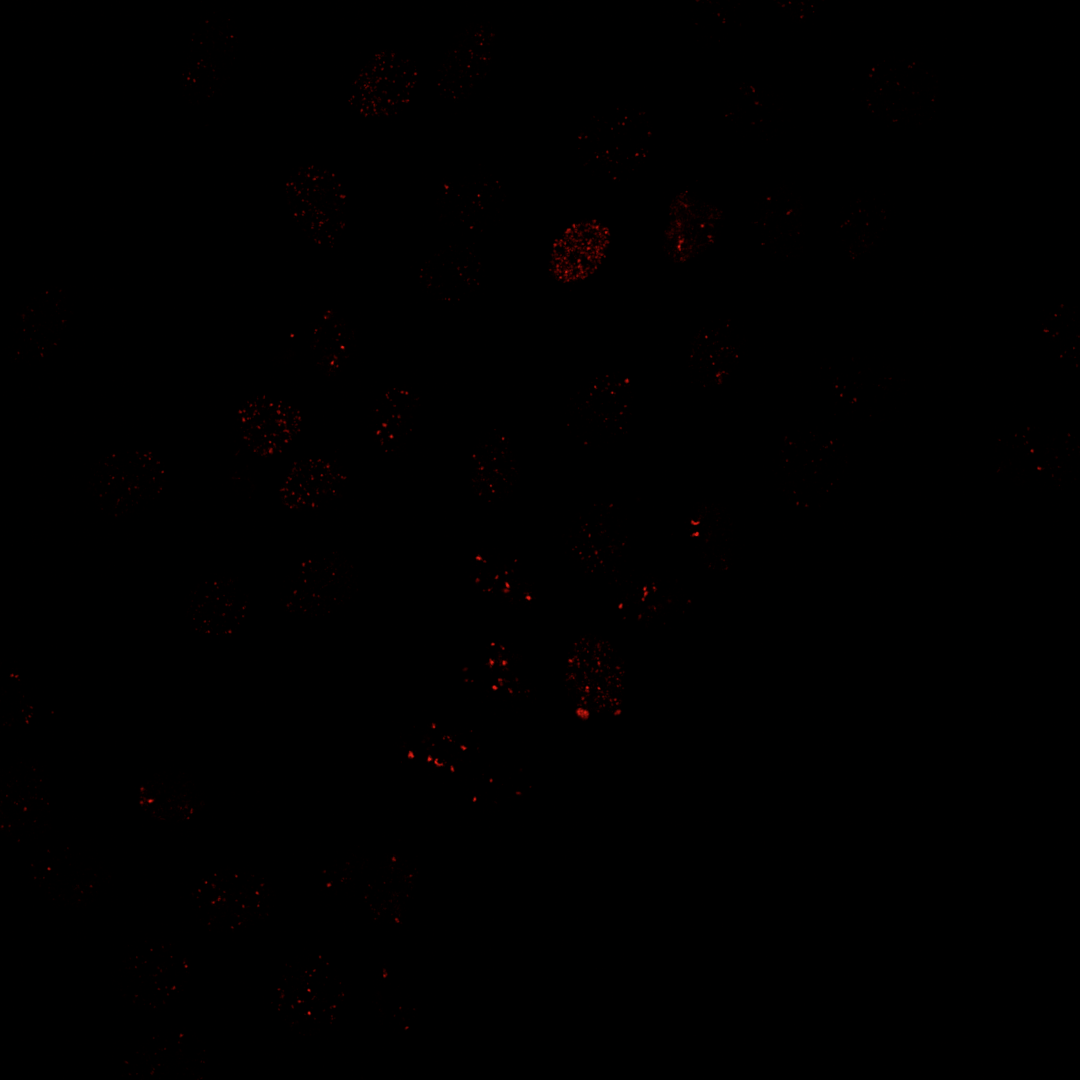

Supplement: Supplementary file 19 — Figure EV4 Source Data [file 44321_2025_352_MOESM19_ESM.zip › EMM-2025-21907-V2_SourceDataFigEV4/EV4F/WT gH2AX ATRi.png]

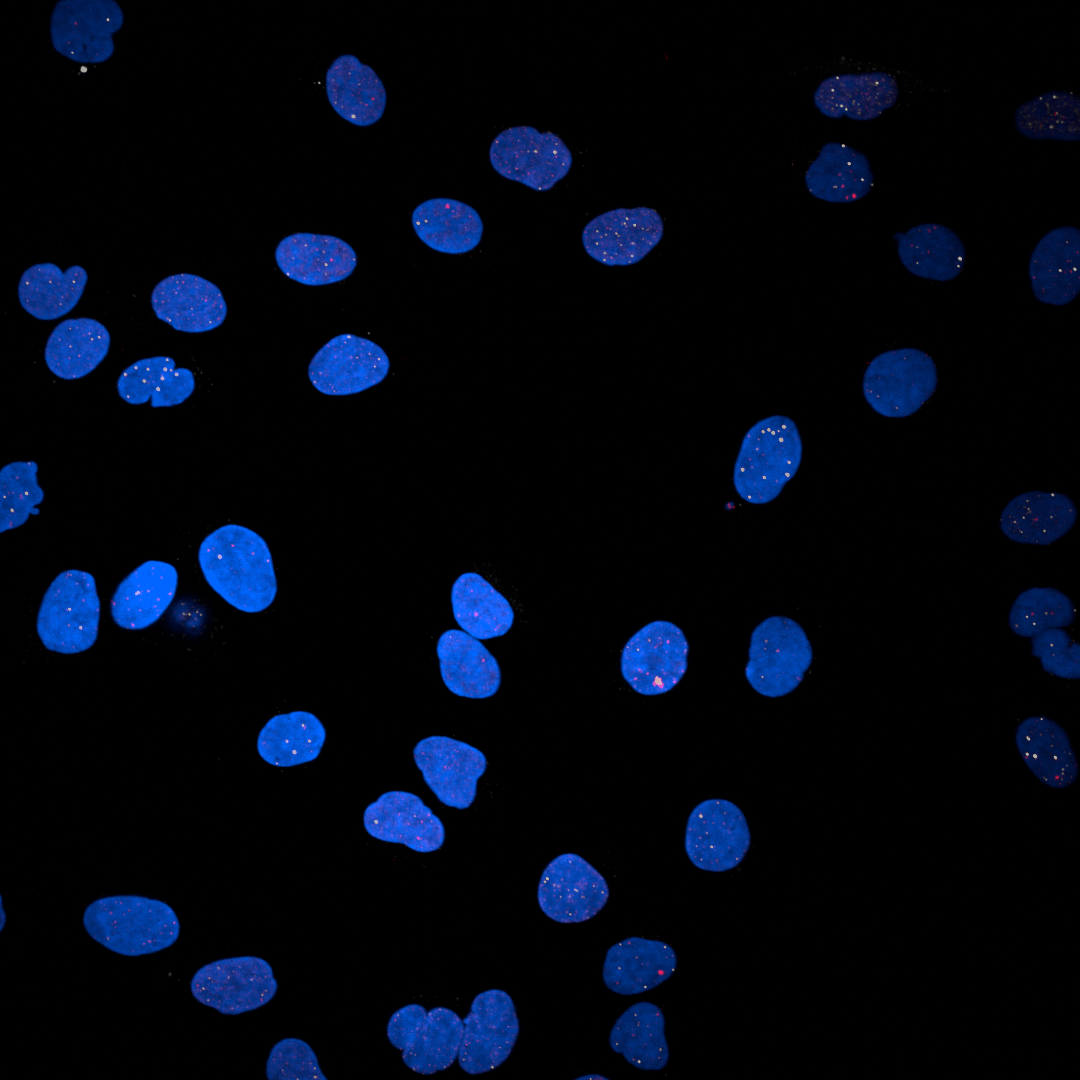

Supplement: Supplementary file 19 — Figure EV4 Source Data [file 44321_2025_352_MOESM19_ESM.zip › EMM-2025-21907-V2_SourceDataFigEV4/EV4F/MAEA M396R merged UT.png]

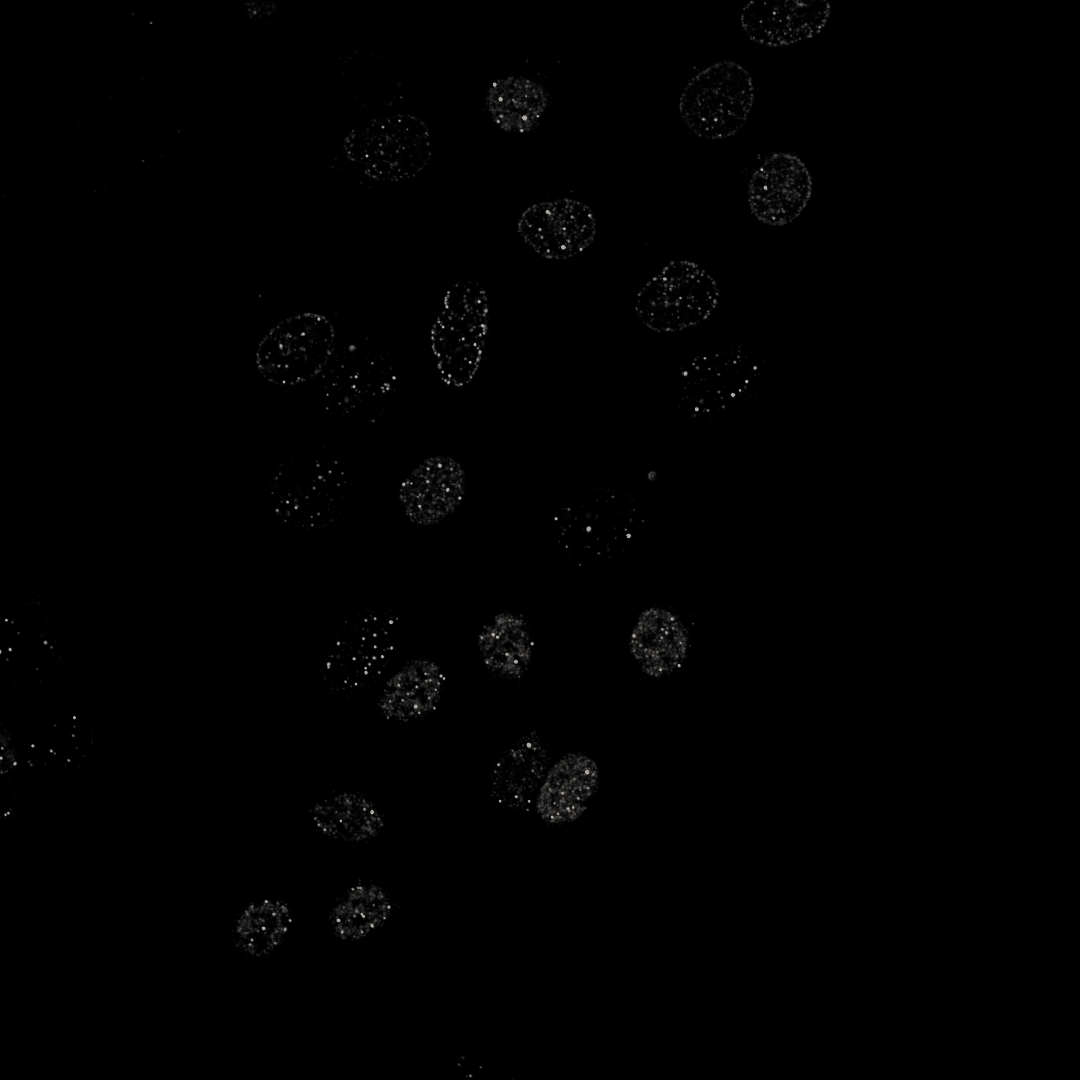

Supplement: Supplementary file 19 — Figure EV4 Source Data [file 44321_2025_352_MOESM19_ESM.zip › EMM-2025-21907-V2_SourceDataFigEV4/EV4F/MAEA E349K RPA UT.png]

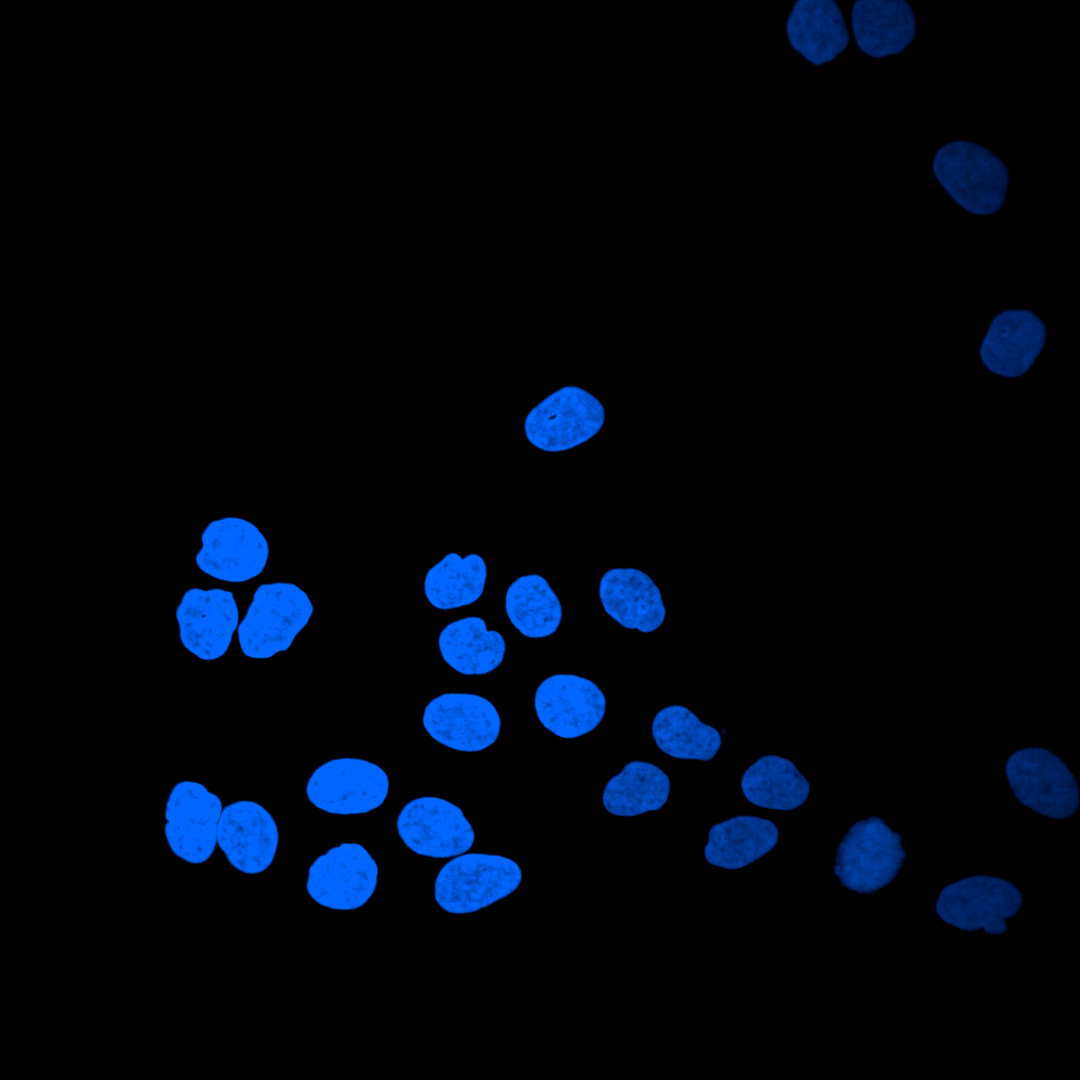

Supplement: Supplementary file 19 — Figure EV4 Source Data [file 44321_2025_352_MOESM19_ESM.zip › EMM-2025-21907-V2_SourceDataFigEV4/EV4F/MAEA 3S DAPI UT.png]

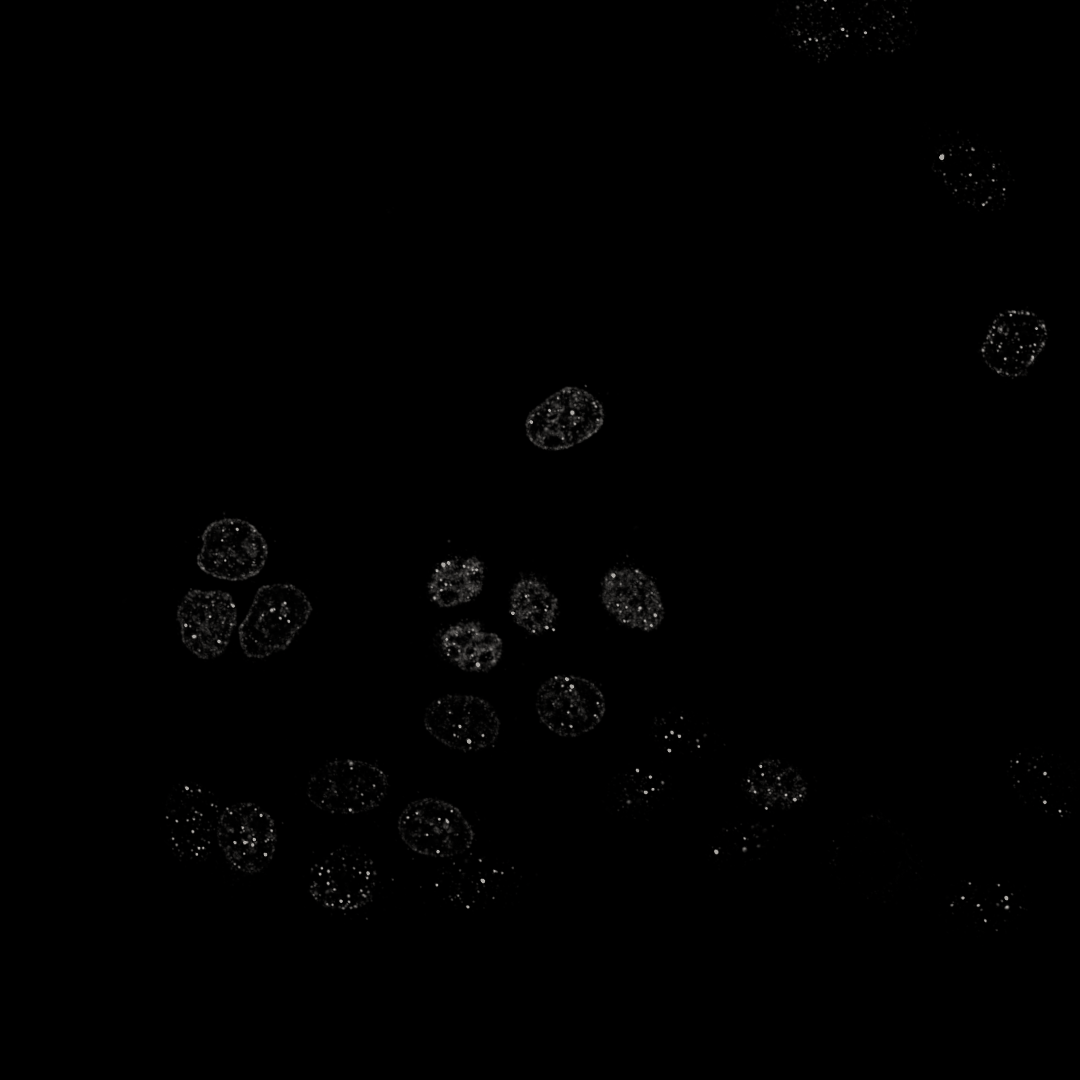

Supplement: Supplementary file 19 — Figure EV4 Source Data [file 44321_2025_352_MOESM19_ESM.zip › EMM-2025-21907-V2_SourceDataFigEV4/EV4F/MAEA 3S RPA UT.png]

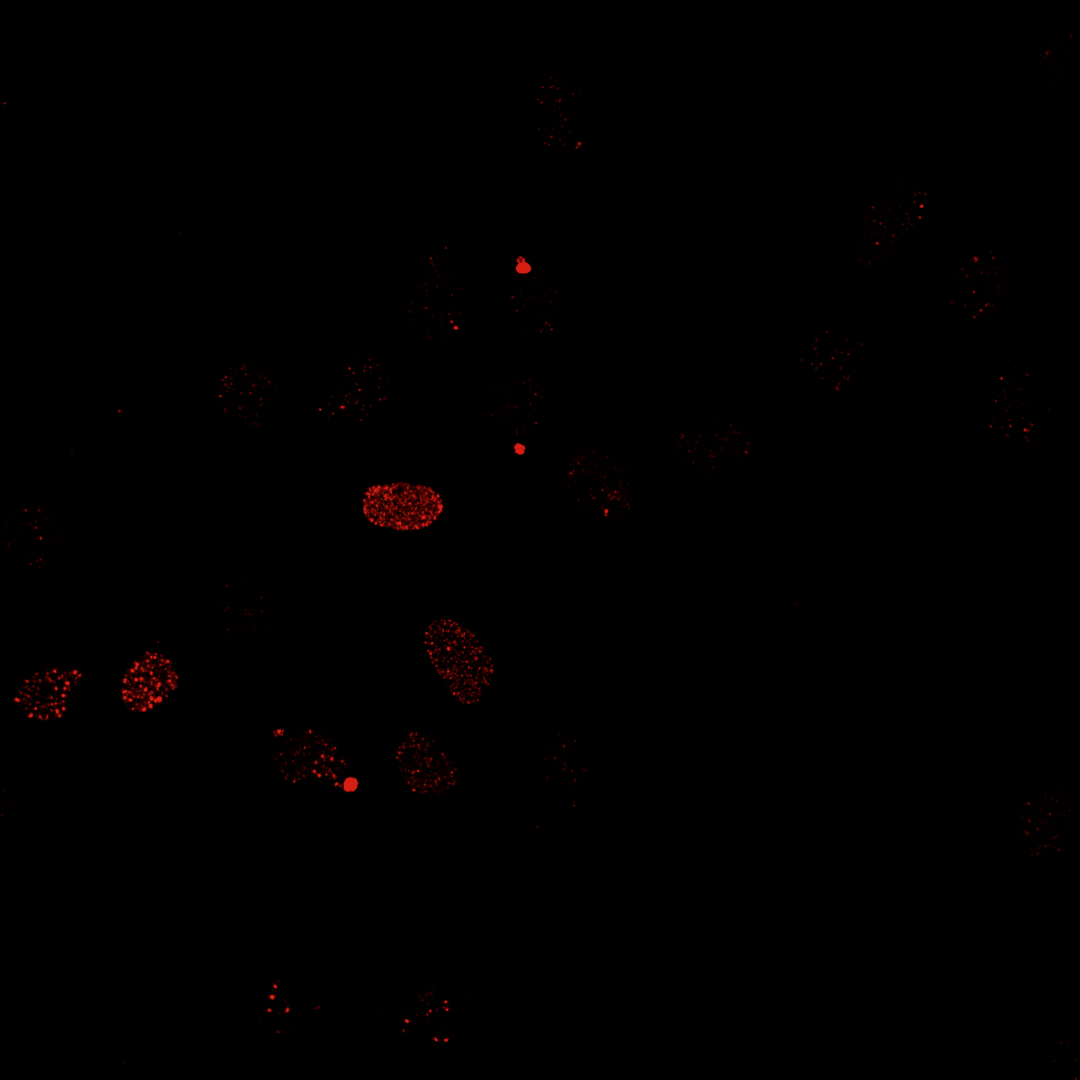

Supplement: Supplementary file 19 — Figure EV4 Source Data [file 44321_2025_352_MOESM19_ESM.zip › EMM-2025-21907-V2_SourceDataFigEV4/EV4F/MAEA M396R gH2AX ATRi.png]

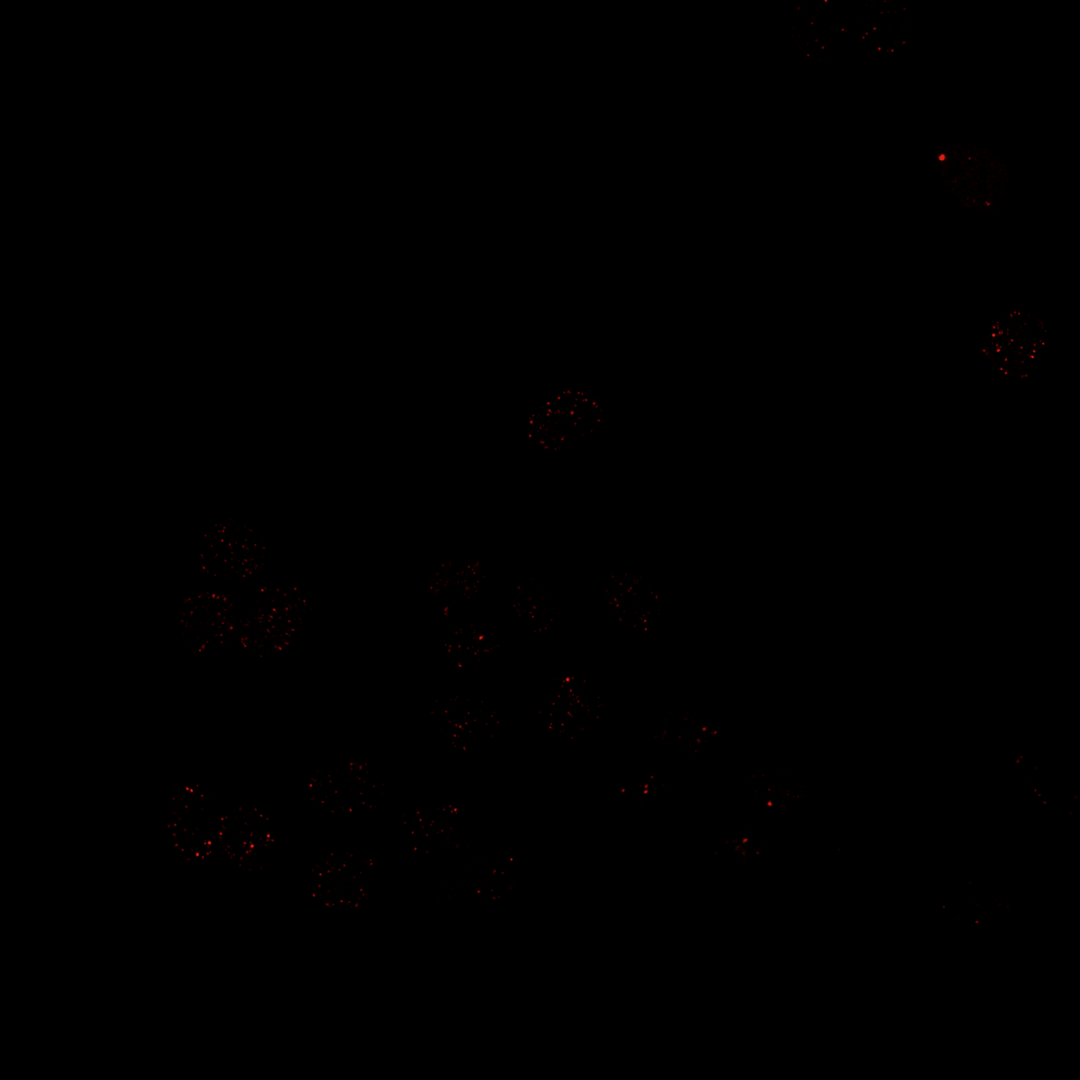

Supplement: Supplementary file 19 — Figure EV4 Source Data [file 44321_2025_352_MOESM19_ESM.zip › EMM-2025-21907-V2_SourceDataFigEV4/EV4F/MAEA 3S gH2AX UT.png]

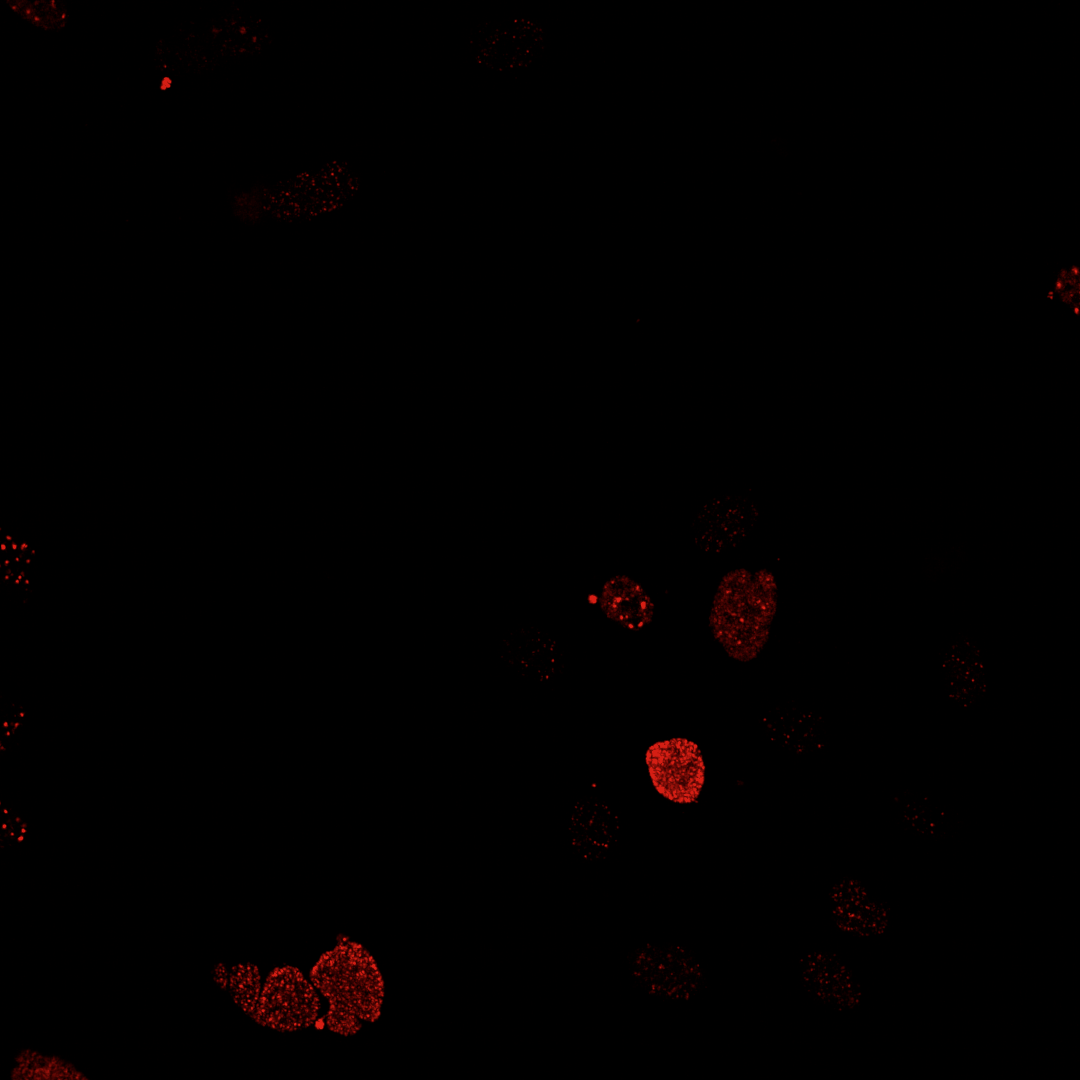

Supplement: Supplementary file 19 — Figure EV4 Source Data [file 44321_2025_352_MOESM19_ESM.zip › EMM-2025-21907-V2_SourceDataFigEV4/EV4F/MAEA E349K gH2AX ATRi.png]

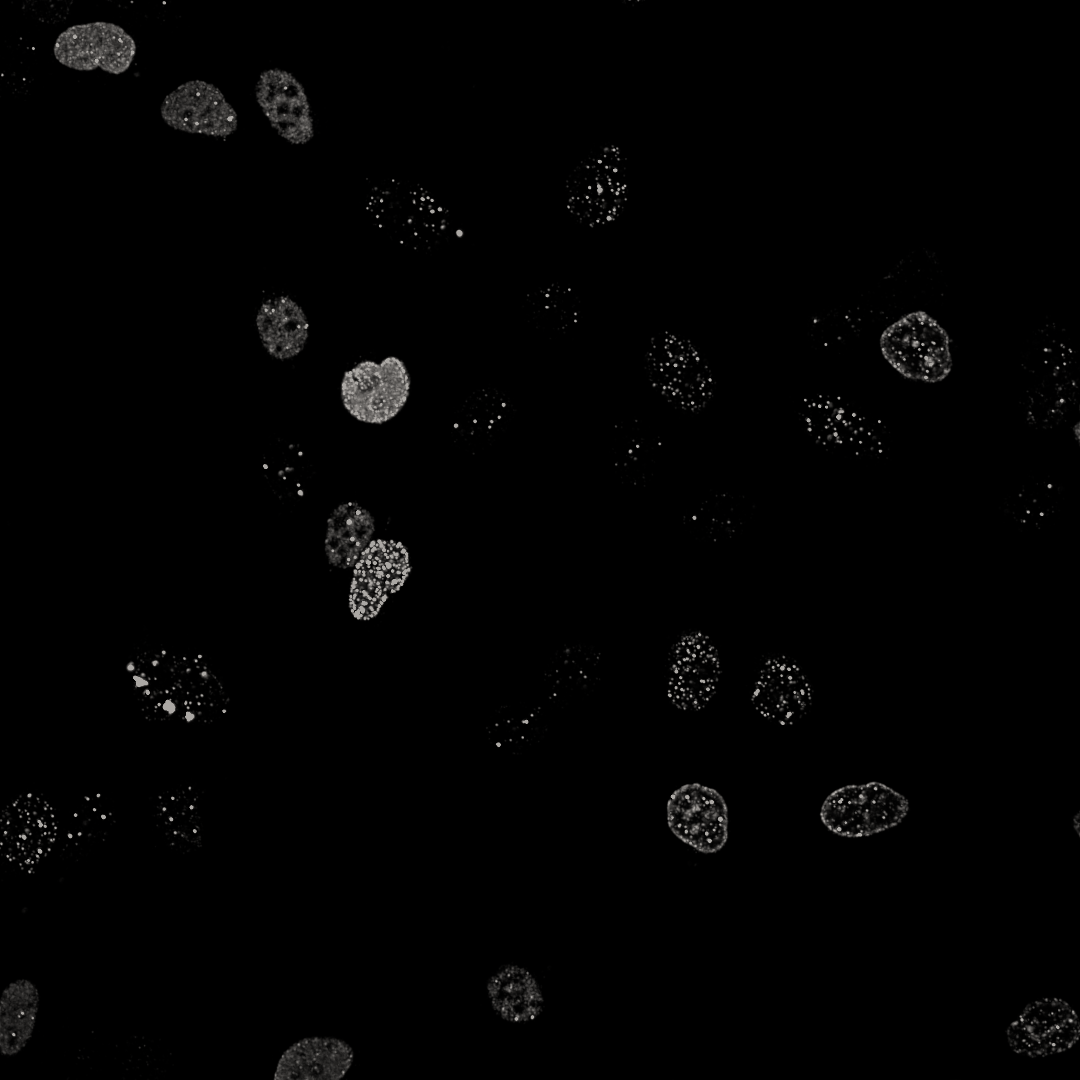

Supplement: Supplementary file 19 — Figure EV4 Source Data [file 44321_2025_352_MOESM19_ESM.zip › EMM-2025-21907-V2_SourceDataFigEV4/EV4F/GFP only RPA ATRi.png]

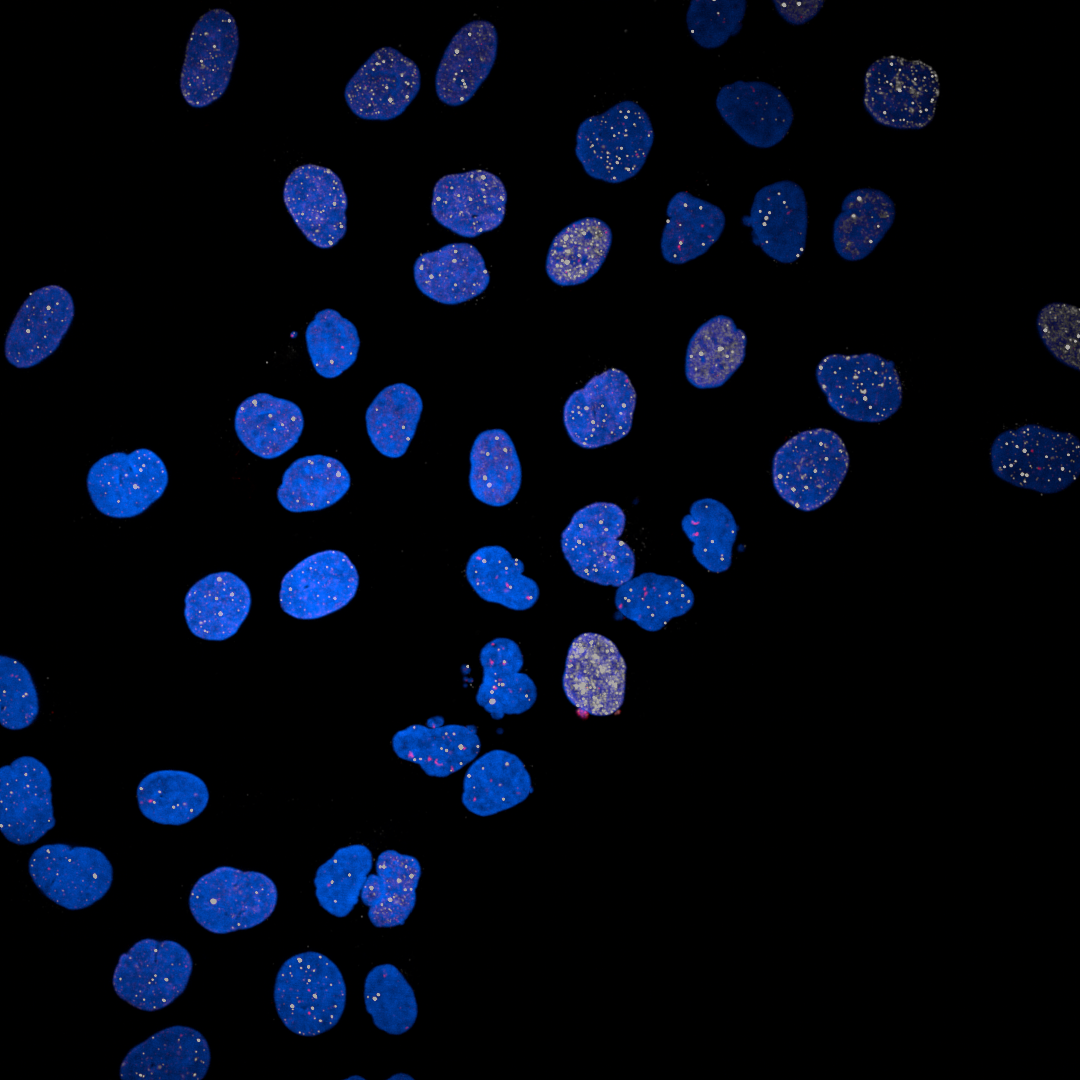

Supplement: Supplementary file 19 — Figure EV4 Source Data [file 44321_2025_352_MOESM19_ESM.zip › EMM-2025-21907-V2_SourceDataFigEV4/EV4F/WT merged ATRi.png]

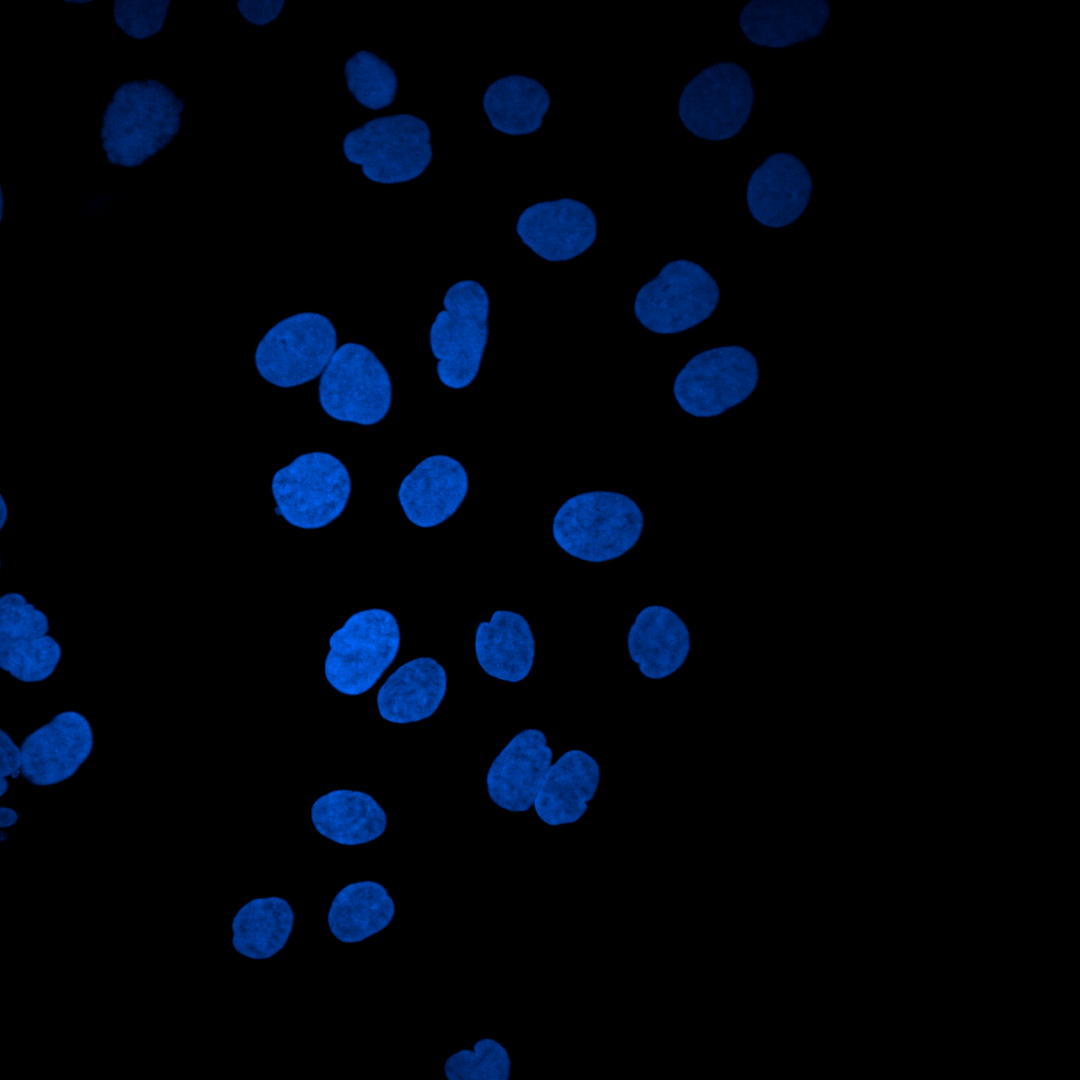

Supplement: Supplementary file 19 — Figure EV4 Source Data [file 44321_2025_352_MOESM19_ESM.zip › EMM-2025-21907-V2_SourceDataFigEV4/EV4F/MAEA E349K DAPI UT.png]
