# Supplementary material for: Loss of CTLH component MAEA impairs DNA repair and replication and leads to developmental delay
Source: EMBO Mol Med. 2025 Dec 19;18(2):492–513. doi: 10.1038/s44321-025-00352-x (PMC12905269; doi:10.1038/s44321-025-00352-x)
Supplement: Supplementary file 19 — Figure EV4 Source Data [file 44321_2025_352_MOESM19_ESM.zip › EMM-2025-21907-V2_SourceDataFigEV4/EV4E/README_4VE.rtf]

This file contains the raw images for RAD51 IF staining in SH-SY5Y cells. - WT indicates Wildtype and M indicates MAEA KO cells. - DMSO and CPT denote the treatment conditions. - DAPI indicates the nuclear stain. Alexa 488 indicates the RAD51 staining. Alexa 568 indicates PCNA/CENPF staining. Images were assigned a channel and cropped using adobe illustrator to make the figures.
