# Supplementary figures and images for: Loss of CTLH component MAEA impairs DNA repair and replication and leads to developmental delay (part 2 of 2)
Source: EMBO Mol Med. 2025 Dec 19;18(2):492–513. doi: 10.1038/s44321-025-00352-x (PMC12905269; doi:10.1038/s44321-025-00352-x)

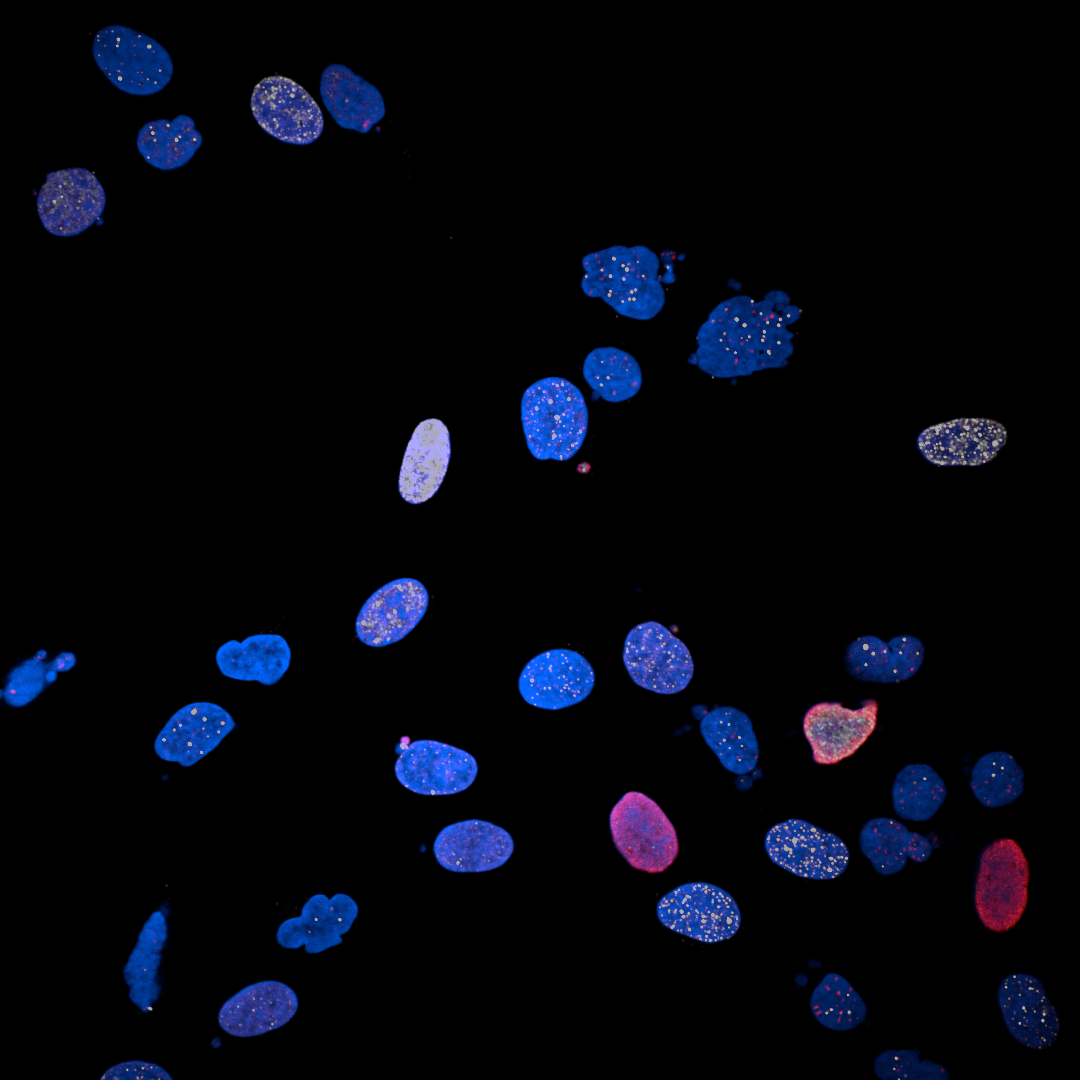

Supplement: Supplementary file 19 — Figure EV4 Source Data [file 44321_2025_352_MOESM19_ESM.zip › EMM-2025-21907-V2_SourceDataFigEV4/EV4F/MAEA 3S merged ATRi.png]

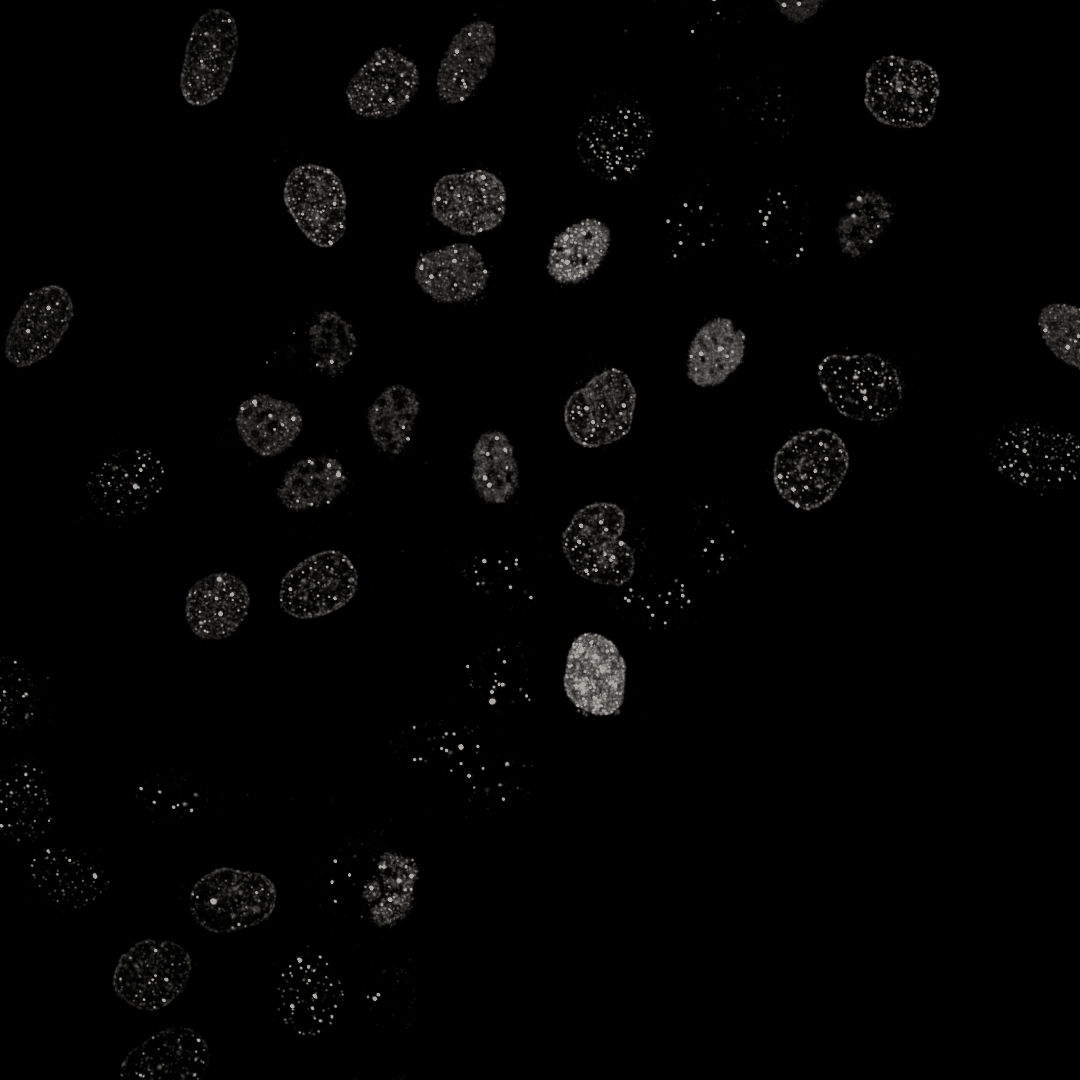

Supplement: Supplementary file 19 — Figure EV4 Source Data [file 44321_2025_352_MOESM19_ESM.zip › EMM-2025-21907-V2_SourceDataFigEV4/EV4F/WT RPA ATRi.png]

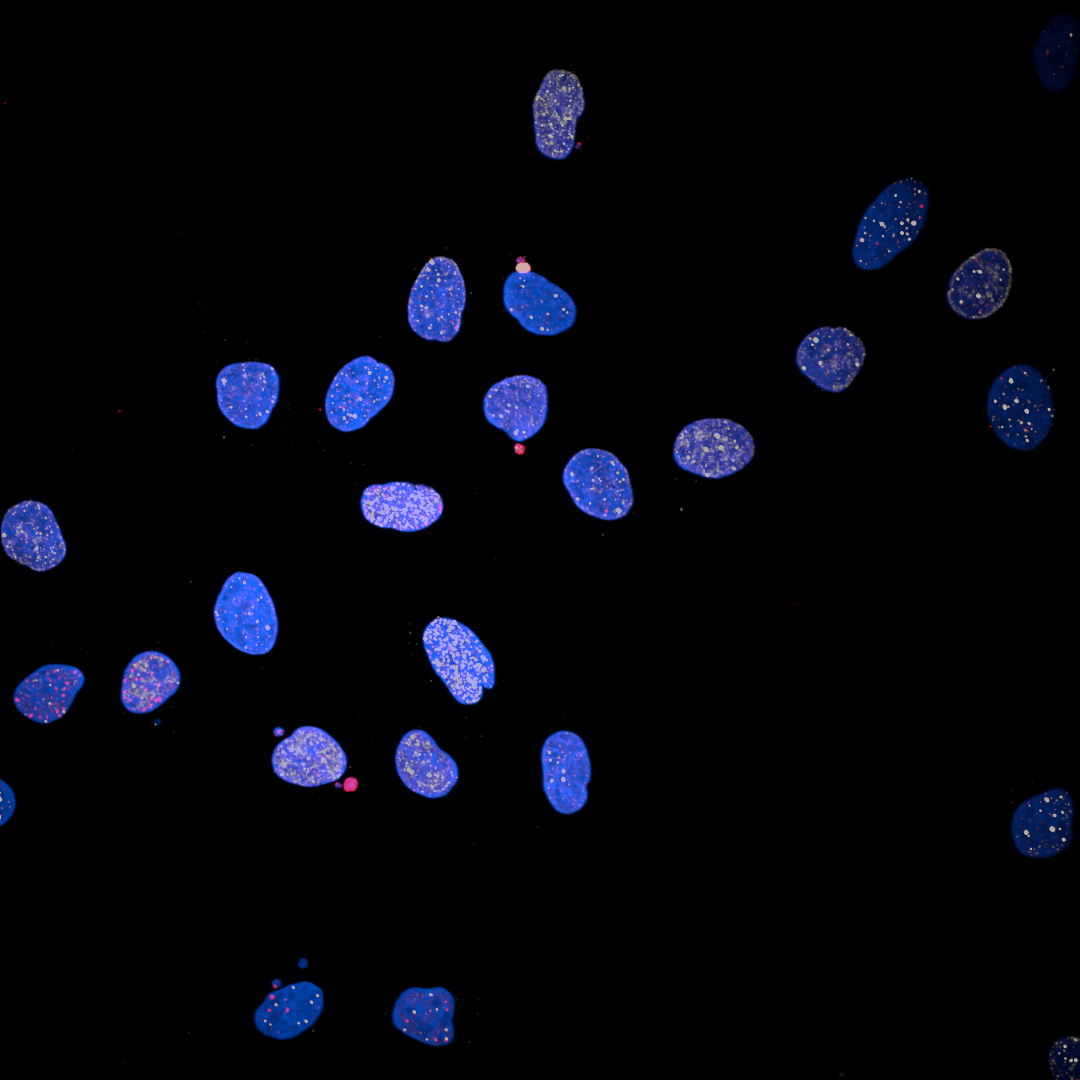

Supplement: Supplementary file 19 — Figure EV4 Source Data [file 44321_2025_352_MOESM19_ESM.zip › EMM-2025-21907-V2_SourceDataFigEV4/EV4F/MAEA M396R merged ATRi.png]

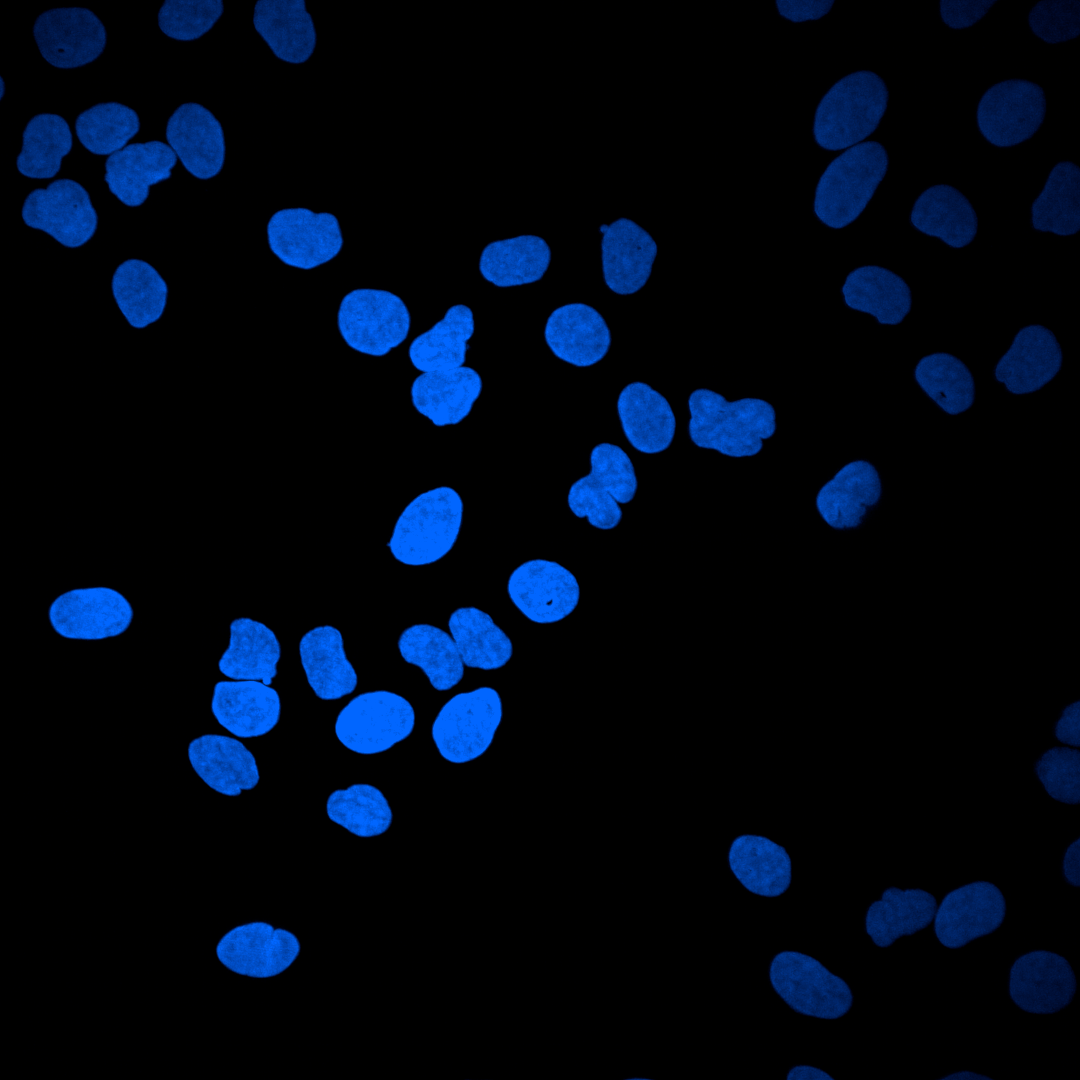

Supplement: Supplementary file 19 — Figure EV4 Source Data [file 44321_2025_352_MOESM19_ESM.zip › EMM-2025-21907-V2_SourceDataFigEV4/EV4F/WT DAPI UT.png]

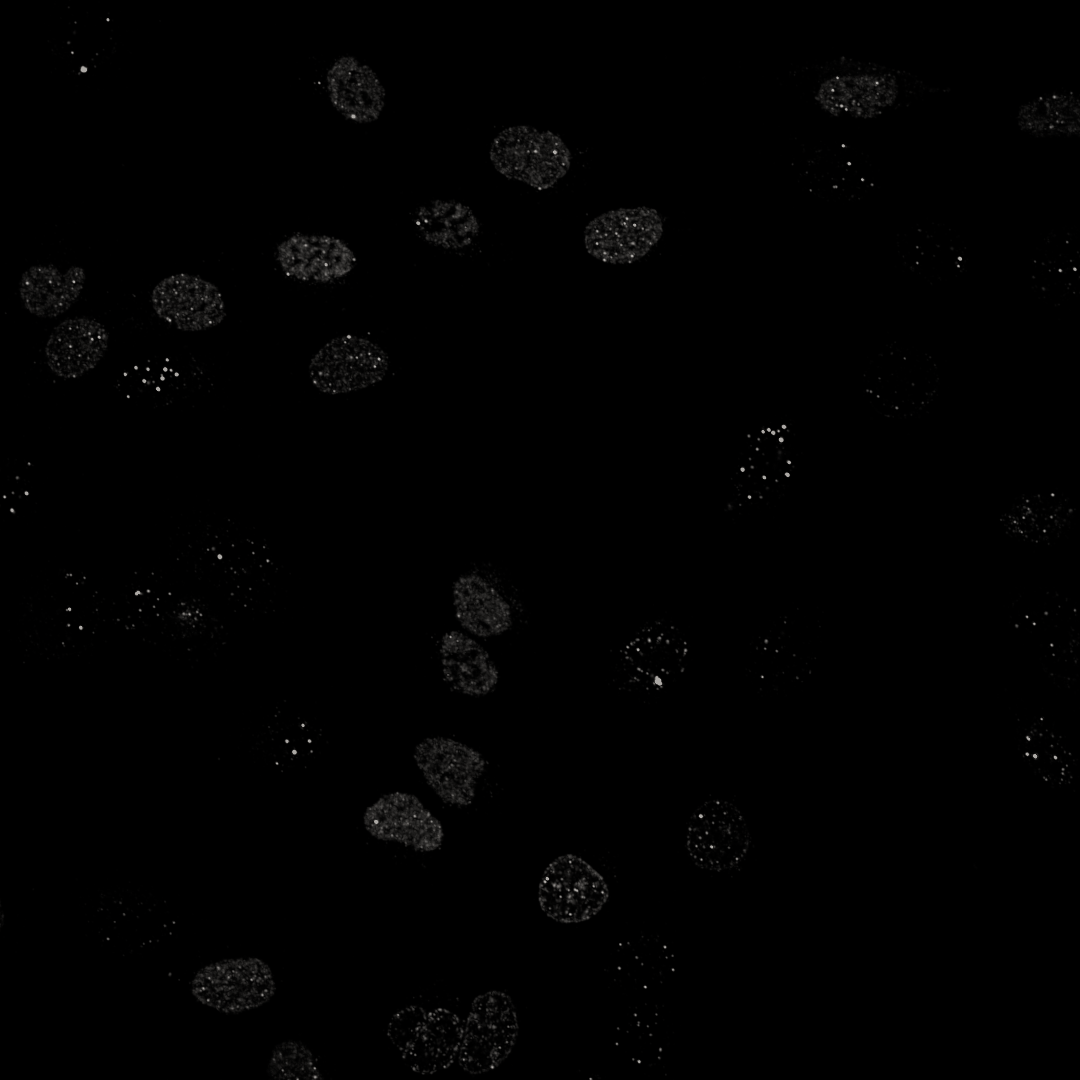

Supplement: Supplementary file 19 — Figure EV4 Source Data [file 44321_2025_352_MOESM19_ESM.zip › EMM-2025-21907-V2_SourceDataFigEV4/EV4F/MAEA M396R RPA UT.png]

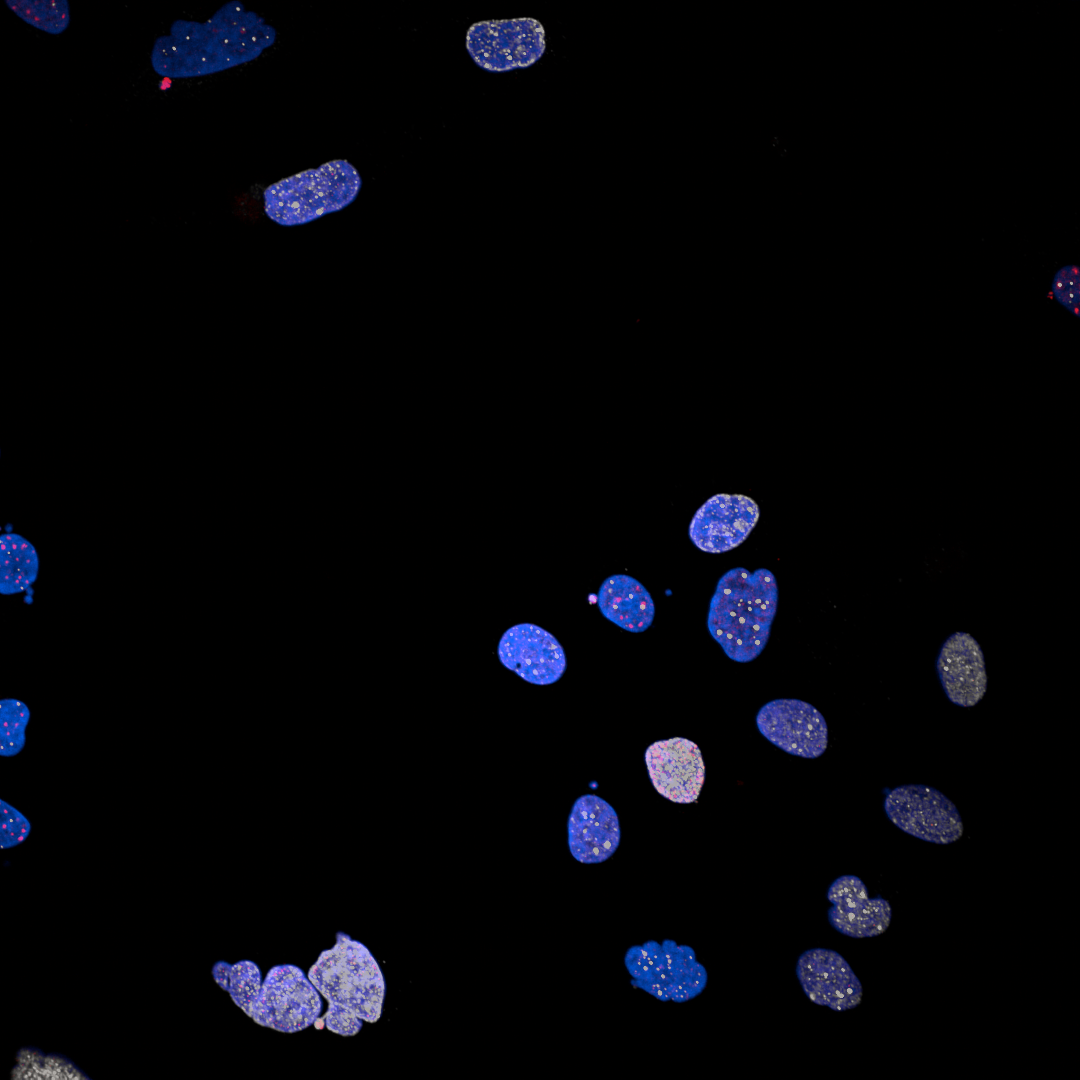

Supplement: Supplementary file 19 — Figure EV4 Source Data [file 44321_2025_352_MOESM19_ESM.zip › EMM-2025-21907-V2_SourceDataFigEV4/EV4F/MAEA E349K merged ATRi.png]

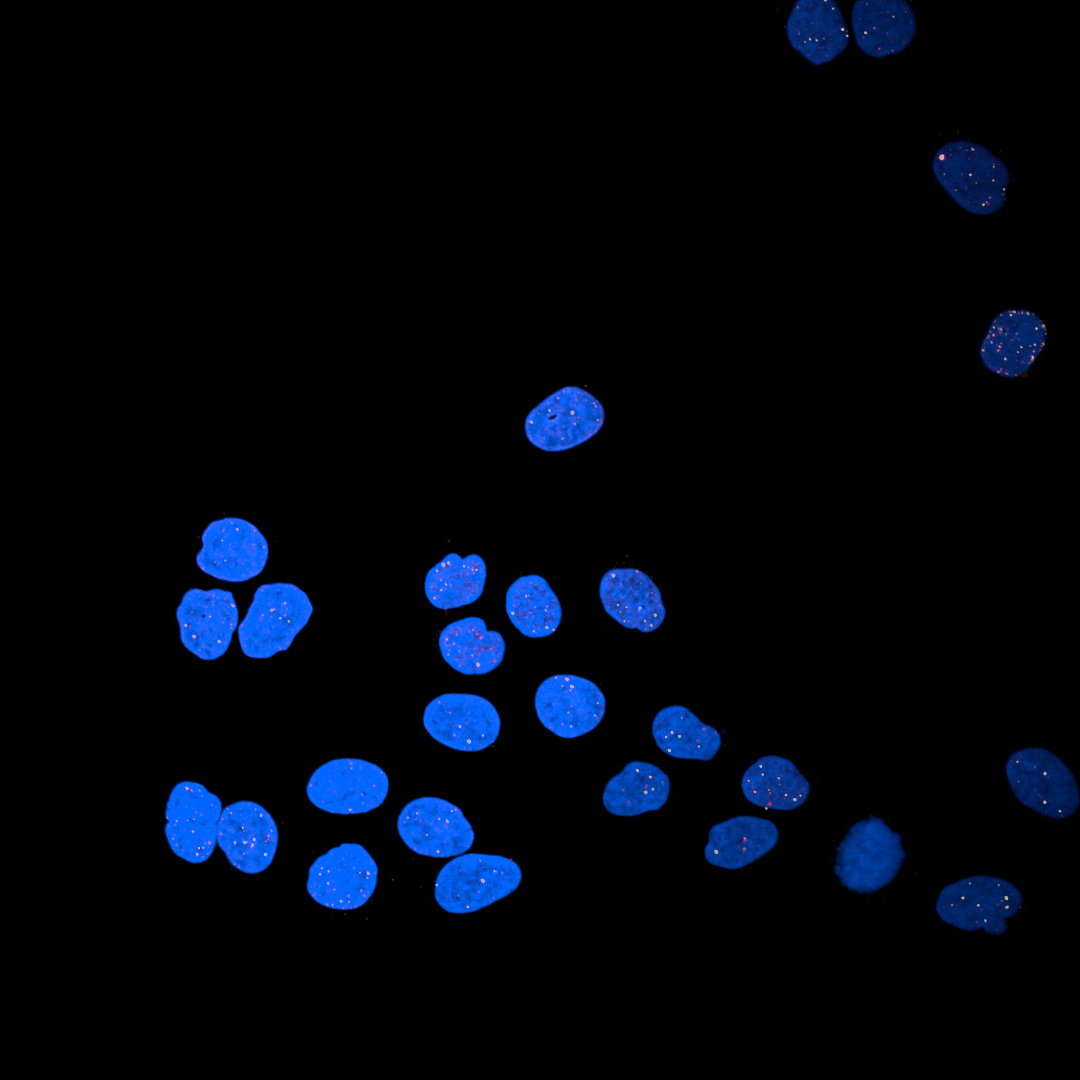

Supplement: Supplementary file 19 — Figure EV4 Source Data [file 44321_2025_352_MOESM19_ESM.zip › EMM-2025-21907-V2_SourceDataFigEV4/EV4F/MAEA 3S merged UT.png]

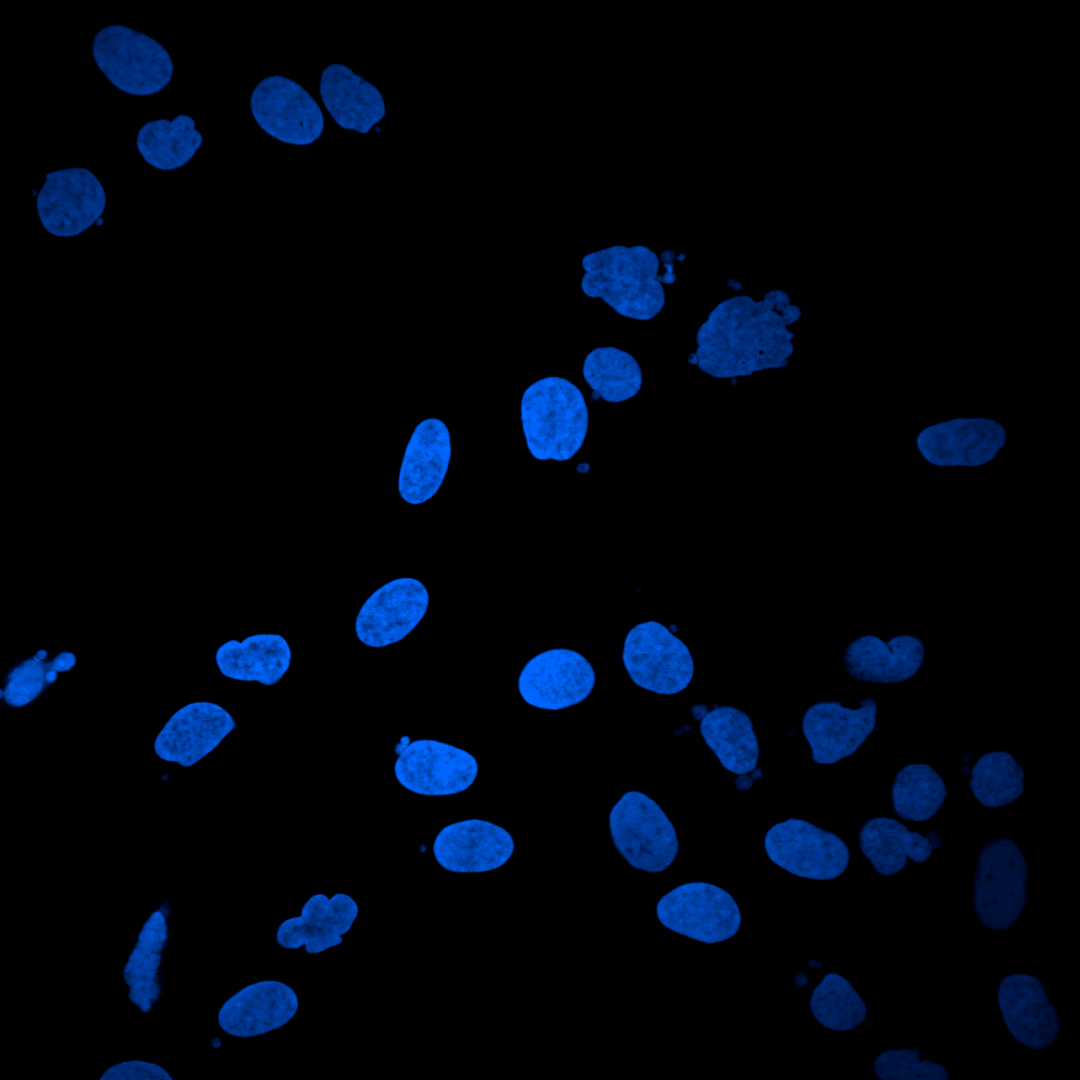

Supplement: Supplementary file 19 — Figure EV4 Source Data [file 44321_2025_352_MOESM19_ESM.zip › EMM-2025-21907-V2_SourceDataFigEV4/EV4F/MAEA 3S DAPI ATRi.png]

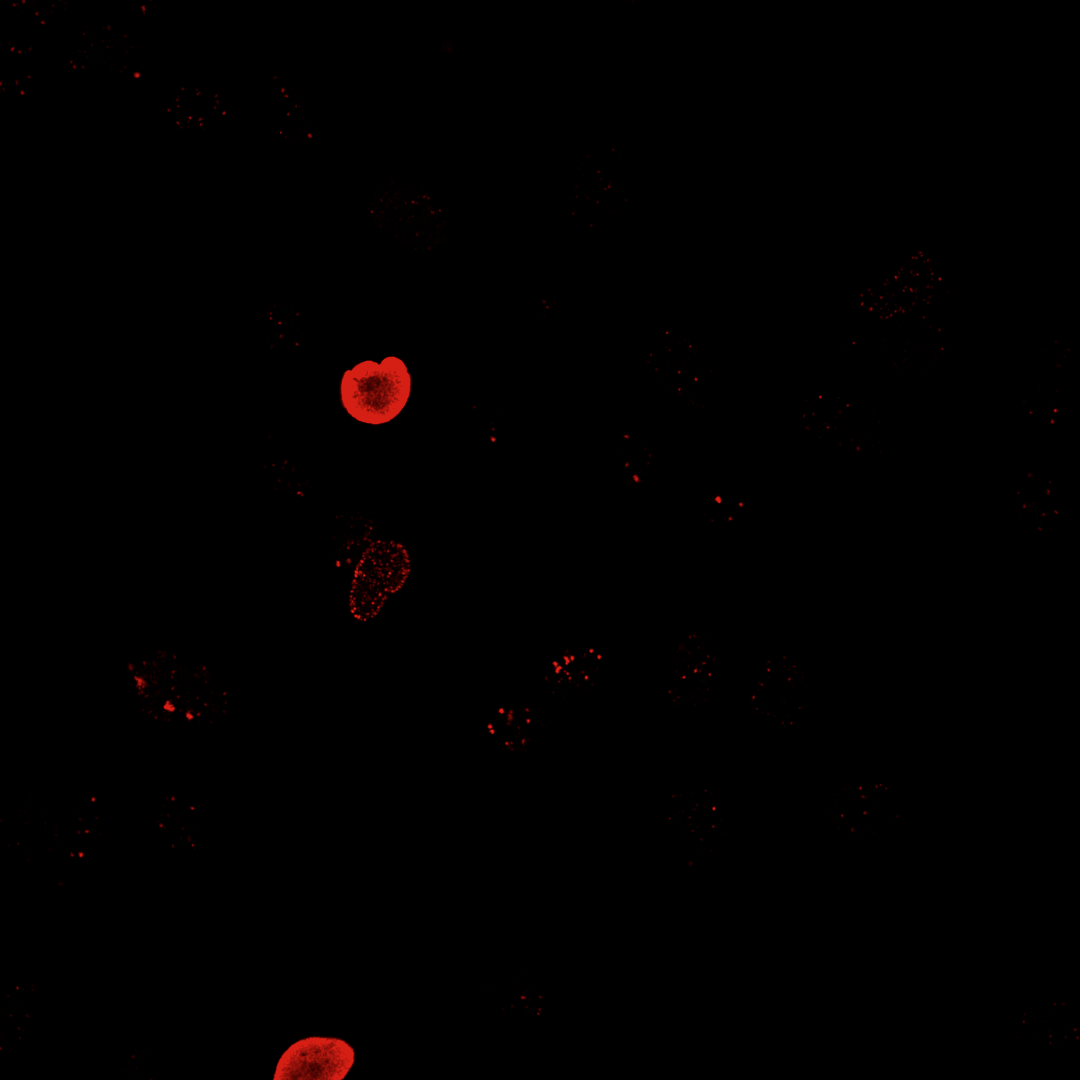

Supplement: Supplementary file 19 — Figure EV4 Source Data [file 44321_2025_352_MOESM19_ESM.zip › EMM-2025-21907-V2_SourceDataFigEV4/EV4F/GFP only gH2AX ATRi.png]

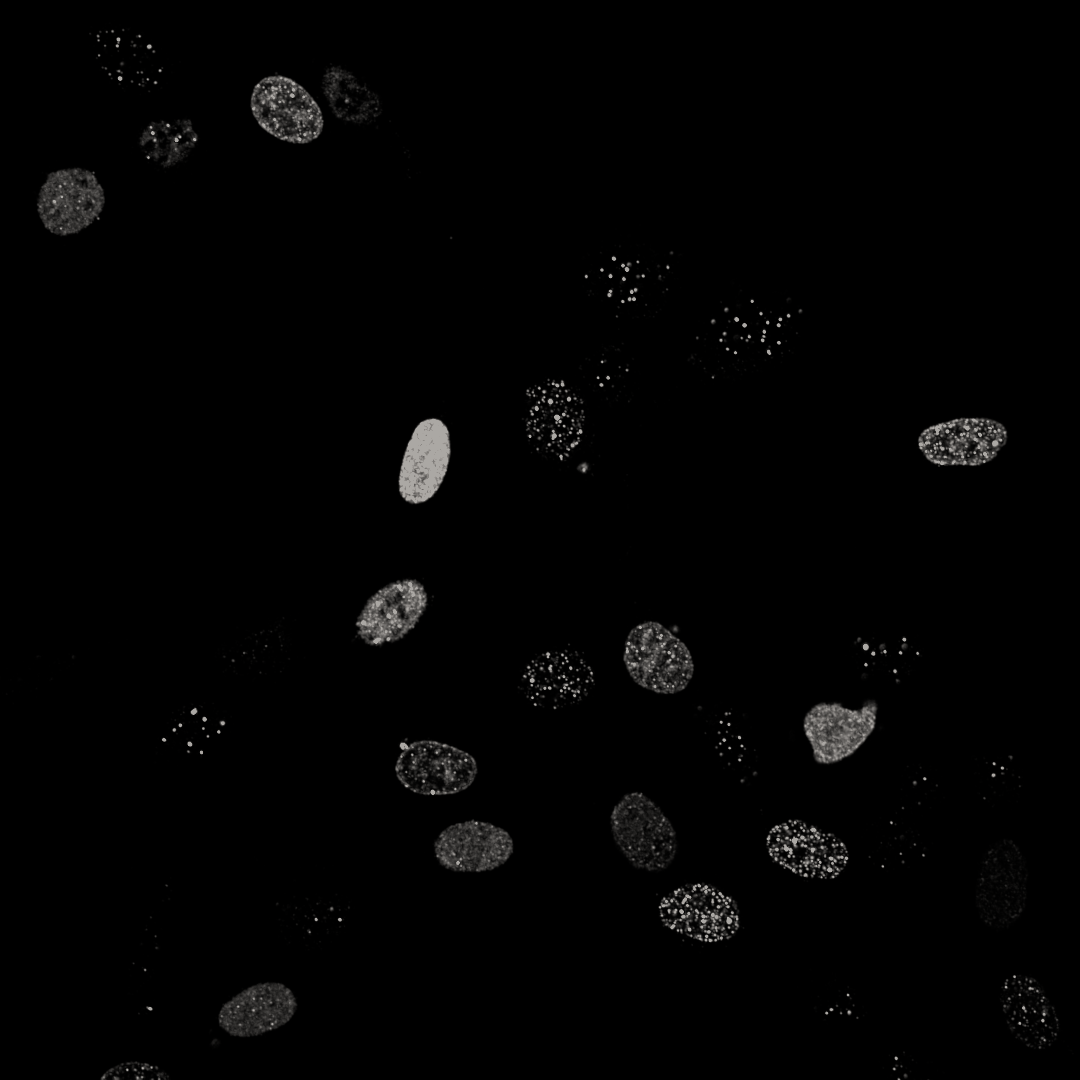

Supplement: Supplementary file 19 — Figure EV4 Source Data [file 44321_2025_352_MOESM19_ESM.zip › EMM-2025-21907-V2_SourceDataFigEV4/EV4F/MAEA 3S RPA ATRi.png]

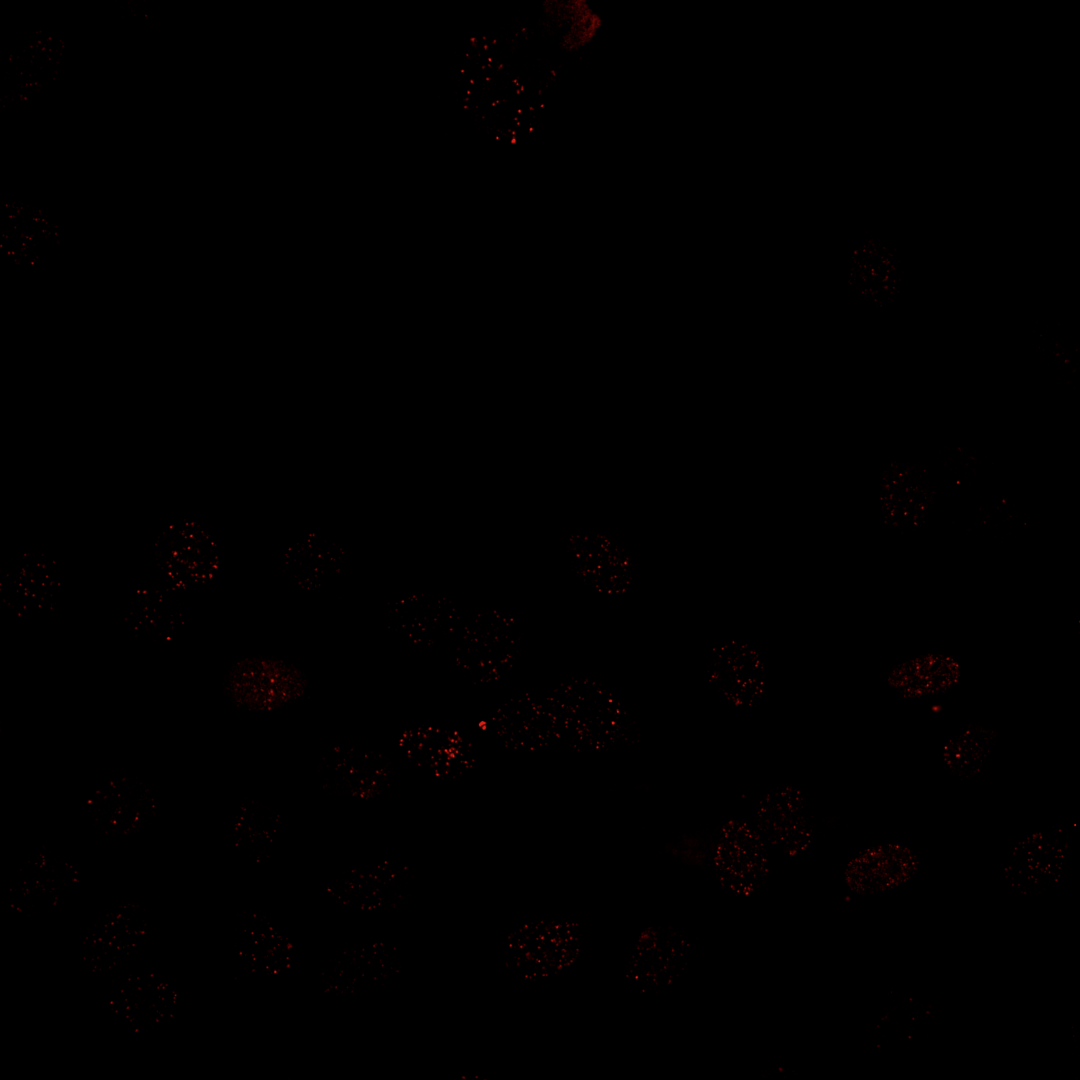

Supplement: Supplementary file 19 — Figure EV4 Source Data [file 44321_2025_352_MOESM19_ESM.zip › EMM-2025-21907-V2_SourceDataFigEV4/EV4F/GFP only gH2AX UT.png]

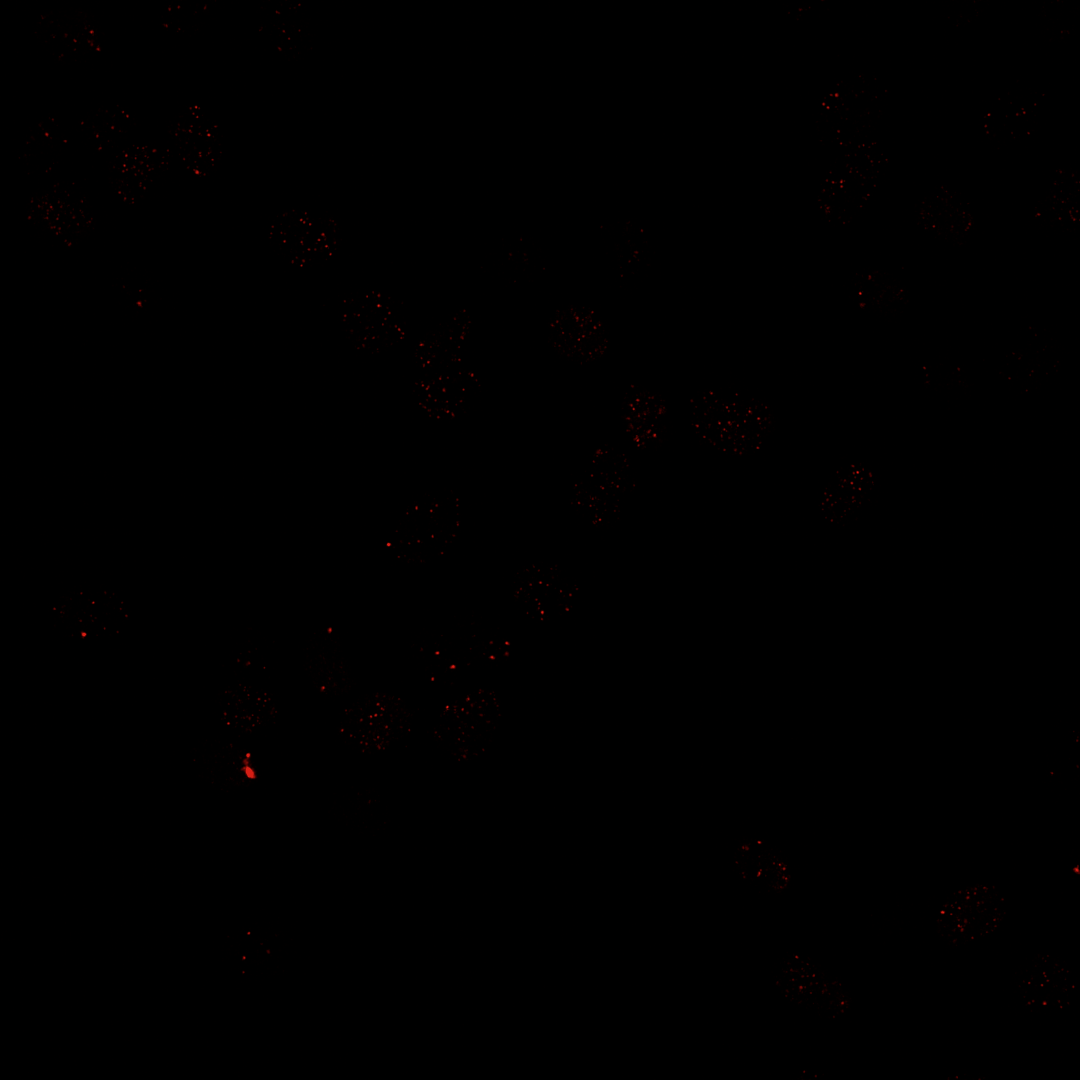

Supplement: Supplementary file 19 — Figure EV4 Source Data [file 44321_2025_352_MOESM19_ESM.zip › EMM-2025-21907-V2_SourceDataFigEV4/EV4F/WT gH2AX UT.png]

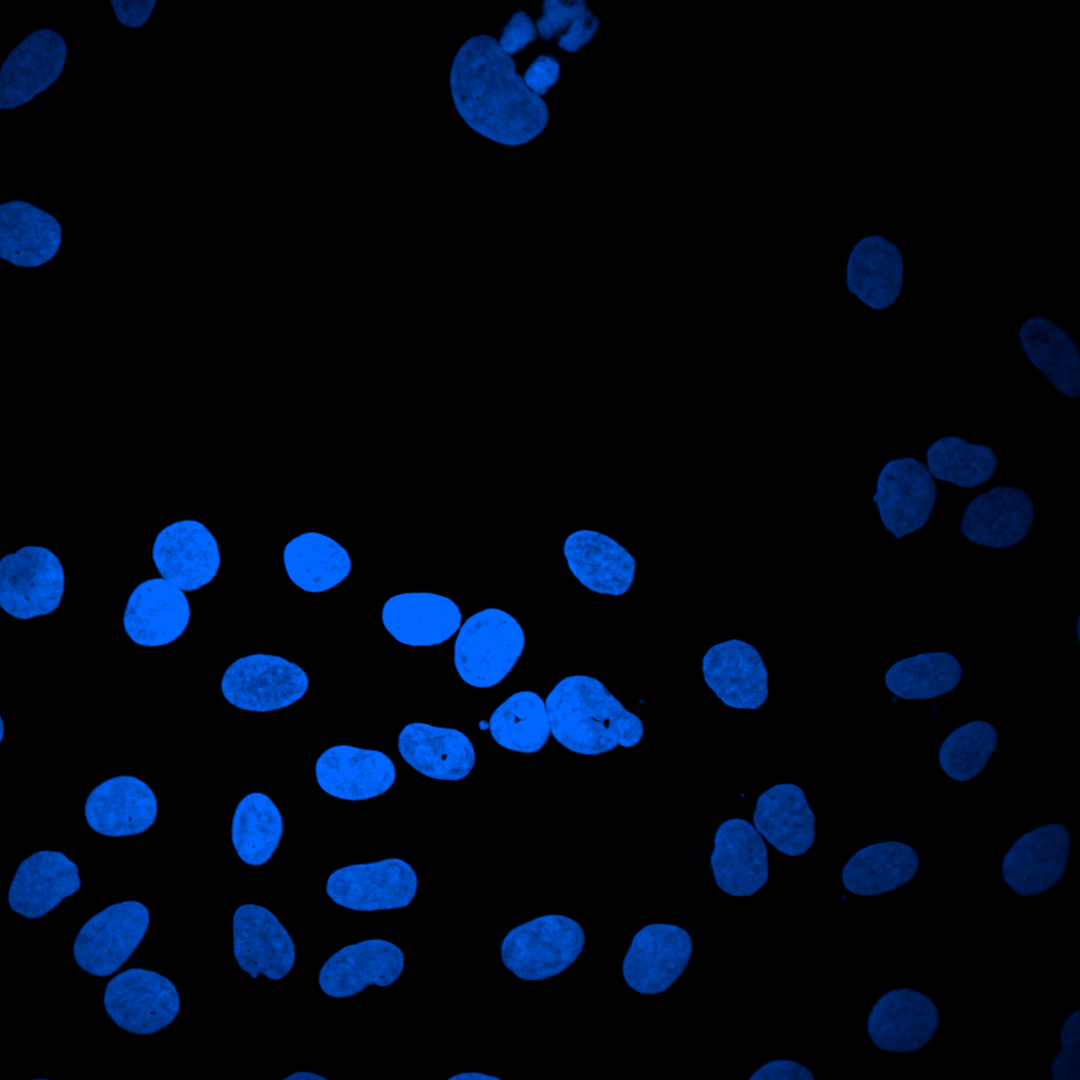

Supplement: Supplementary file 19 — Figure EV4 Source Data [file 44321_2025_352_MOESM19_ESM.zip › EMM-2025-21907-V2_SourceDataFigEV4/EV4F/GFP only DAPI UT.png]

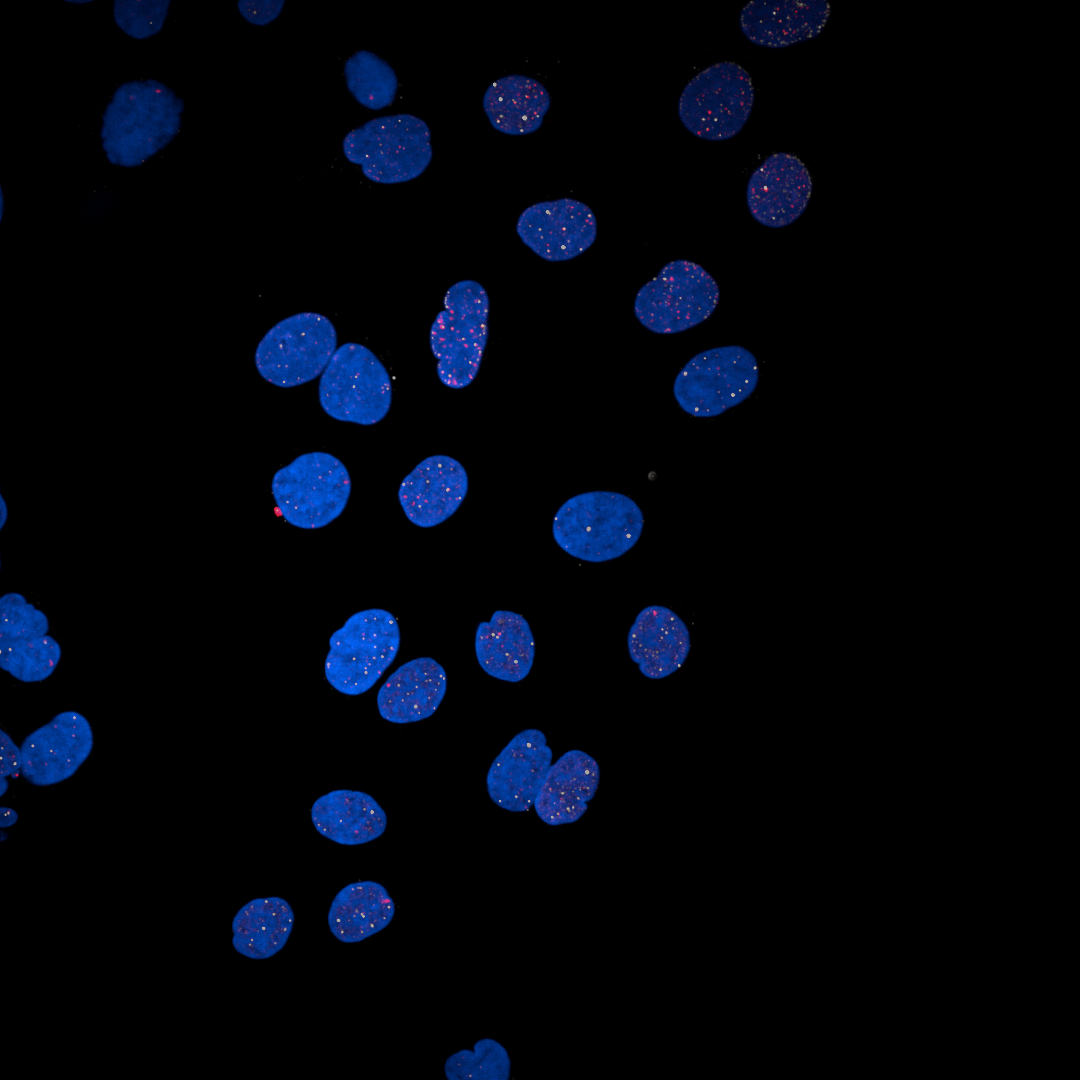

Supplement: Supplementary file 19 — Figure EV4 Source Data [file 44321_2025_352_MOESM19_ESM.zip › EMM-2025-21907-V2_SourceDataFigEV4/EV4F/MAEA E349K merged UT.png]

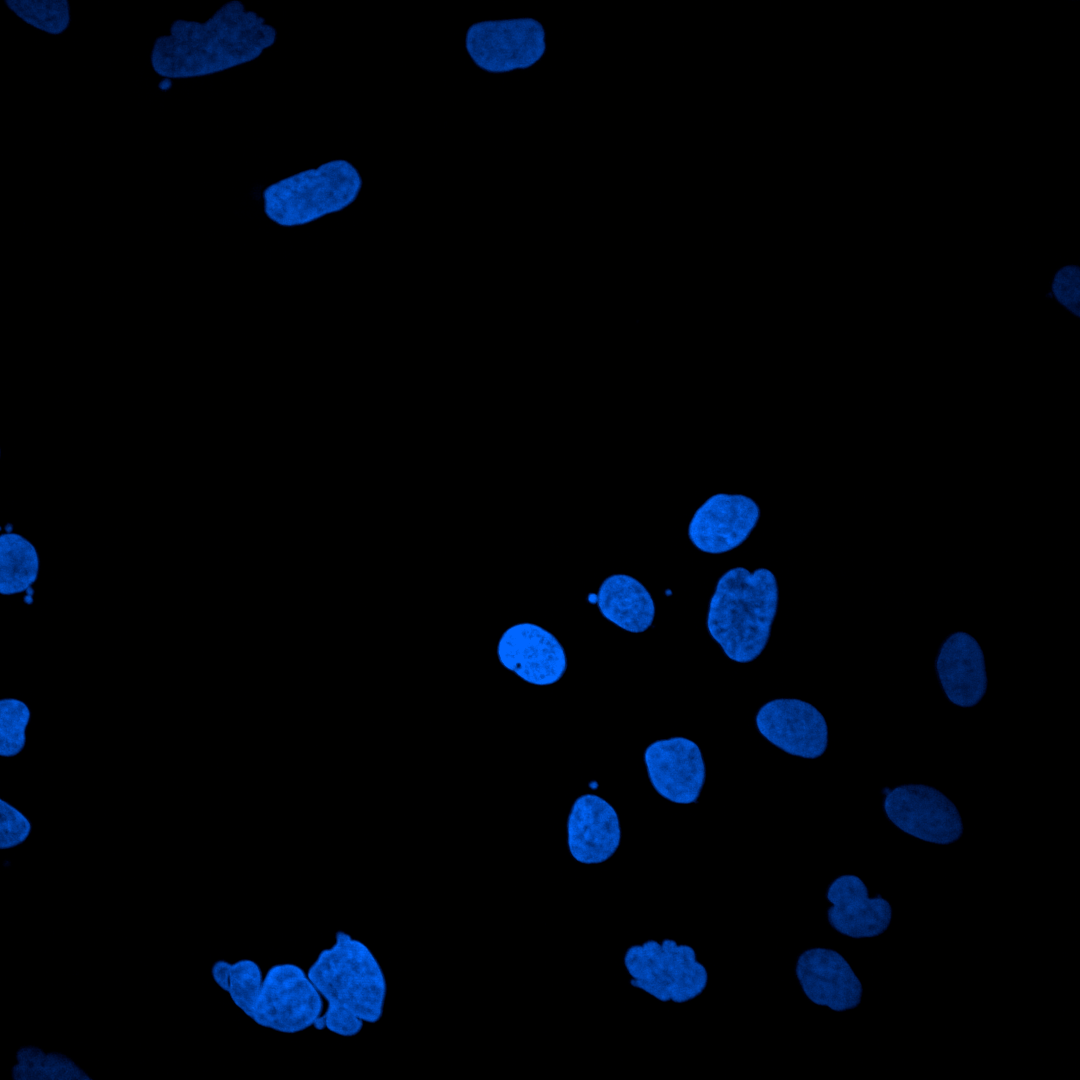

Supplement: Supplementary file 19 — Figure EV4 Source Data [file 44321_2025_352_MOESM19_ESM.zip › EMM-2025-21907-V2_SourceDataFigEV4/EV4F/MAEA E349K DAPI ATRi.png]

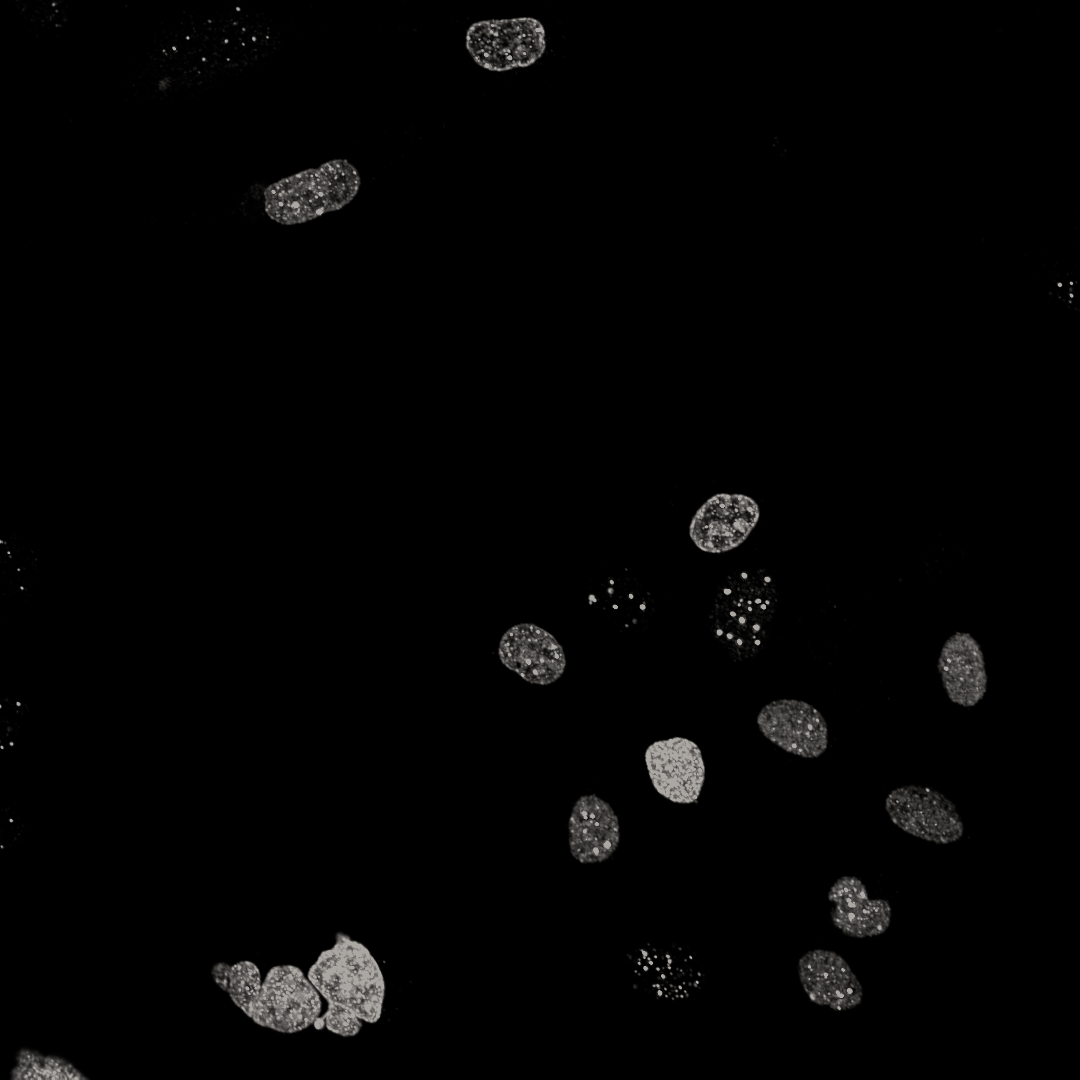

Supplement: Supplementary file 19 — Figure EV4 Source Data [file 44321_2025_352_MOESM19_ESM.zip › EMM-2025-21907-V2_SourceDataFigEV4/EV4F/MAEA E349K RPA ATRi.png]

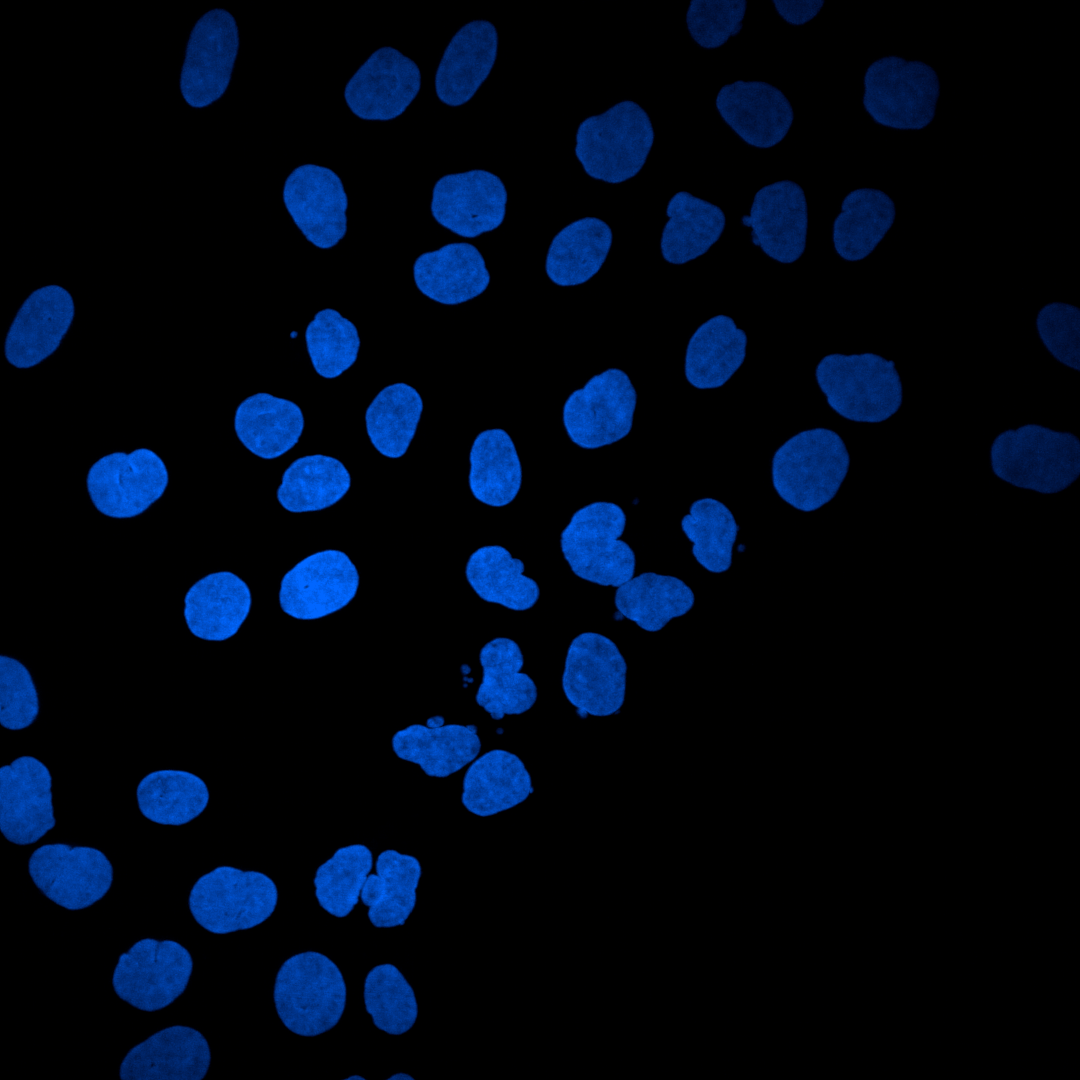

Supplement: Supplementary file 19 — Figure EV4 Source Data [file 44321_2025_352_MOESM19_ESM.zip › EMM-2025-21907-V2_SourceDataFigEV4/EV4F/WT DAPI ATRi.png]

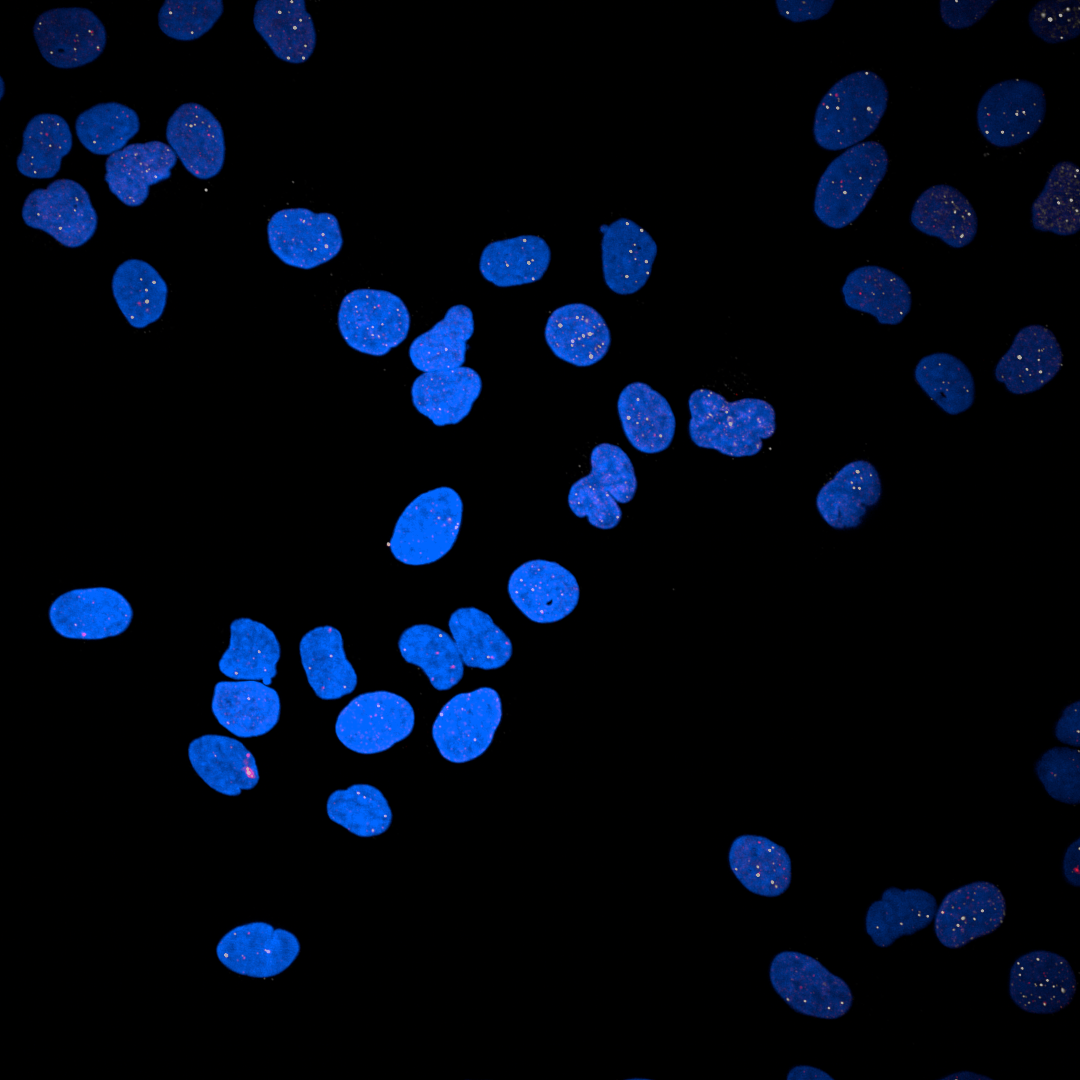

Supplement: Supplementary file 19 — Figure EV4 Source Data [file 44321_2025_352_MOESM19_ESM.zip › EMM-2025-21907-V2_SourceDataFigEV4/EV4F/WT merged UT.png]

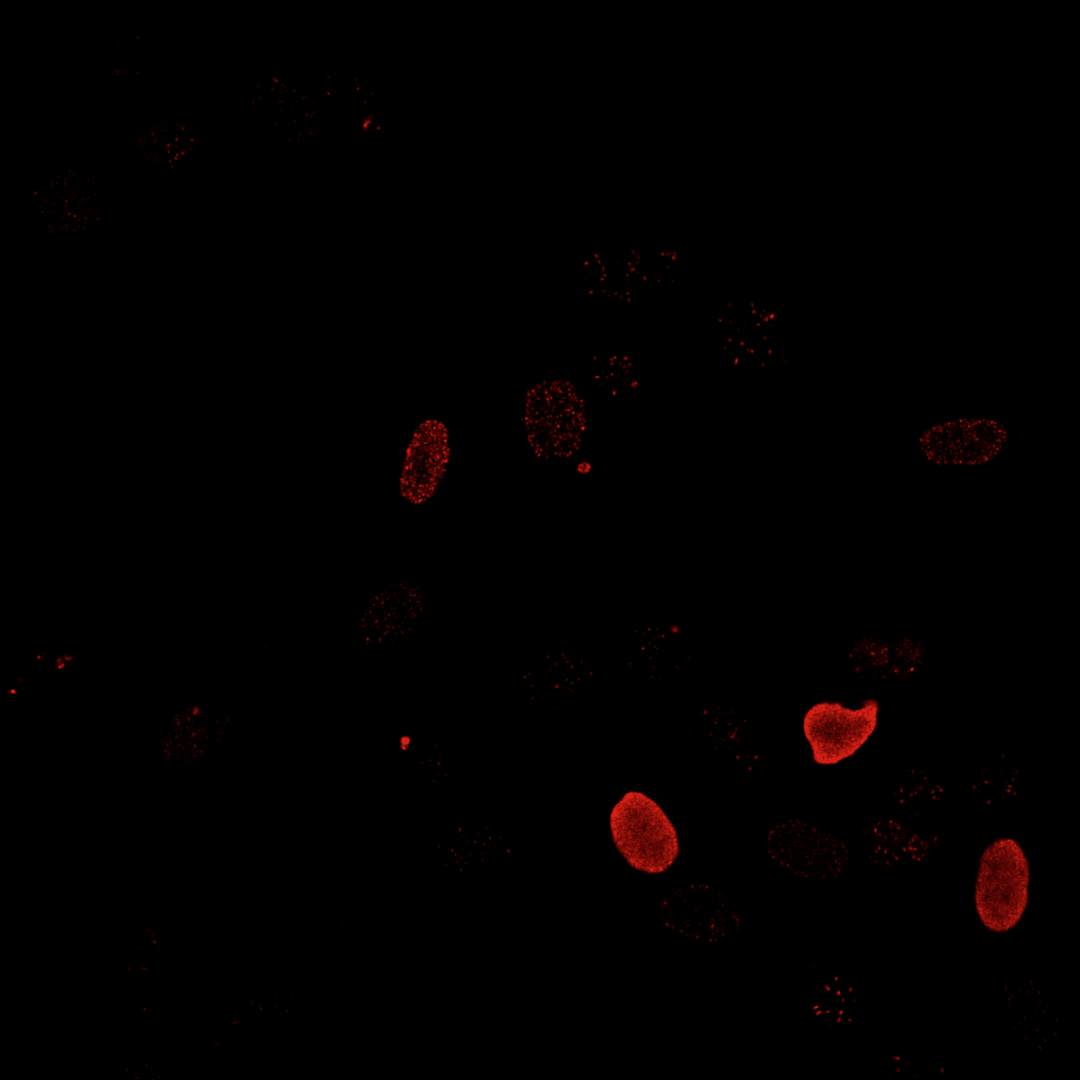

Supplement: Supplementary file 19 — Figure EV4 Source Data [file 44321_2025_352_MOESM19_ESM.zip › EMM-2025-21907-V2_SourceDataFigEV4/EV4F/MAEA 3S gH2AX ATRi.png]

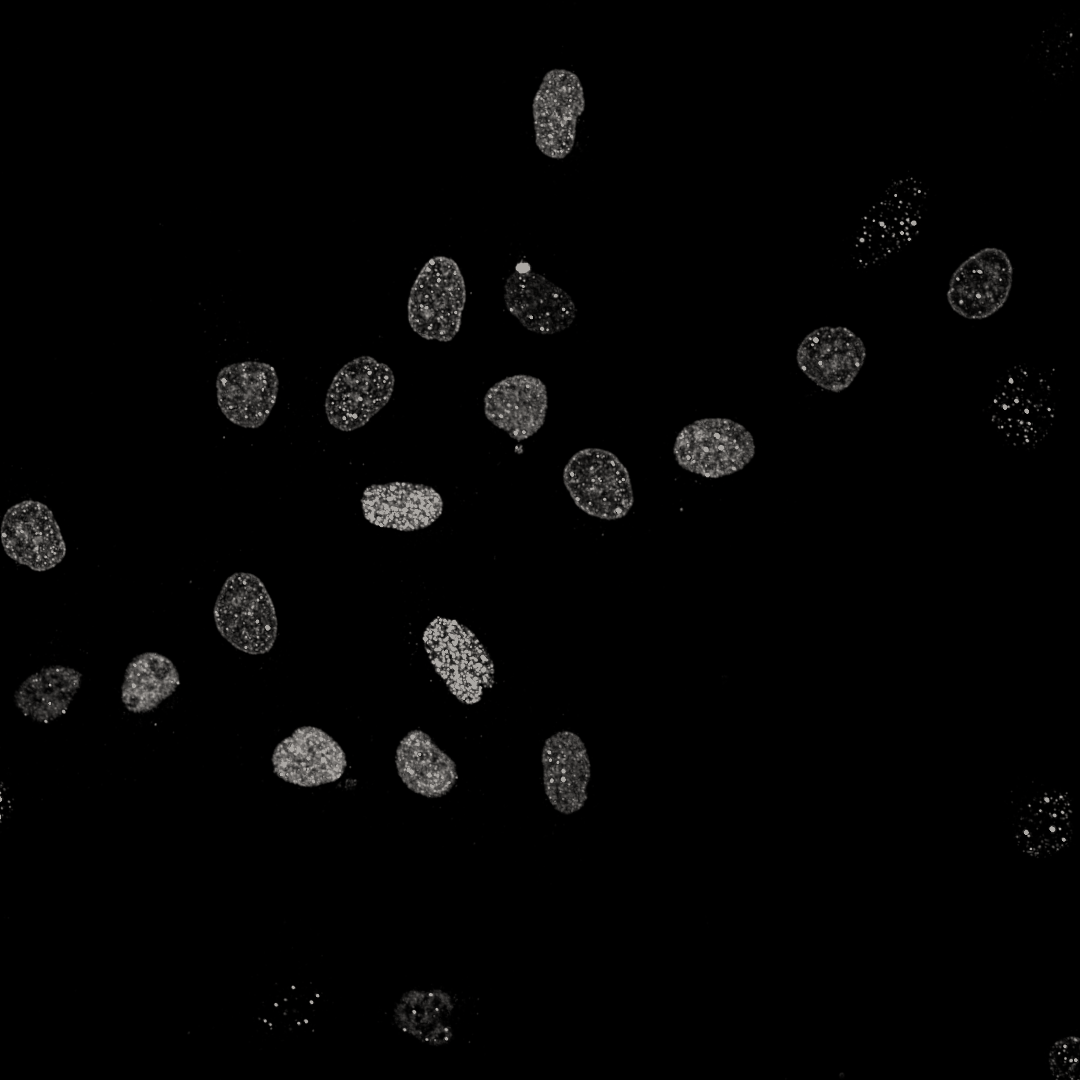

Supplement: Supplementary file 19 — Figure EV4 Source Data [file 44321_2025_352_MOESM19_ESM.zip › EMM-2025-21907-V2_SourceDataFigEV4/EV4F/MAEA M396R RPA ATRi.png]

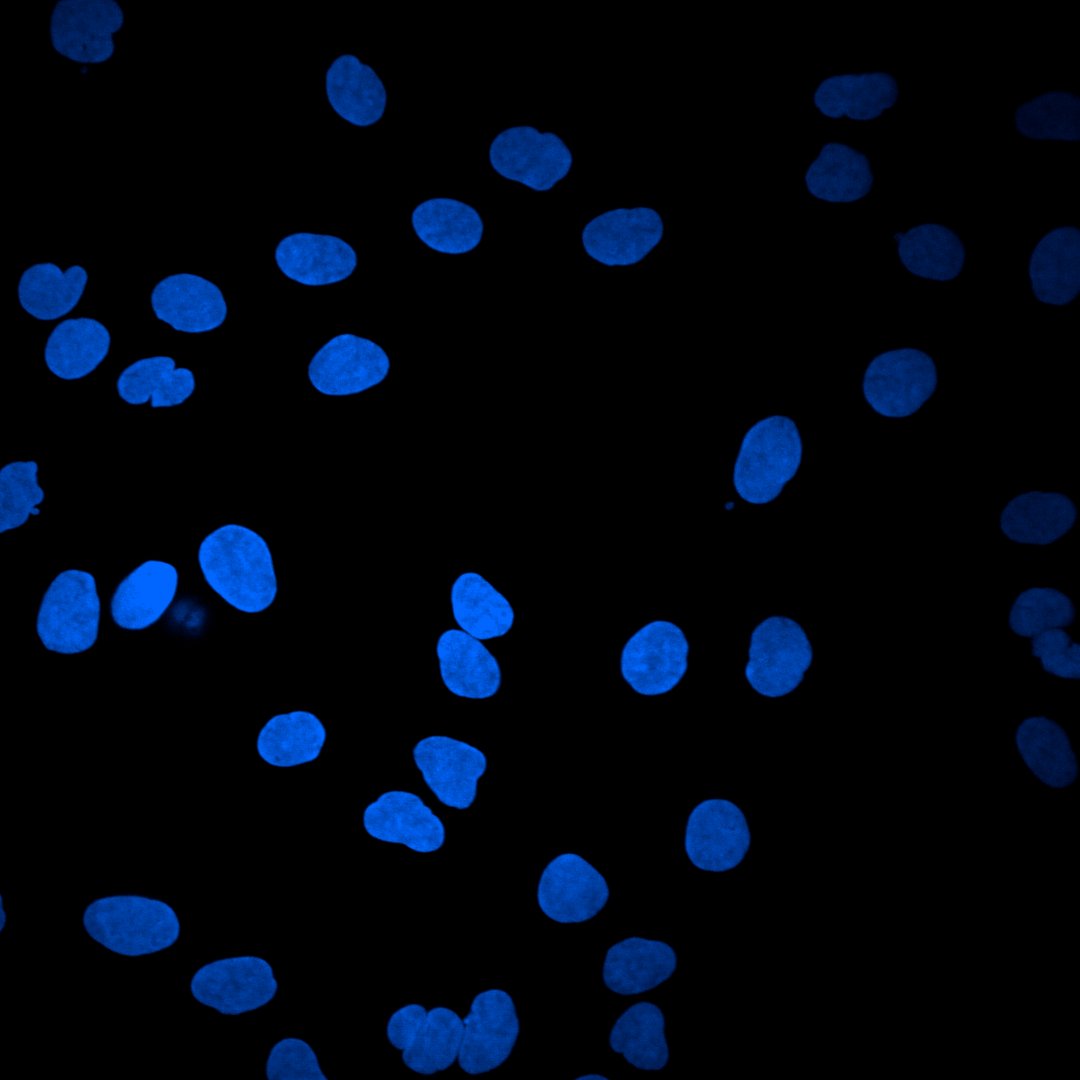

Supplement: Supplementary file 19 — Figure EV4 Source Data [file 44321_2025_352_MOESM19_ESM.zip › EMM-2025-21907-V2_SourceDataFigEV4/EV4F/MAEA M396R DAPI UT.png]

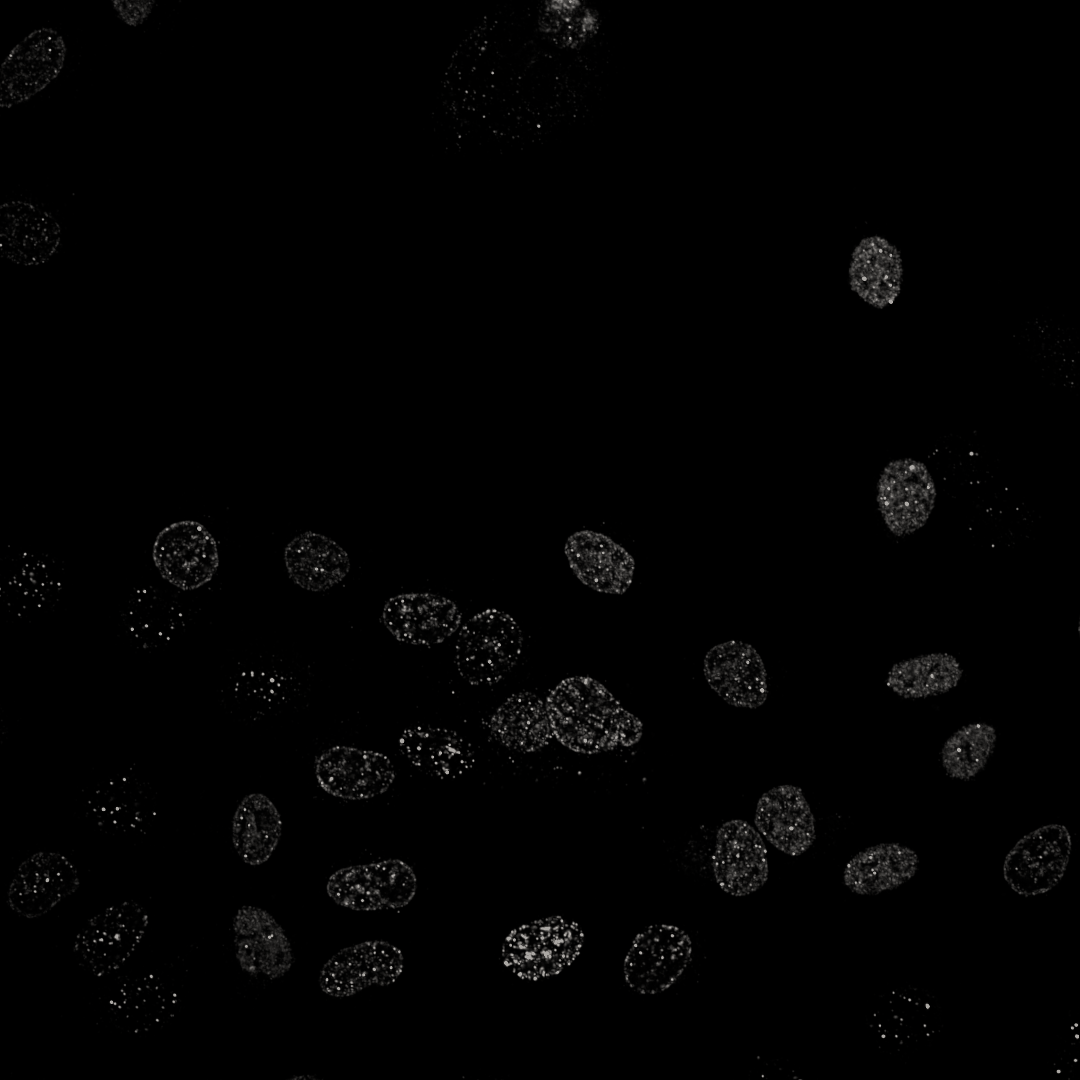

Supplement: Supplementary file 19 — Figure EV4 Source Data [file 44321_2025_352_MOESM19_ESM.zip › EMM-2025-21907-V2_SourceDataFigEV4/EV4F/GFP only RPA UT.png]

SH-SY5Y

EV sgMAEA

190

115

80

50

30

25

15

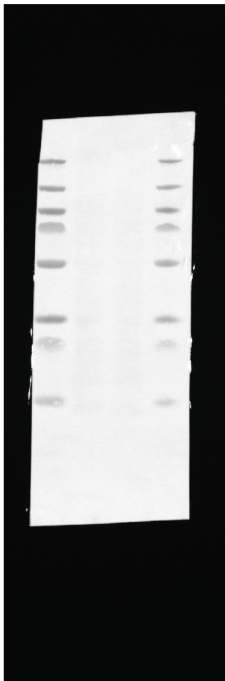

Supplement: Supplementary file 19 — Figure EV4 Source Data [file 44321_2025_352_MOESM19_ESM.zip › EMM-2025-21907-V2_SourceDataFigEV4/EV4A/EV4A_ladders.pdf]

SH-SY5Y

EV sgMAEA

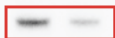

MAEA

Supplement: Supplementary file 19 — Figure EV4 Source Data [file 44321_2025_352_MOESM19_ESM.zip › EMM-2025-21907-V2_SourceDataFigEV4/EV4A/EV4A_maea.pdf]

SH-SY5Y

EV sgMAEA

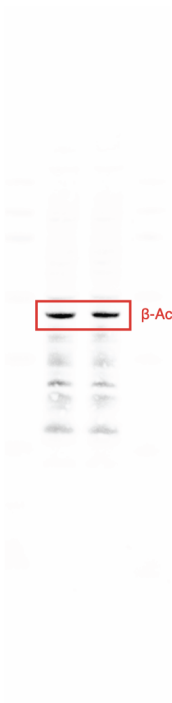

Supplement: Supplementary file 19 — Figure EV4 Source Data [file 44321_2025_352_MOESM19_ESM.zip › EMM-2025-21907-V2_SourceDataFigEV4/EV4A/EV4A_actin.pdf]

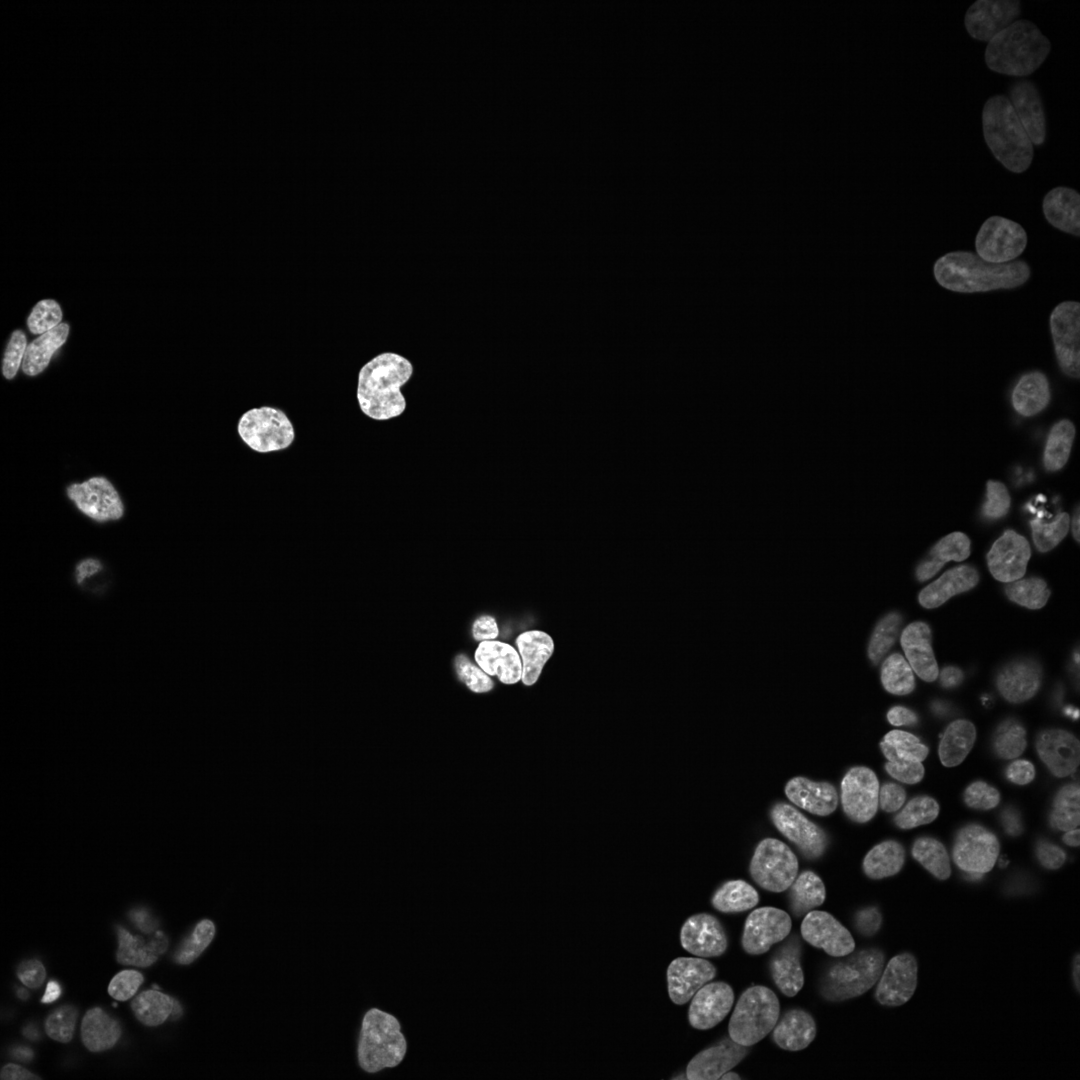

Supplement: Supplementary file 19 — Figure EV4 Source Data [file 44321_2025_352_MOESM19_ESM.zip › EMM-2025-21907-V2_SourceDataFigEV4/EV4C/M_CPT_BrdU_DAPI_.tif]

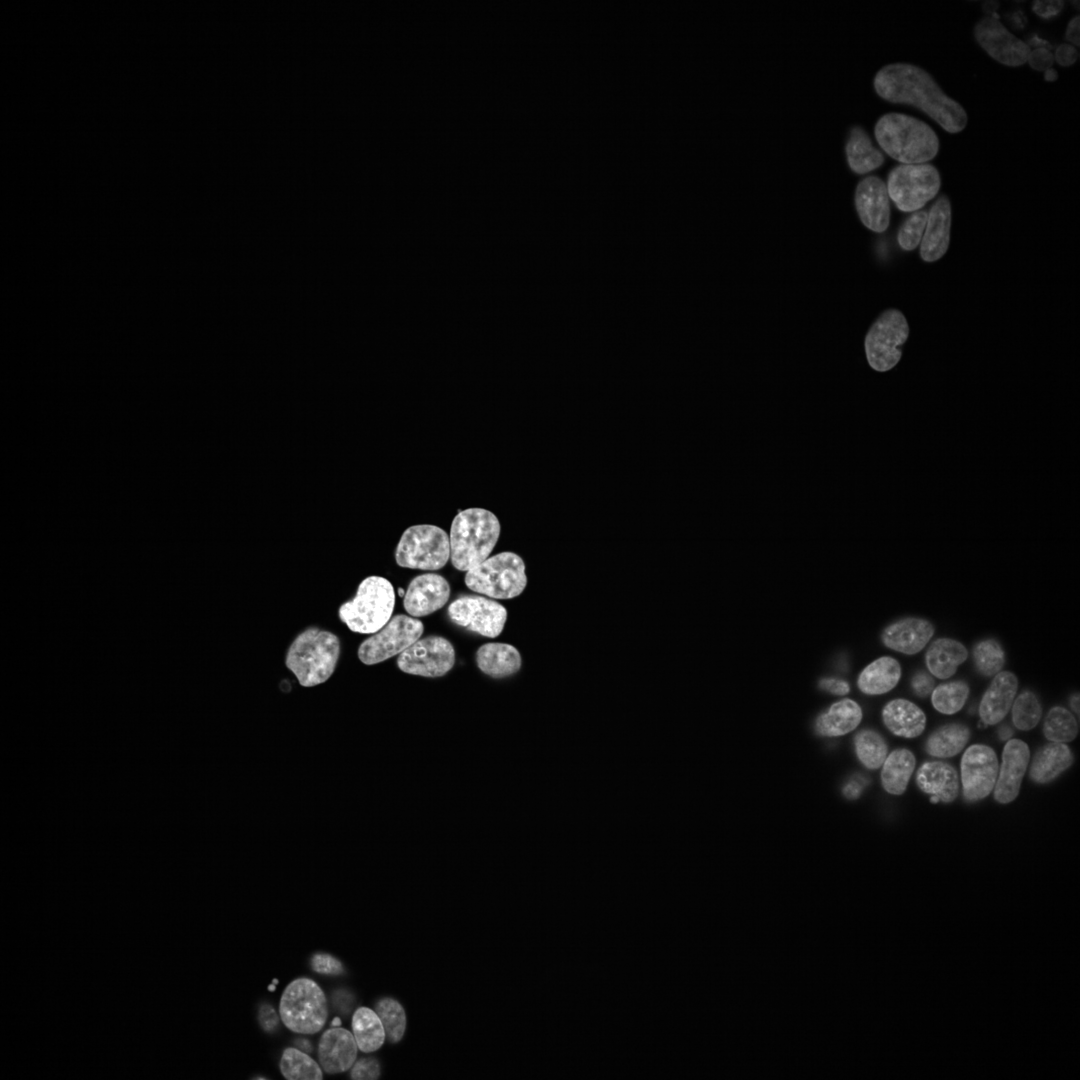

Supplement: Supplementary file 19 — Figure EV4 Source Data [file 44321_2025_352_MOESM19_ESM.zip › EMM-2025-21907-V2_SourceDataFigEV4/EV4C/WT_DMSO_BrdU_DAPI_.tif]

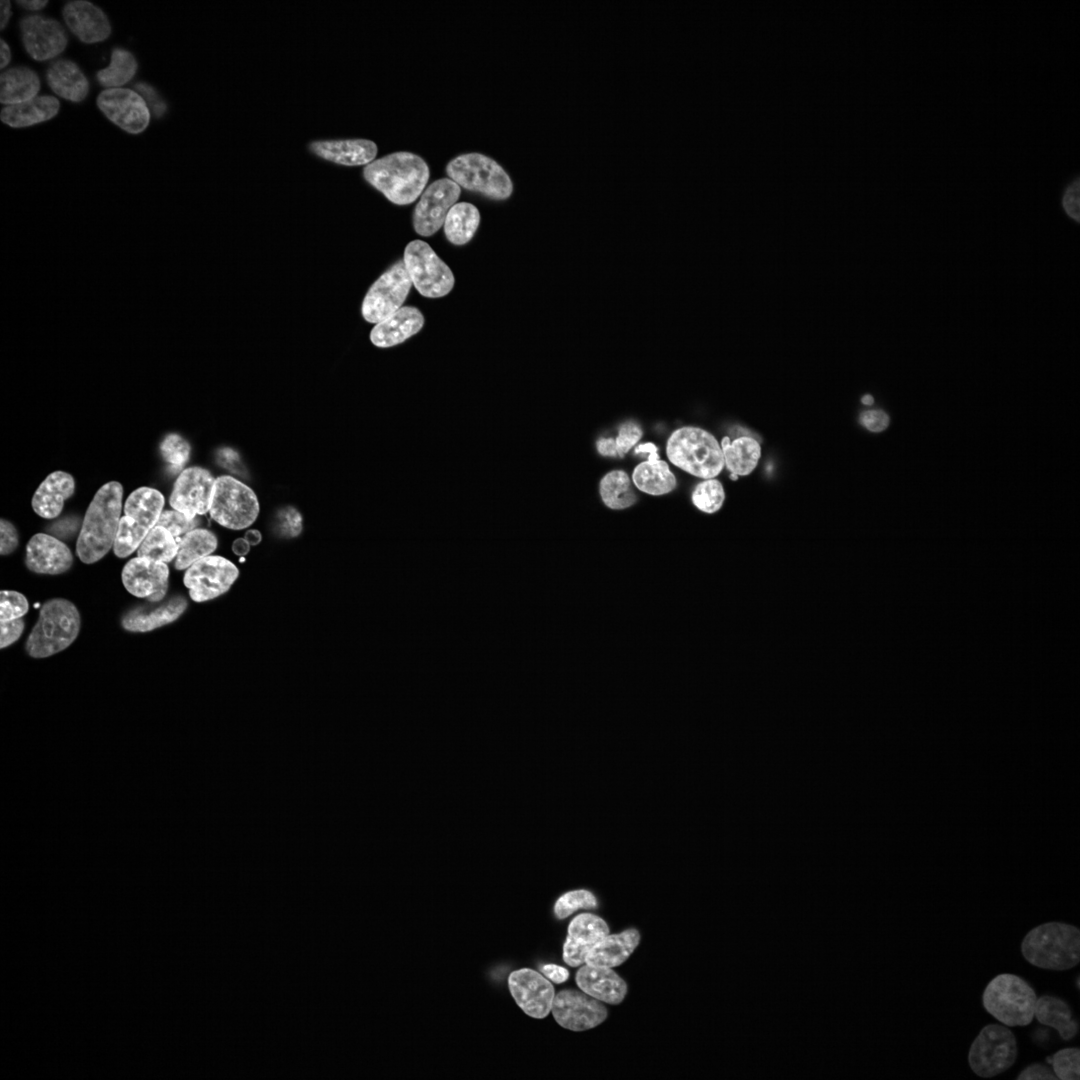

Supplement: Supplementary file 19 — Figure EV4 Source Data [file 44321_2025_352_MOESM19_ESM.zip › EMM-2025-21907-V2_SourceDataFigEV4/EV4C/M_DMSO_BrdU_DAPI_.tif]

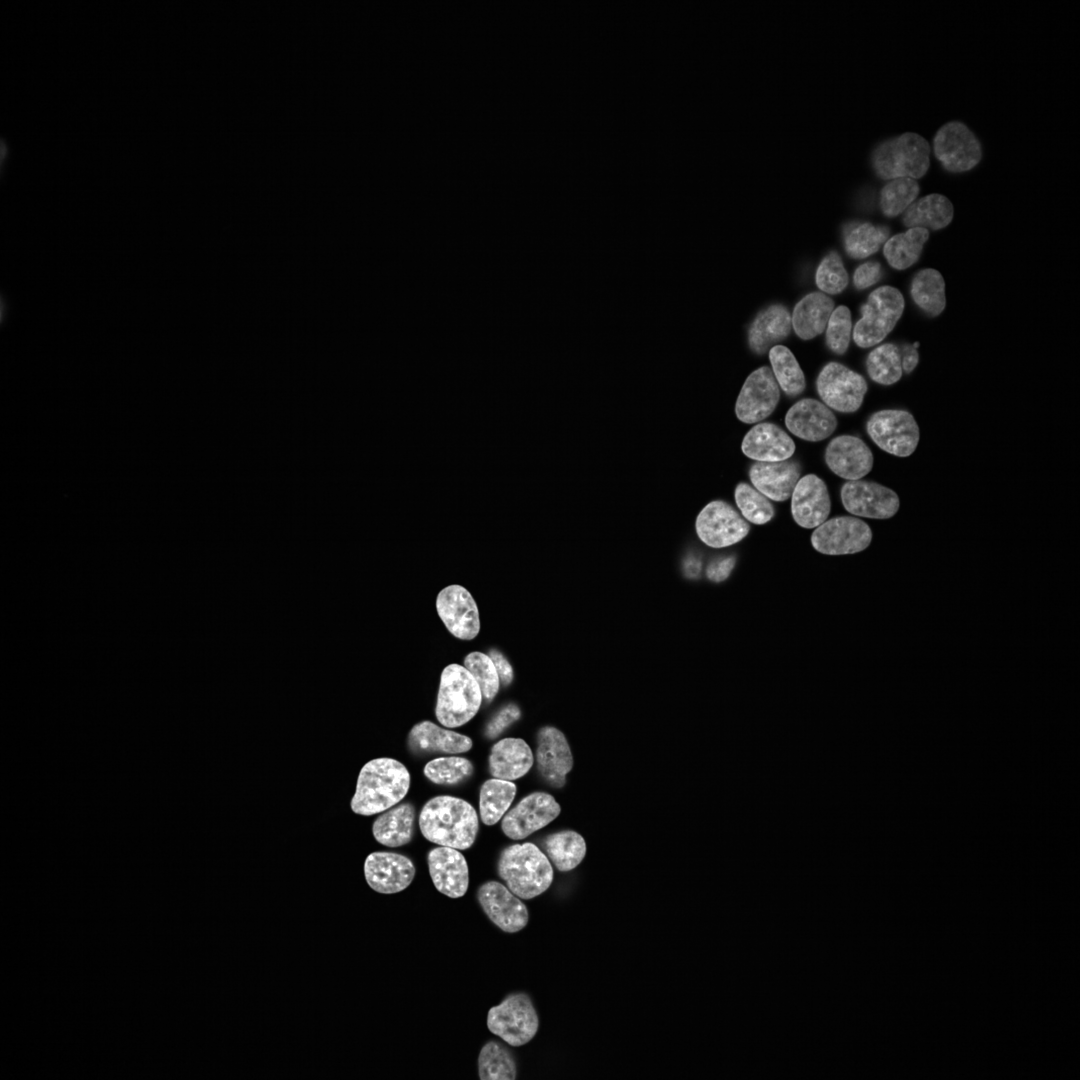

Supplement: Supplementary file 19 — Figure EV4 Source Data [file 44321_2025_352_MOESM19_ESM.zip › EMM-2025-21907-V2_SourceDataFigEV4/EV4C/WT_CPT_BrdU_DAPI_.tif]

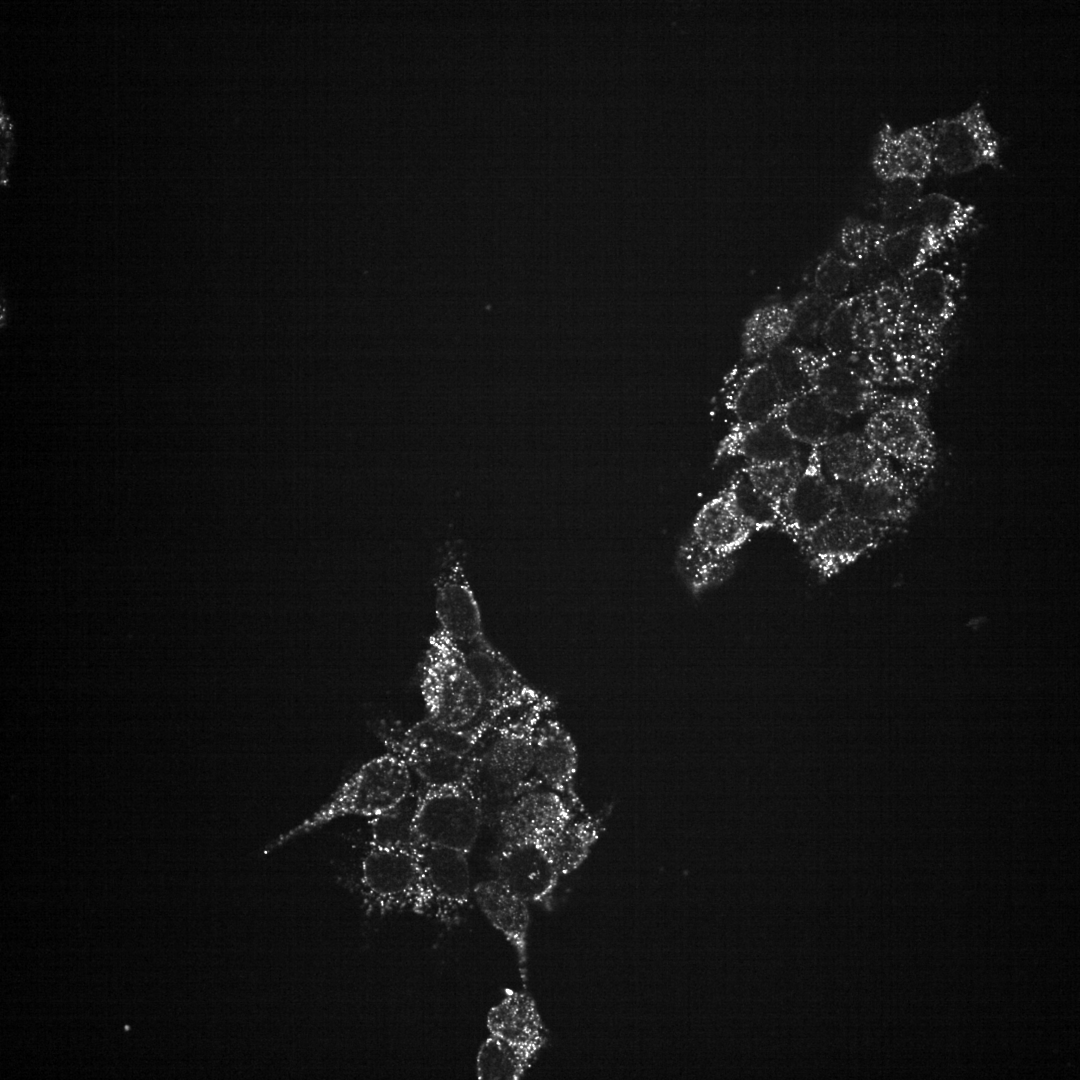

Supplement: Supplementary file 19 — Figure EV4 Source Data [file 44321_2025_352_MOESM19_ESM.zip › EMM-2025-21907-V2_SourceDataFigEV4/EV4C/WT_CPT_BrdU_Alexa 488_.tif]

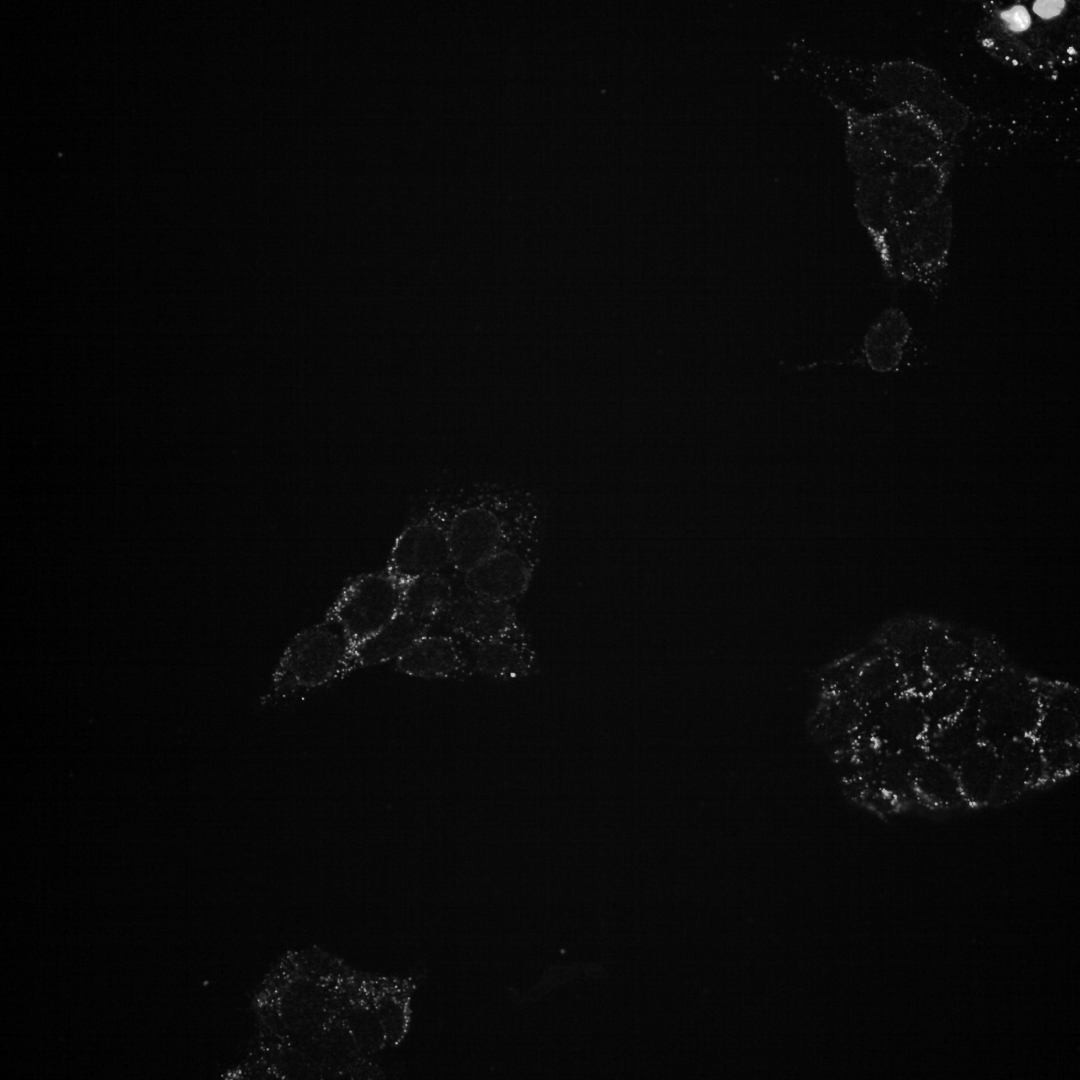

Supplement: Supplementary file 19 — Figure EV4 Source Data [file 44321_2025_352_MOESM19_ESM.zip › EMM-2025-21907-V2_SourceDataFigEV4/EV4C/WT_DMSO_BrdU_Alexa 488_.tif]

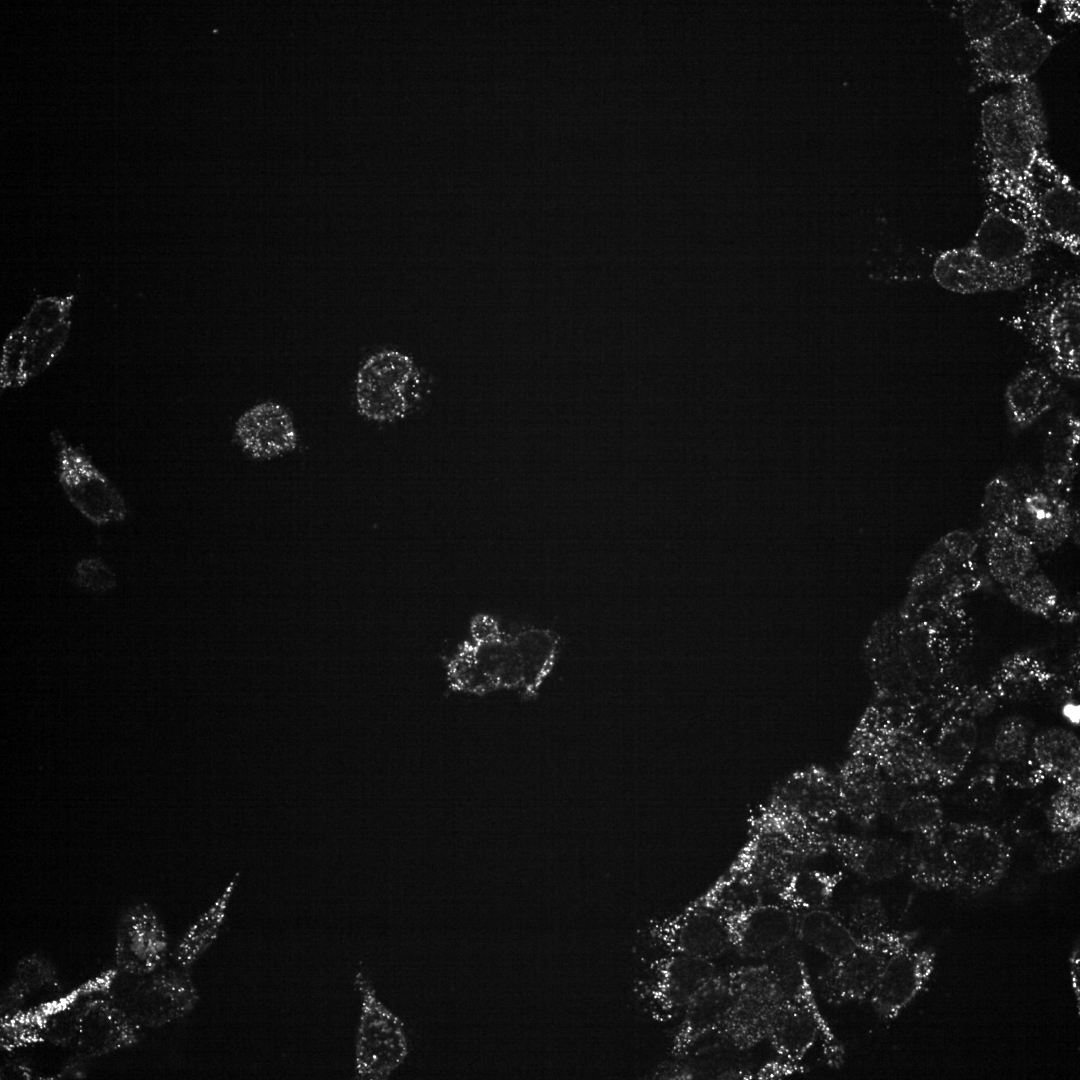

Supplement: Supplementary file 19 — Figure EV4 Source Data [file 44321_2025_352_MOESM19_ESM.zip › EMM-2025-21907-V2_SourceDataFigEV4/EV4C/M_CPT_BrdU_Alexa 488_.tif]

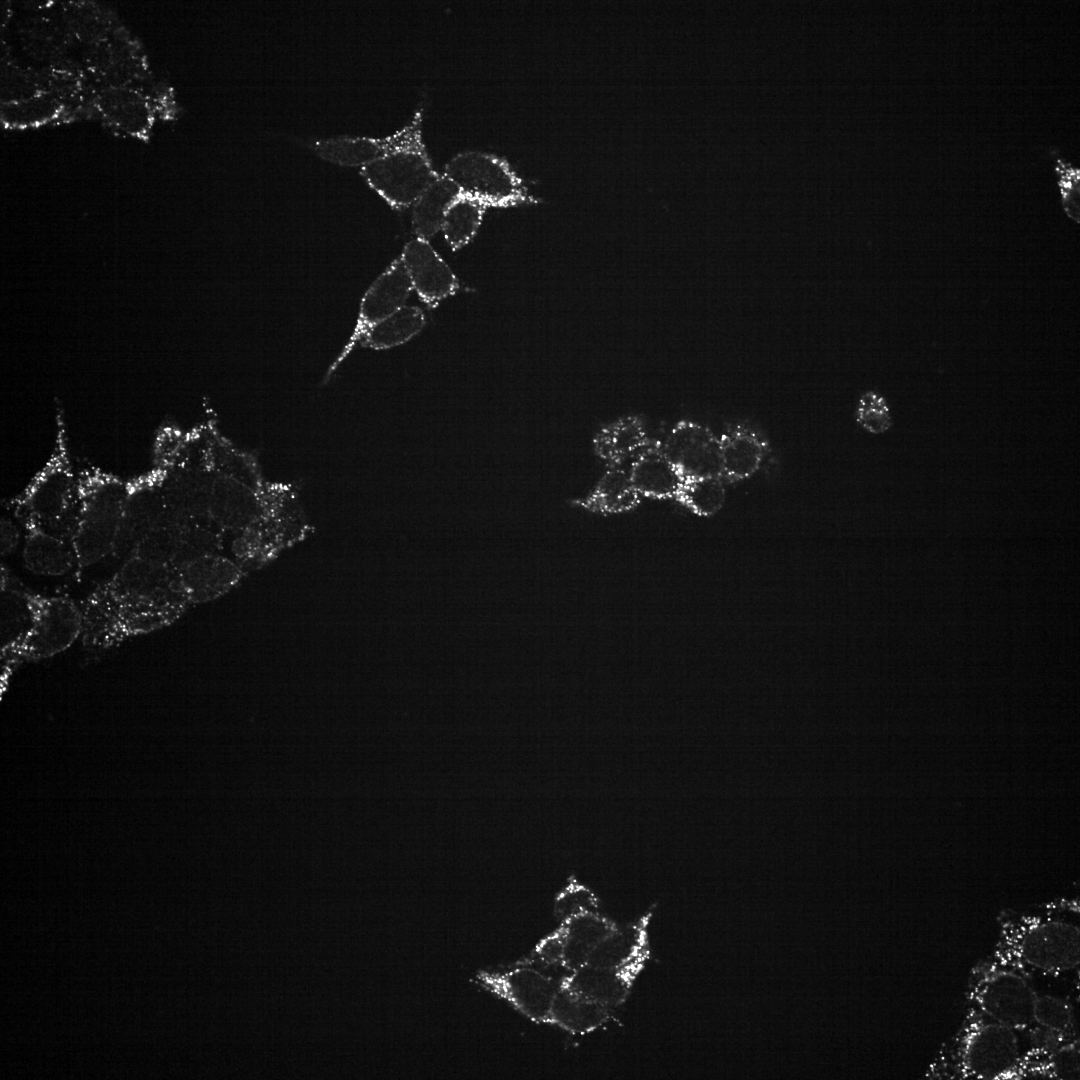

Supplement: Supplementary file 19 — Figure EV4 Source Data [file 44321_2025_352_MOESM19_ESM.zip › EMM-2025-21907-V2_SourceDataFigEV4/EV4C/M_DMSO_BrdU_Alexa 488_.tif]

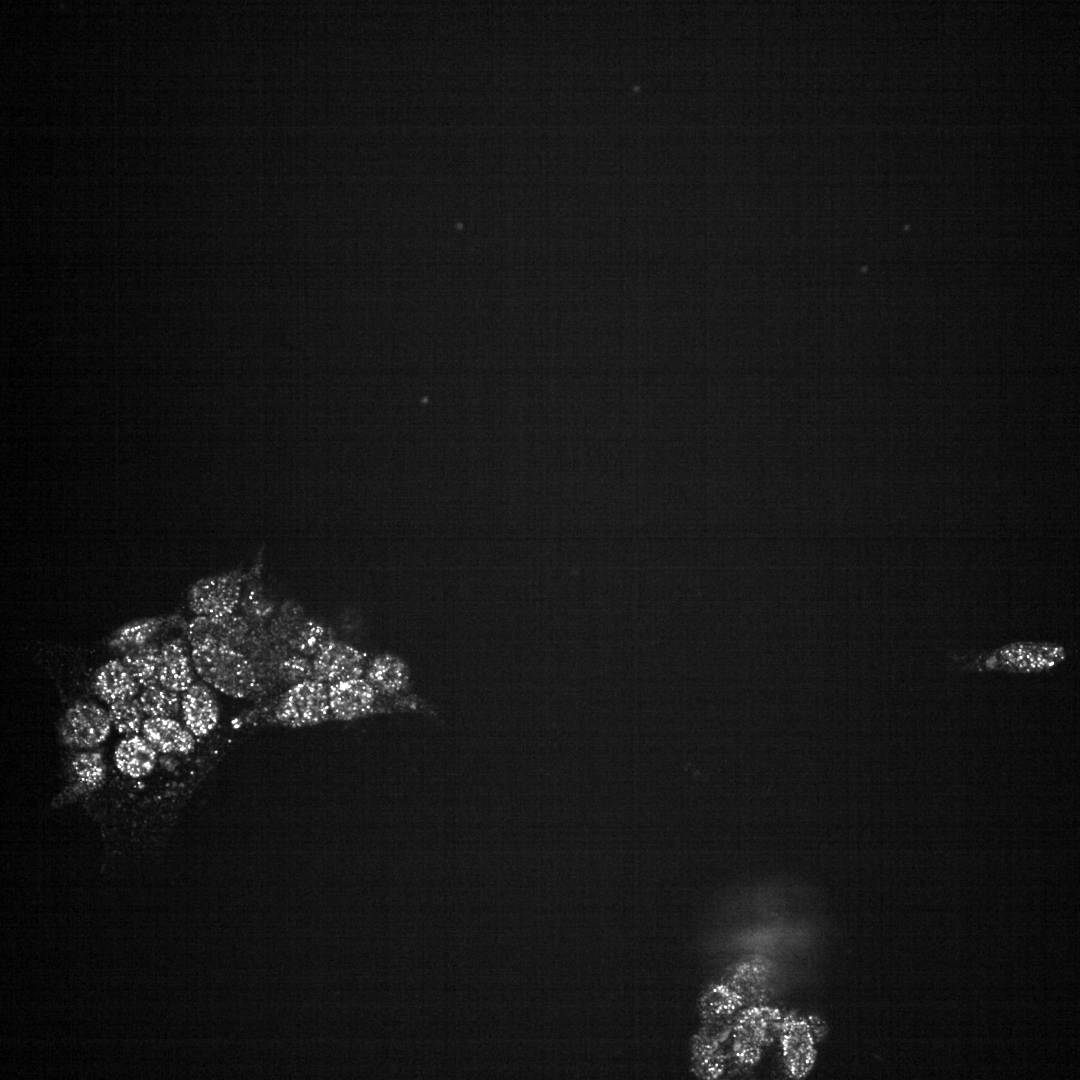

Supplement: Supplementary file 19 — Figure EV4 Source Data [file 44321_2025_352_MOESM19_ESM.zip › EMM-2025-21907-V2_SourceDataFigEV4/EV4E/WT_CPT_RAD51_Alexa 488_.tif]

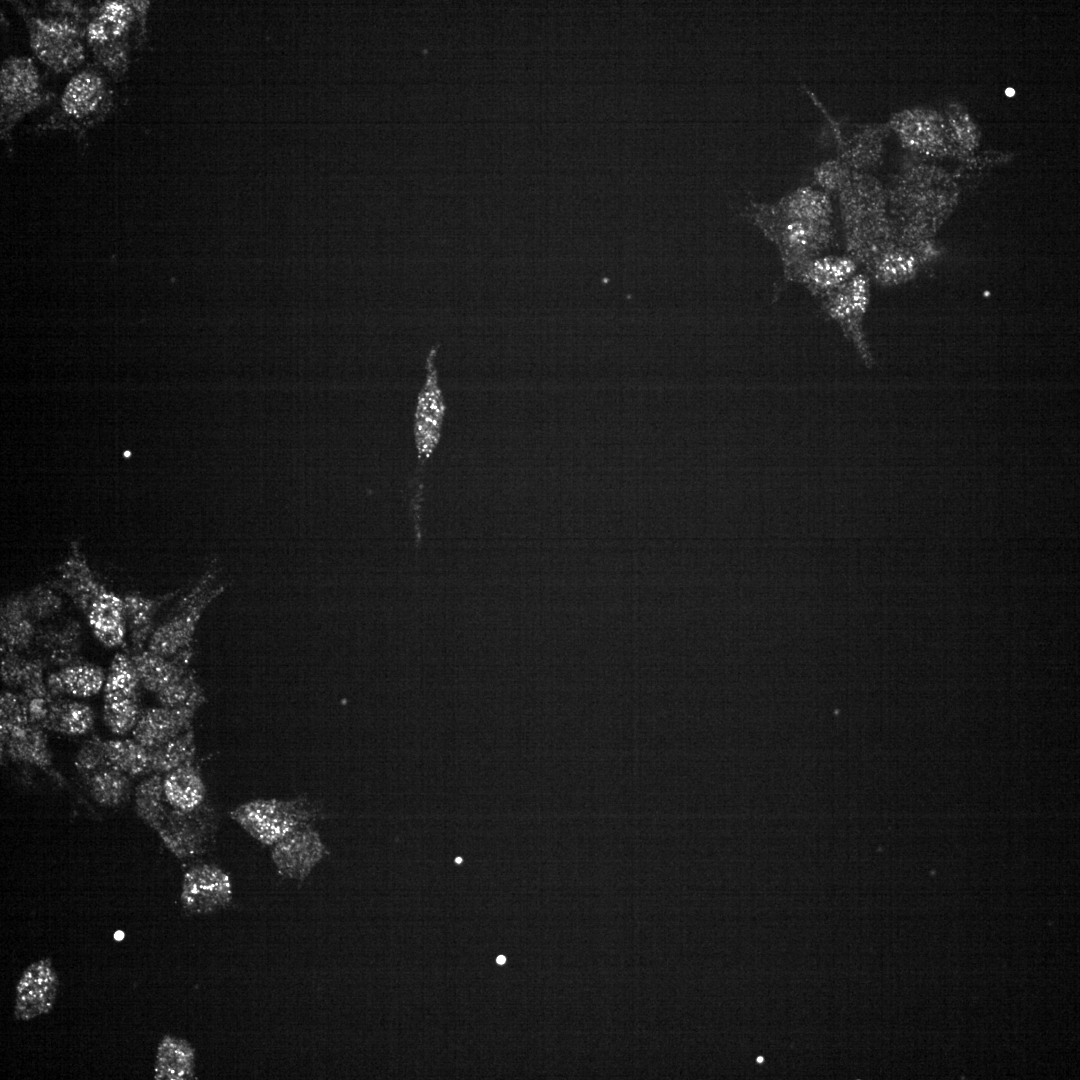

Supplement: Supplementary file 19 — Figure EV4 Source Data [file 44321_2025_352_MOESM19_ESM.zip › EMM-2025-21907-V2_SourceDataFigEV4/EV4E/M_CPT_RAD51_Alexa 488_.tif]

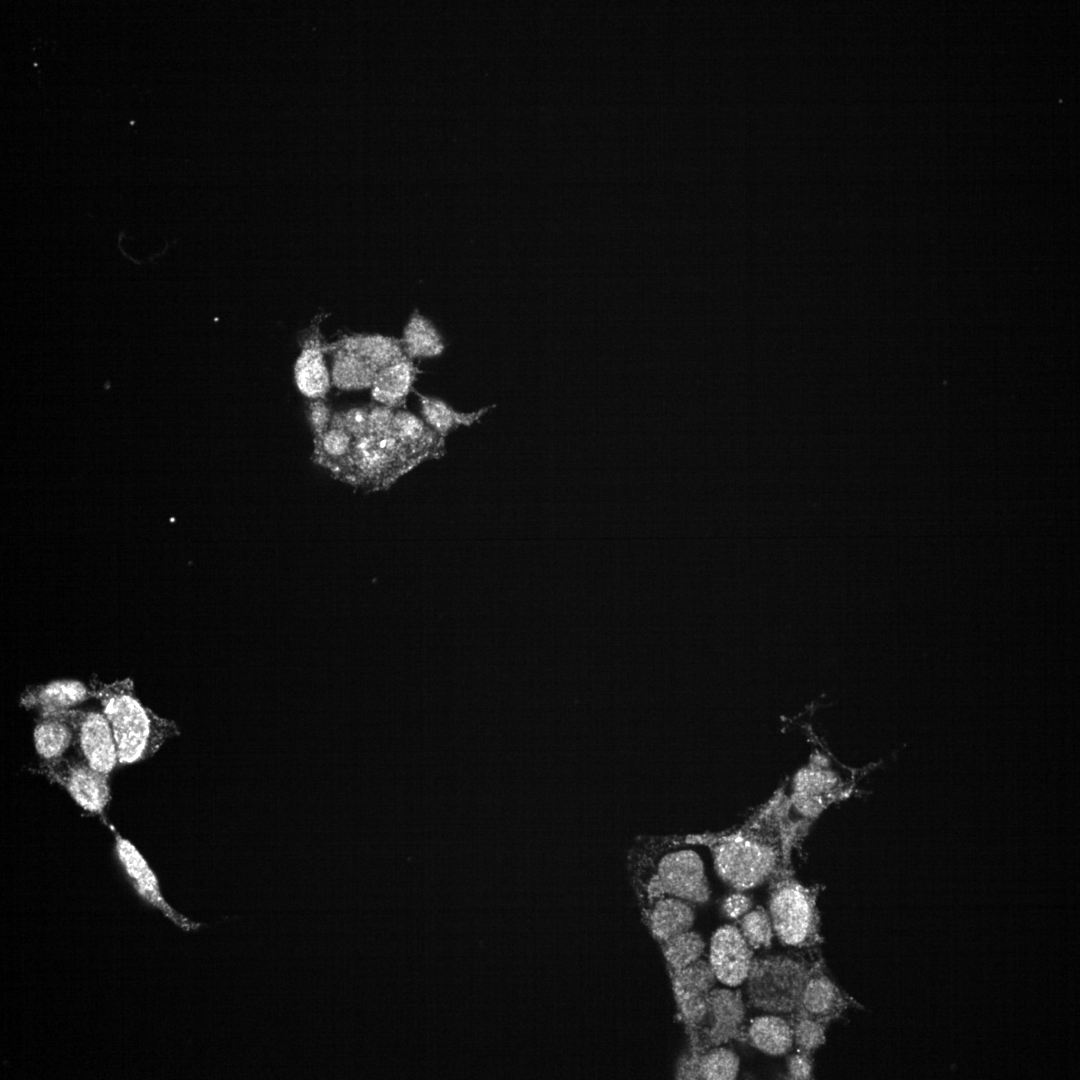

Supplement: Supplementary file 19 — Figure EV4 Source Data [file 44321_2025_352_MOESM19_ESM.zip › EMM-2025-21907-V2_SourceDataFigEV4/EV4E/WT_DMSO_RAD51_Alexa 568_.tif]

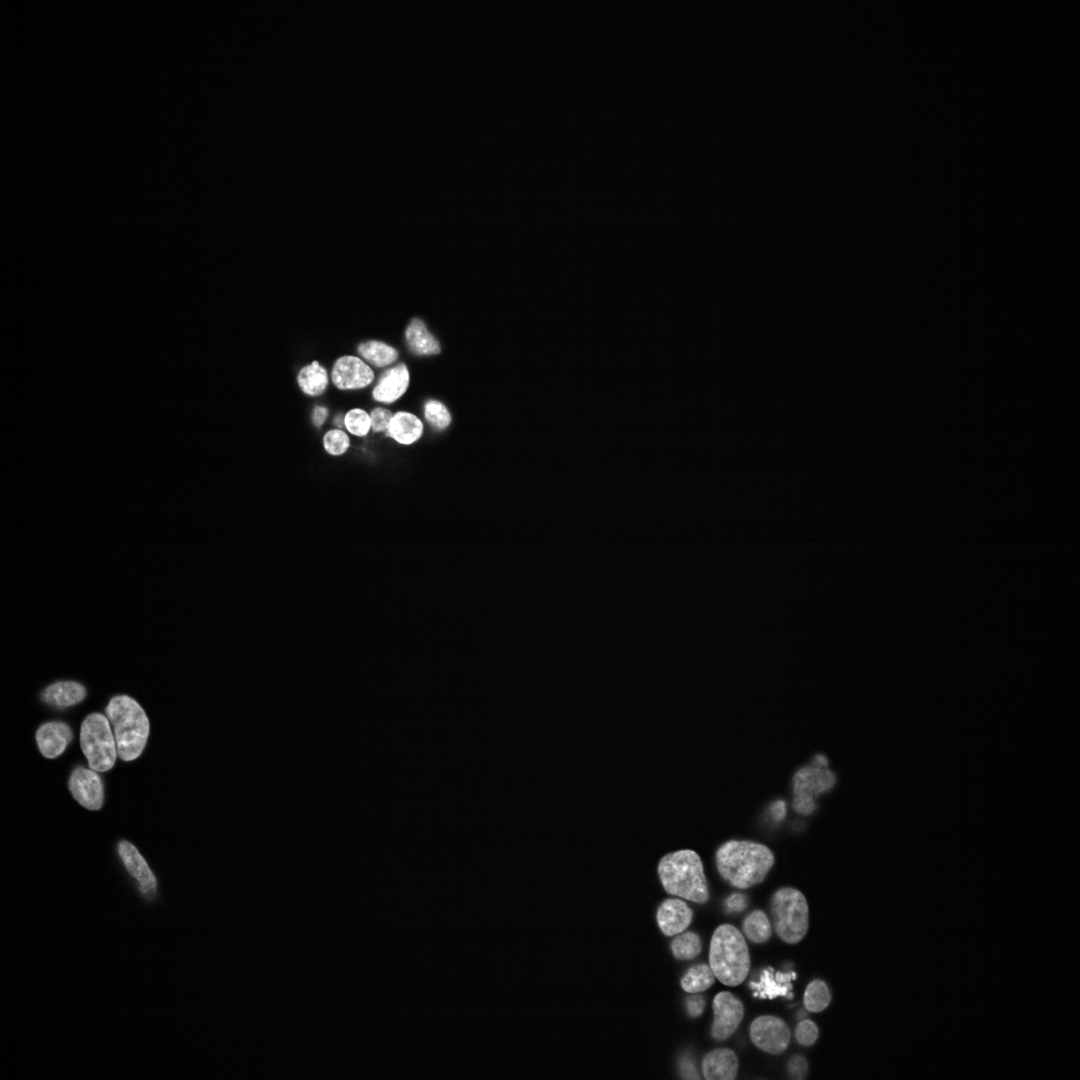

Supplement: Supplementary file 19 — Figure EV4 Source Data [file 44321_2025_352_MOESM19_ESM.zip › EMM-2025-21907-V2_SourceDataFigEV4/EV4E/WT_DMSO_RAD51_DAPI_.tif]

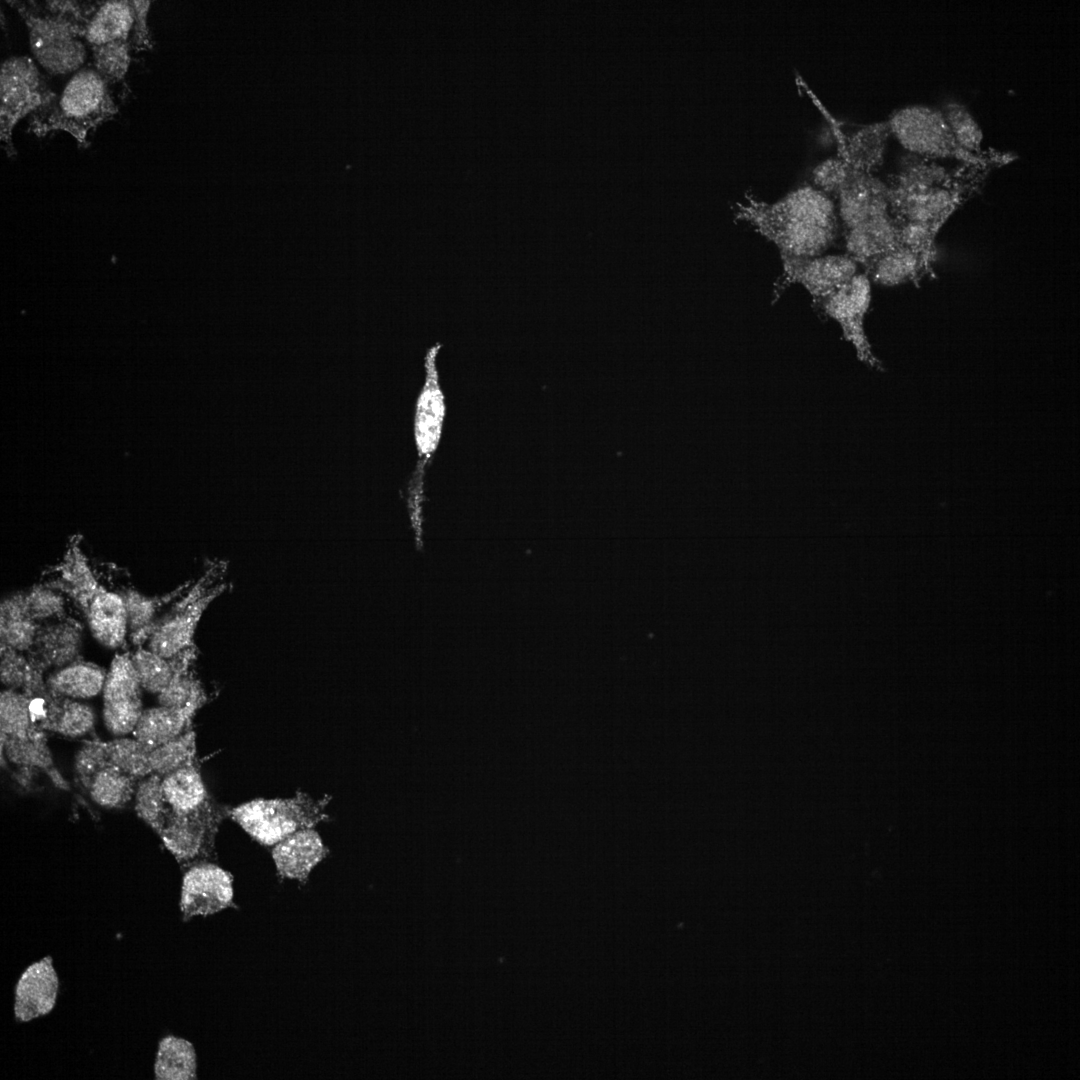

Supplement: Supplementary file 19 — Figure EV4 Source Data [file 44321_2025_352_MOESM19_ESM.zip › EMM-2025-21907-V2_SourceDataFigEV4/EV4E/M_CPT_RAD51_Alexa 568_.tif]

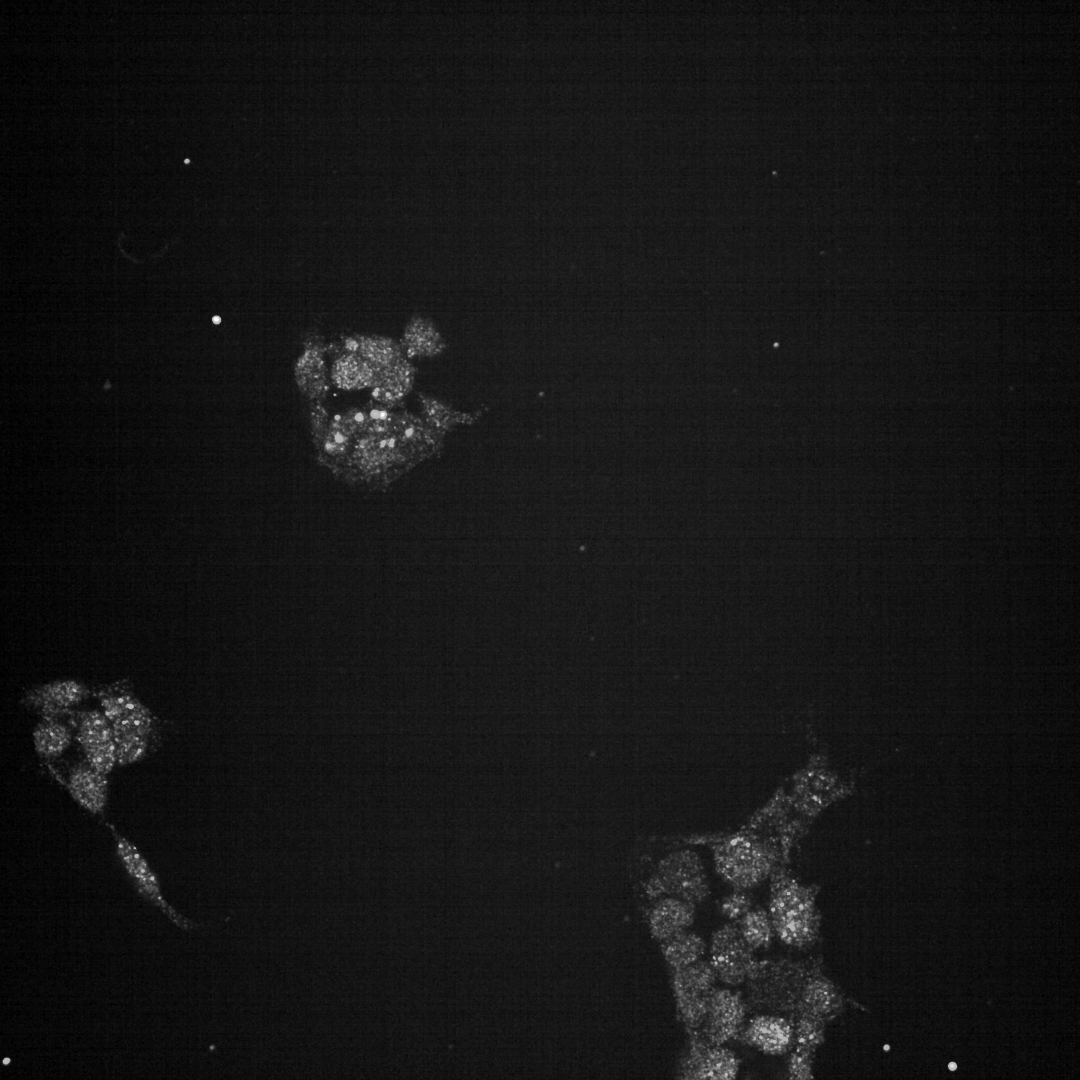

Supplement: Supplementary file 19 — Figure EV4 Source Data [file 44321_2025_352_MOESM19_ESM.zip › EMM-2025-21907-V2_SourceDataFigEV4/EV4E/WT_DMSO_RAD51_Alexa 488_.tif]

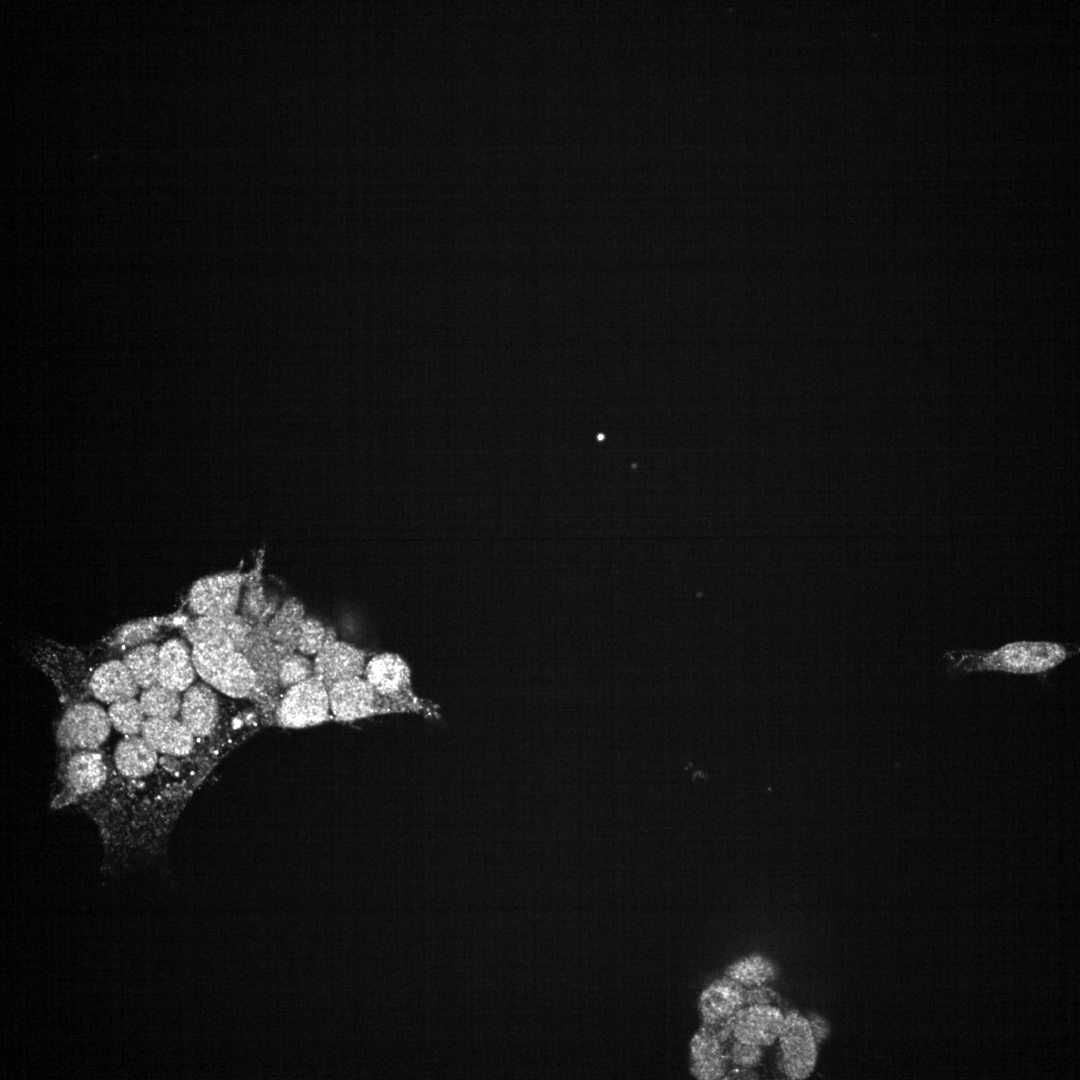

Supplement: Supplementary file 19 — Figure EV4 Source Data [file 44321_2025_352_MOESM19_ESM.zip › EMM-2025-21907-V2_SourceDataFigEV4/EV4E/WT_CPT_RAD51_Alexa 568_.tif]

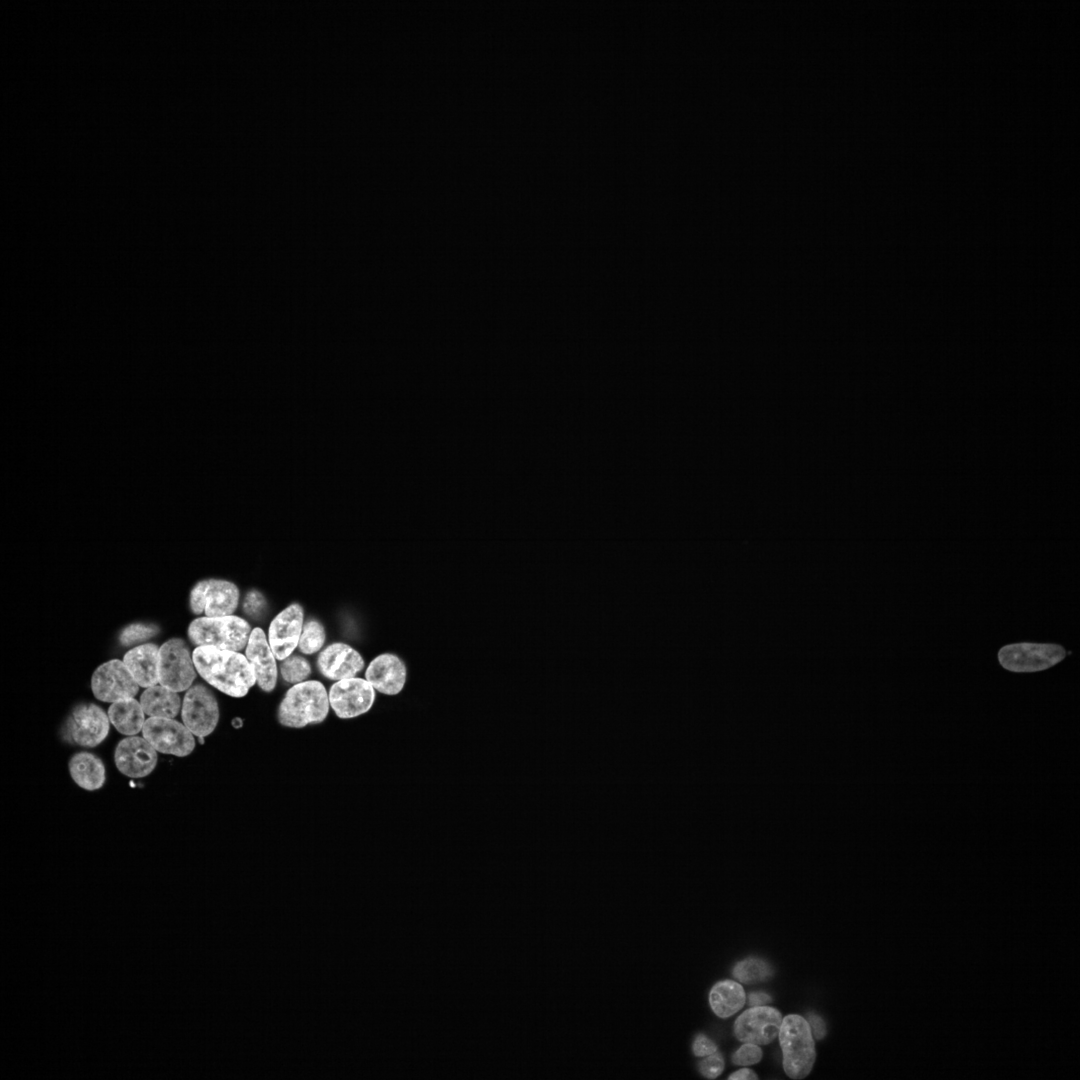

Supplement: Supplementary file 19 — Figure EV4 Source Data [file 44321_2025_352_MOESM19_ESM.zip › EMM-2025-21907-V2_SourceDataFigEV4/EV4E/WT_CPT_RAD51_DAPI_.tif]

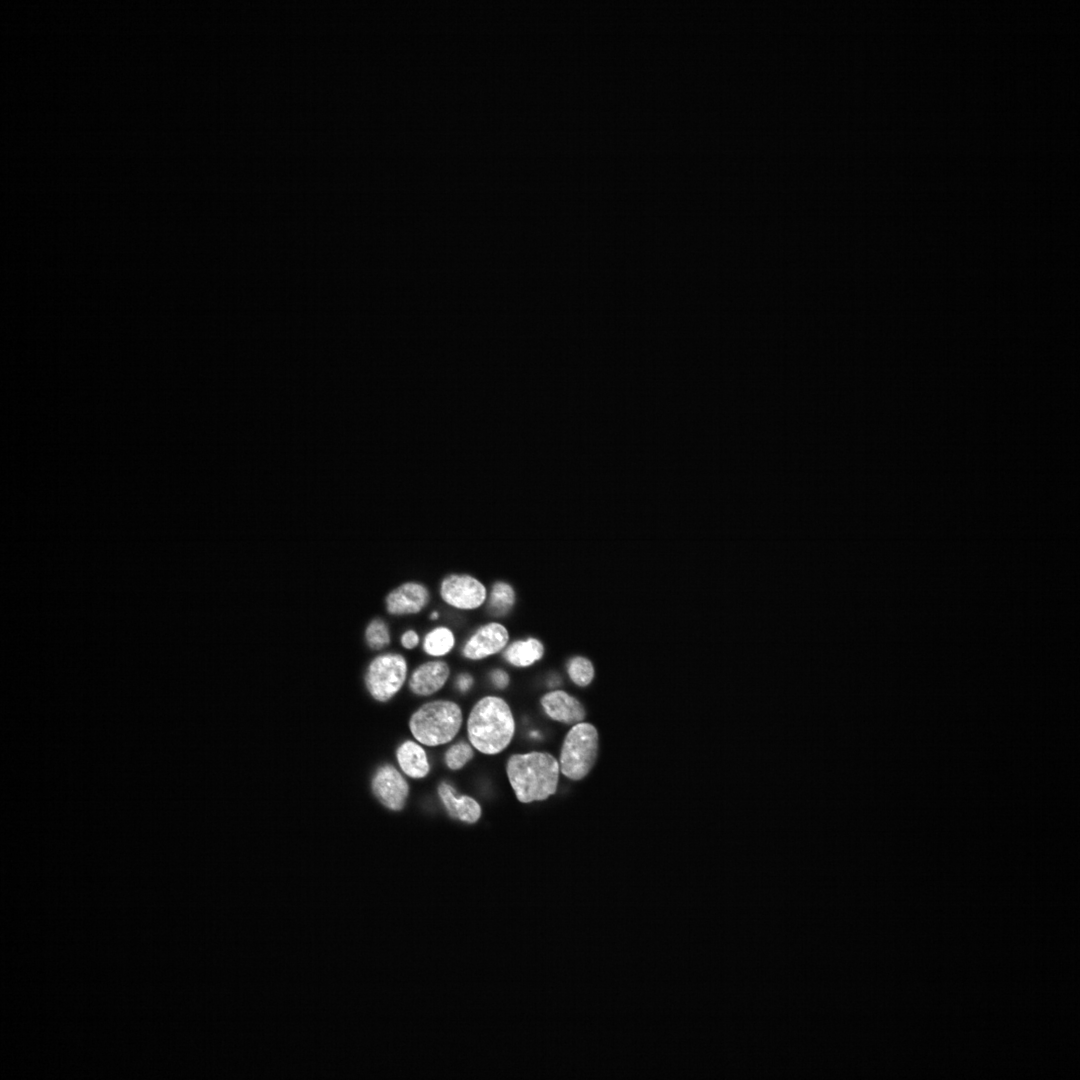

Supplement: Supplementary file 19 — Figure EV4 Source Data [file 44321_2025_352_MOESM19_ESM.zip › EMM-2025-21907-V2_SourceDataFigEV4/EV4E/M_DMSO_RAD51_DAPI_.tif]

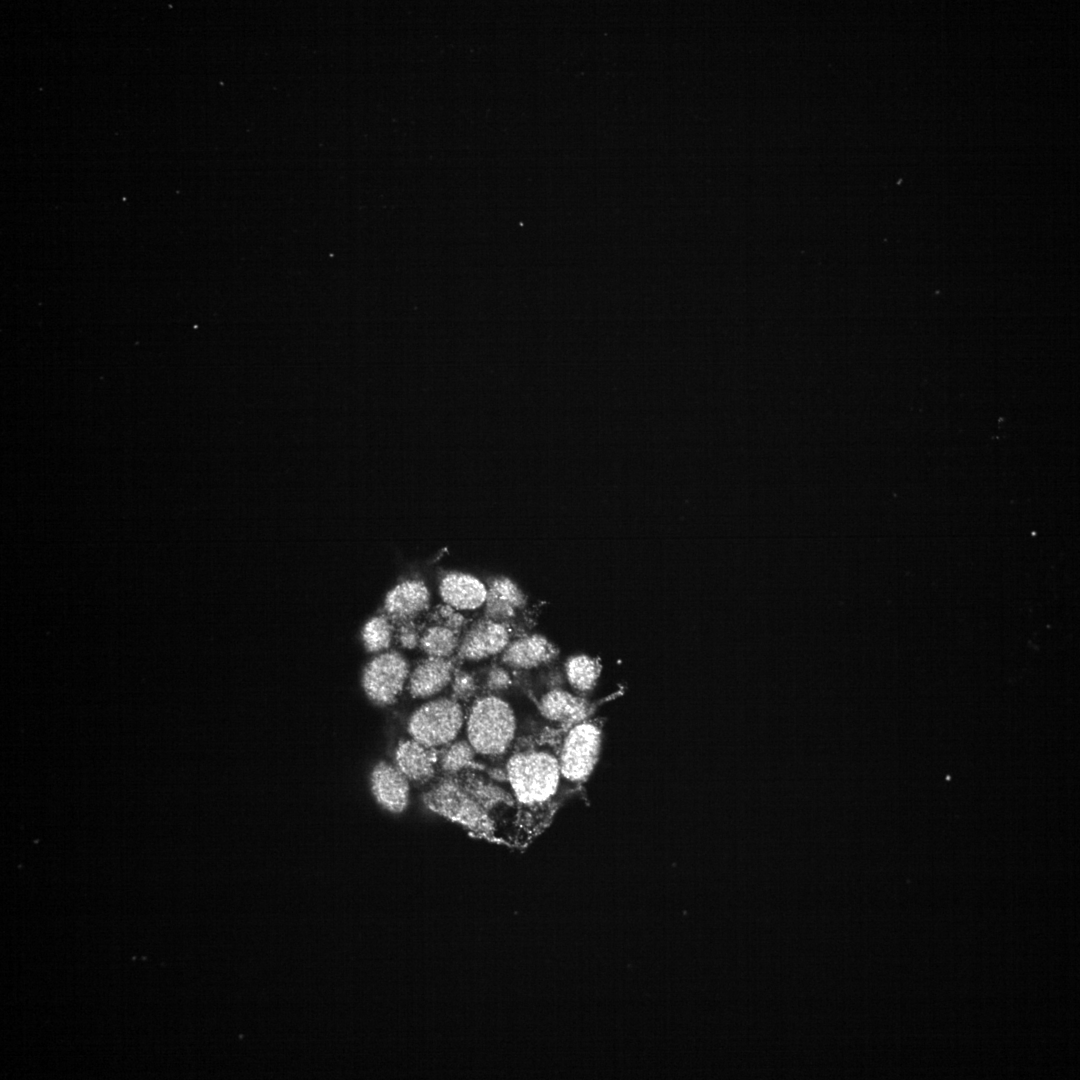

Supplement: Supplementary file 19 — Figure EV4 Source Data [file 44321_2025_352_MOESM19_ESM.zip › EMM-2025-21907-V2_SourceDataFigEV4/EV4E/M_DMSO_RAD51_Alexa 568_.tif]

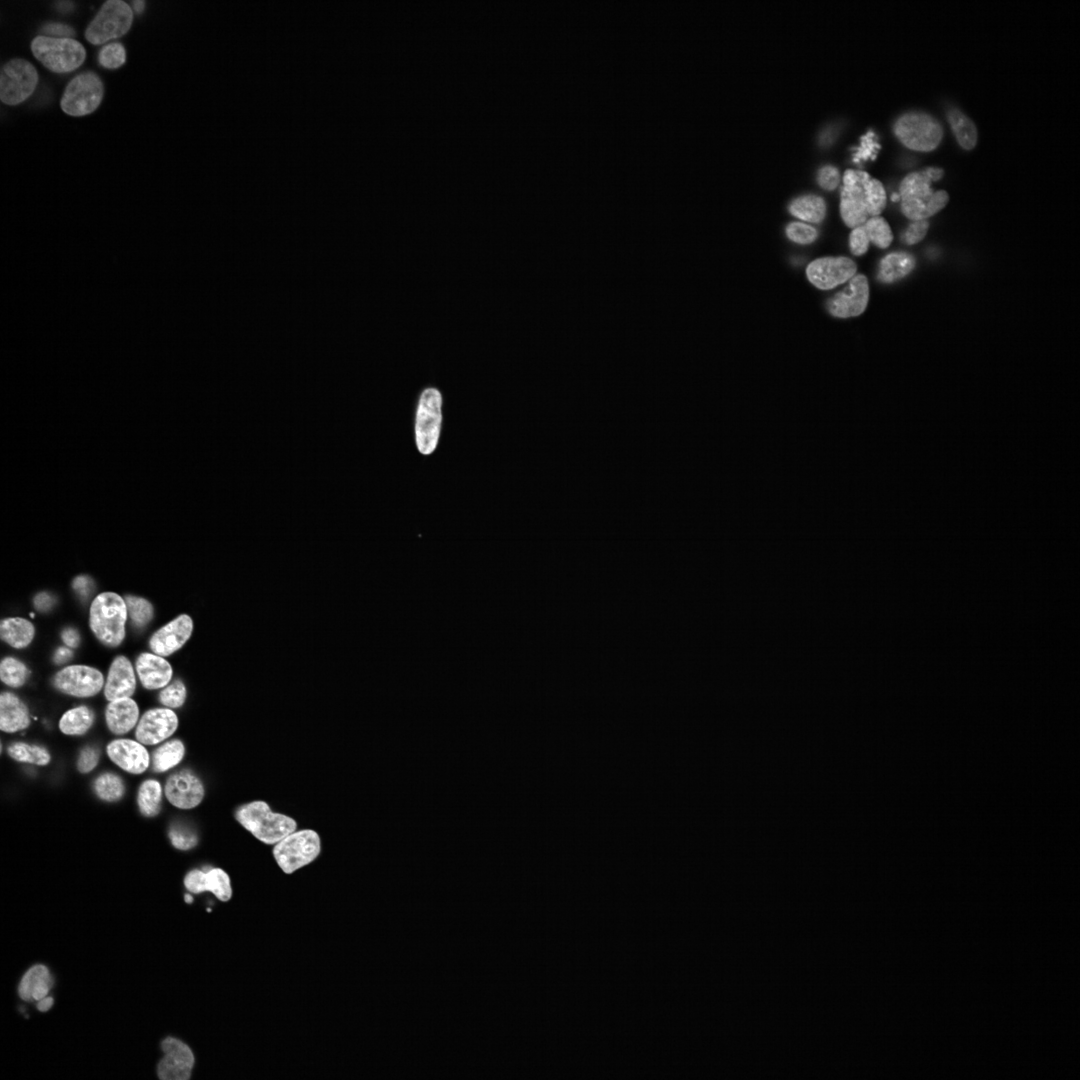

Supplement: Supplementary file 19 — Figure EV4 Source Data [file 44321_2025_352_MOESM19_ESM.zip › EMM-2025-21907-V2_SourceDataFigEV4/EV4E/M_CPT_RAD51_DAPI_.tif]

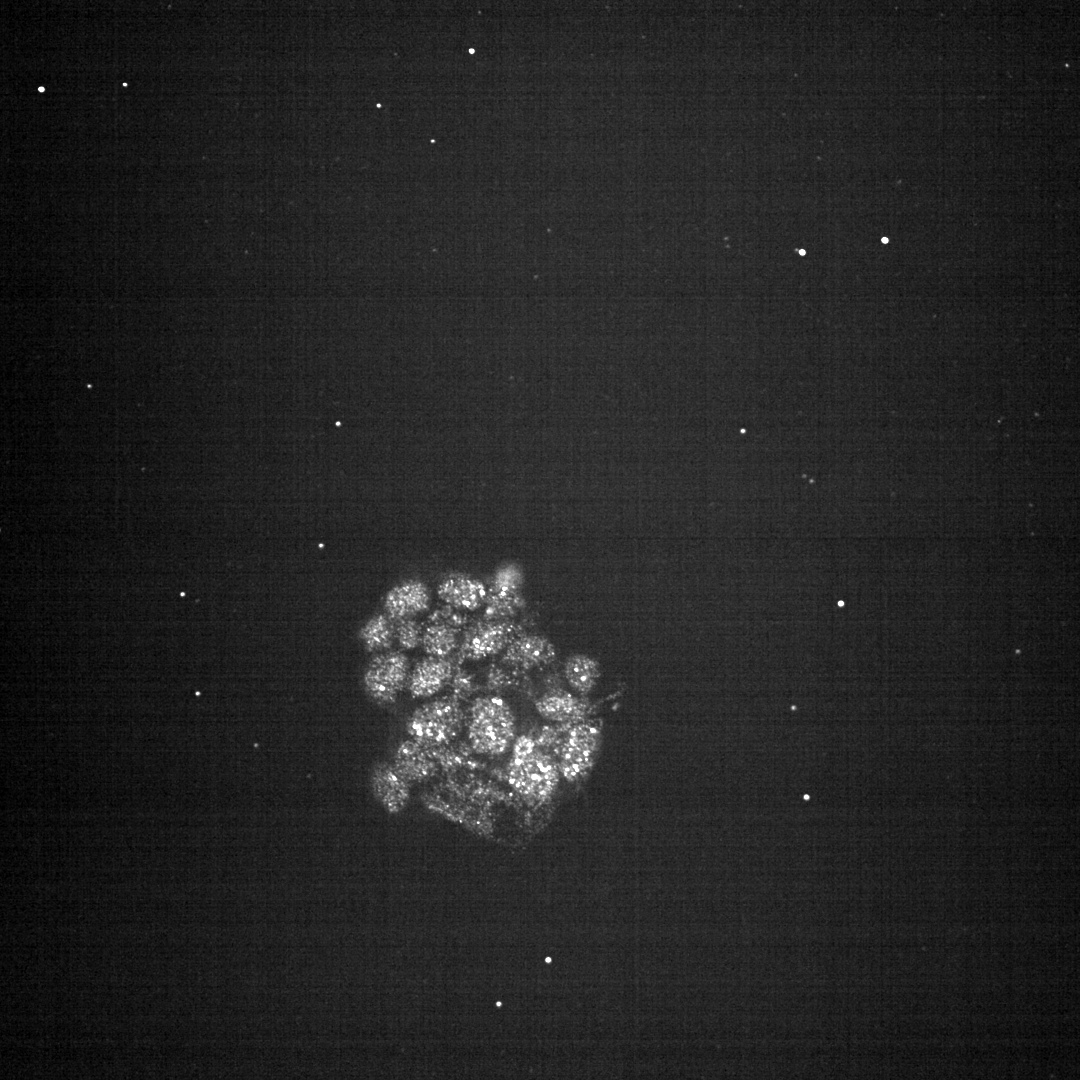

Supplement: Supplementary file 19 — Figure EV4 Source Data [file 44321_2025_352_MOESM19_ESM.zip › EMM-2025-21907-V2_SourceDataFigEV4/EV4E/M_DMSO_RAD51_Alexa 488_.tif]
