# Supplementary material for: Loss of CTLH component MAEA impairs DNA repair and replication and leads to developmental delay
Source: EMBO Mol Med. 2025 Dec 19;18(2):492–513. doi: 10.1038/s44321-025-00352-x (PMC12905269; doi:10.1038/s44321-025-00352-x)
Supplement: Supplementary file 20 — Expanded View Figures [file 44321_2025_352_MOESM20_ESM.pdf]

Expanded View Figures

Figure EV1. *MAEA* KO and HM cell line validation.

(**A**) Immunoblot for MAEA in the indicated U2OS cell lines; $n = 2$ independent experiments. (**B**) TIDE analysis of the sgRNA target site in *MAEA* KO and *MAEA* HM cells. (**C**) Sanger trace analysis of the sgRNA target site in *MAEA* KO and HM cells. (**D**) Cell cycle analysis of *MAEA* KO and HM cells. (**E**, **F**) Representative images of untreated WT and MAEA-deficient U2OS (**E**) and HAP1 (**F**) cells. (**G**) Immunoblot for MAEA in U2OS following siRNA transfection; $n = 3$ independent experiments. (**H**, **I**) Representative images (**H**) and colony counts (**I**) of U2OS cells with or without siRNA-mediated MAEA depletion. (**J**) The average colony size of siRNA-transfected U2OS cells at 500 cells per well. (**K**) Total area coverage of U2OS colonies following siRNA transfection. (**I–K**) Bars represent the mean ± SEM. KO knockout, HM hypomorph.
Source data are available online for this figure.

© The Author(s)

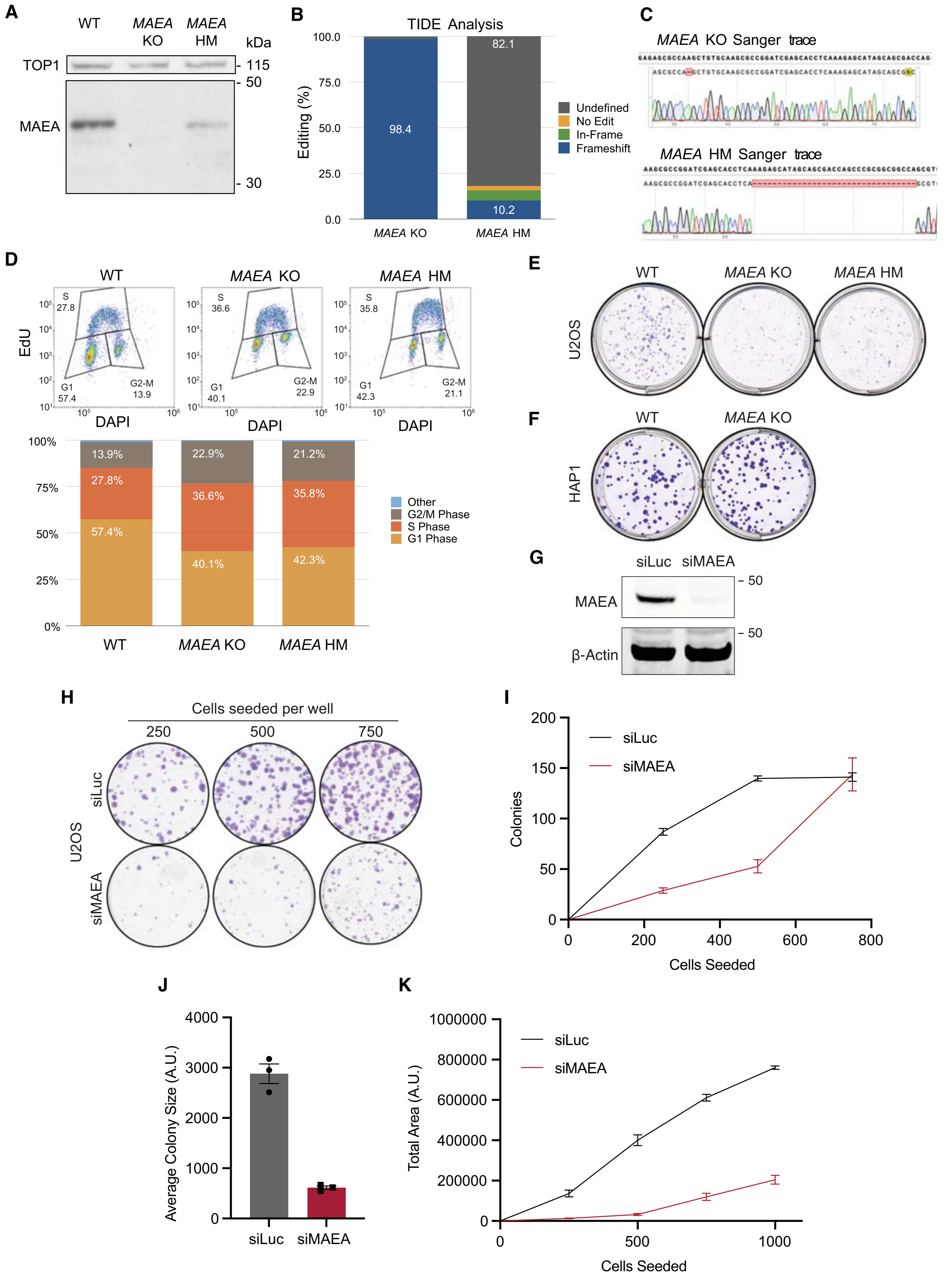
A
WT
MAEA KO
MAEA HM
kDa
TOP1
115
50
MAEA
30
B
TIDE Analysis
Editing (%)
100.0
75.0
50.0
25.0
0.0
98.4
82.1
10.2
MAEA KO
MAEA HM
Undefined
No Edit
In-Frame
Frameshift
C
MAEA KO Sanger trace
MAEA HM Sanger trace
D
WT
MAEA KO
MAEA HM
EdU
DAPI
S 27.8
G1 57.4
G2-M 13.9
S 36.6
G1 40.1
G2-M 22.9
S 35.8
G1 42.3
G2-M 21.1
100%
75%
50%
25%
0%
13.9%
27.8%
57.4%
22.9%
36.6%
40.1%
21.2%
35.8%
42.3%
Other
G2/M Phase
S Phase
G1 Phase
E
WT
MAEA KO
MAEA HM
U2OS
F
WT
MAEA KO
HAP1
G
siLuc
siMAEA
MAEA
β-Actin
50
50
H
Cells seeded per well
250
500
750
siLuc
siMAEA
U2OS
I
siLuc
siMAEA
Colonies
Cells Seeded
200
150
100
50
0
0
200
400
600
800
J
Average Colony Size (A.U.)
4000
3000
2000
1000
0
siLuc
siMAEA
K
siLuc
siMAEA
Total Area (A.U.)
1000000
800000
600000
400000
200000
0
0
500
1000
Cells Seeded

© The Author(s)

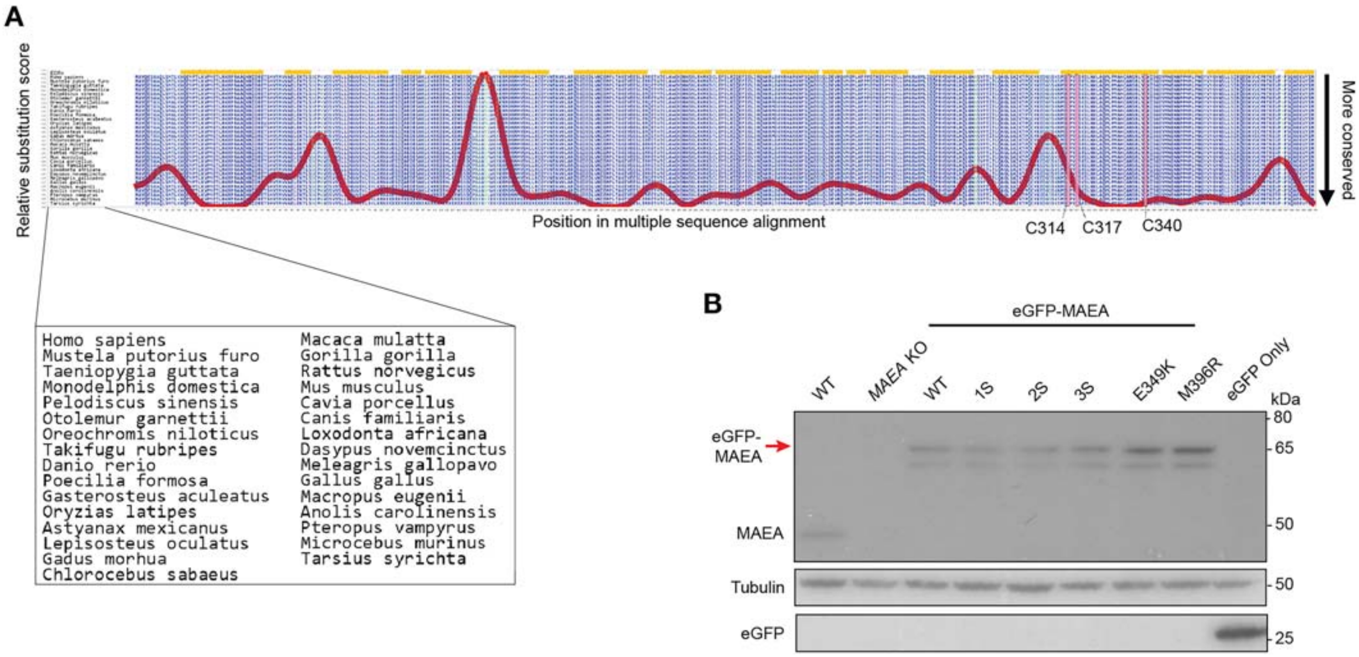

Figure EV2. Clinical and C > S MAEA variants are evolutionarily conserved.

(**A**) Aminode analysis (Chang et al, 2018) of MAEA, with C > S mutations annotated. The red line represents conservation across species. Species compared in the analysis are listed in the box. (**B**) Immunoblot assessing expression of eGFP-MAEA constructs; $n = 2$ independent experiments. Source data are available online for this figure.

© The Author(s)

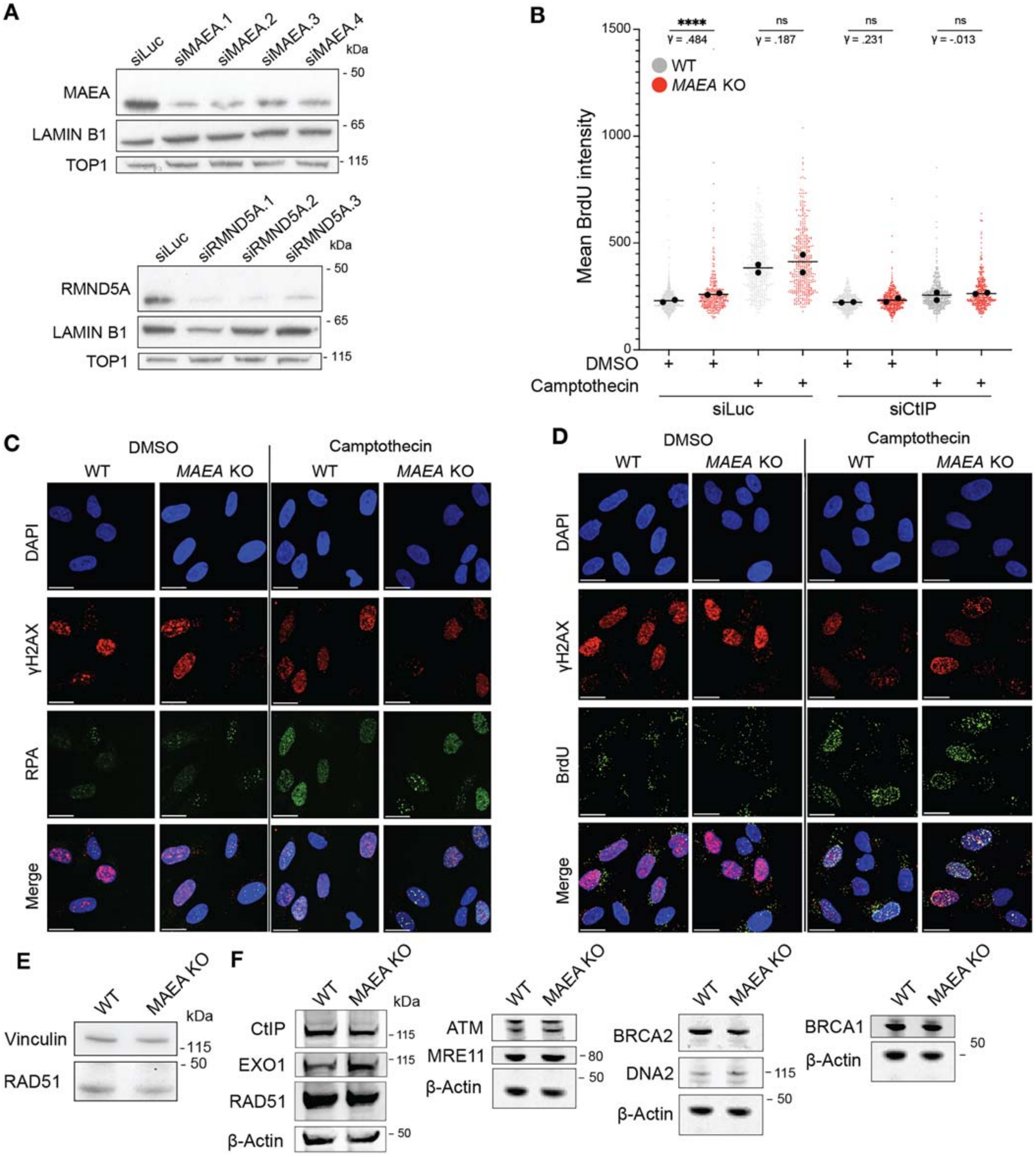

Figure EV3. MAEA loss compromises RAD51 foci formation.

(**A**) Immunoblot against the indicated proteins with the indicated siRNAs; $n = 3$ independent experiments. (**B**) Quantification of BrdU immunofluorescence, after camptothecin treatment in the presence or absence of siRNA-mediated CtIP depletion. (**C**, **D**) Representative images from Fig. 3C,E expanded to include γH2AX staining, indicative of the S phase cells. (**E**) Immunoblot against RAD51 in WT and *MAEA* KO U2OS cells. (**F**) Immunoblot for indicated proteins in U2OS WT or *MAEA* KO cells. (E, **F**) are each $n = 3$ independent experiments. *P* values were generated by performing a two-tailed Kruskal–Wallis test. γ is a measure of effect size. The data were the combined results of two independent experiments. Bars denote mean ± 95% CI. *$P \le 0.05$, **$P \le 0.01$, ***$P \le 0.001$, ****$P \le 0.0001$; exact *P* values can be found in Appendix Table S1. Scale bars = 20 µm. NT non-treated, KO knockout. Source data are available online for this figure.

 © The Author(s)

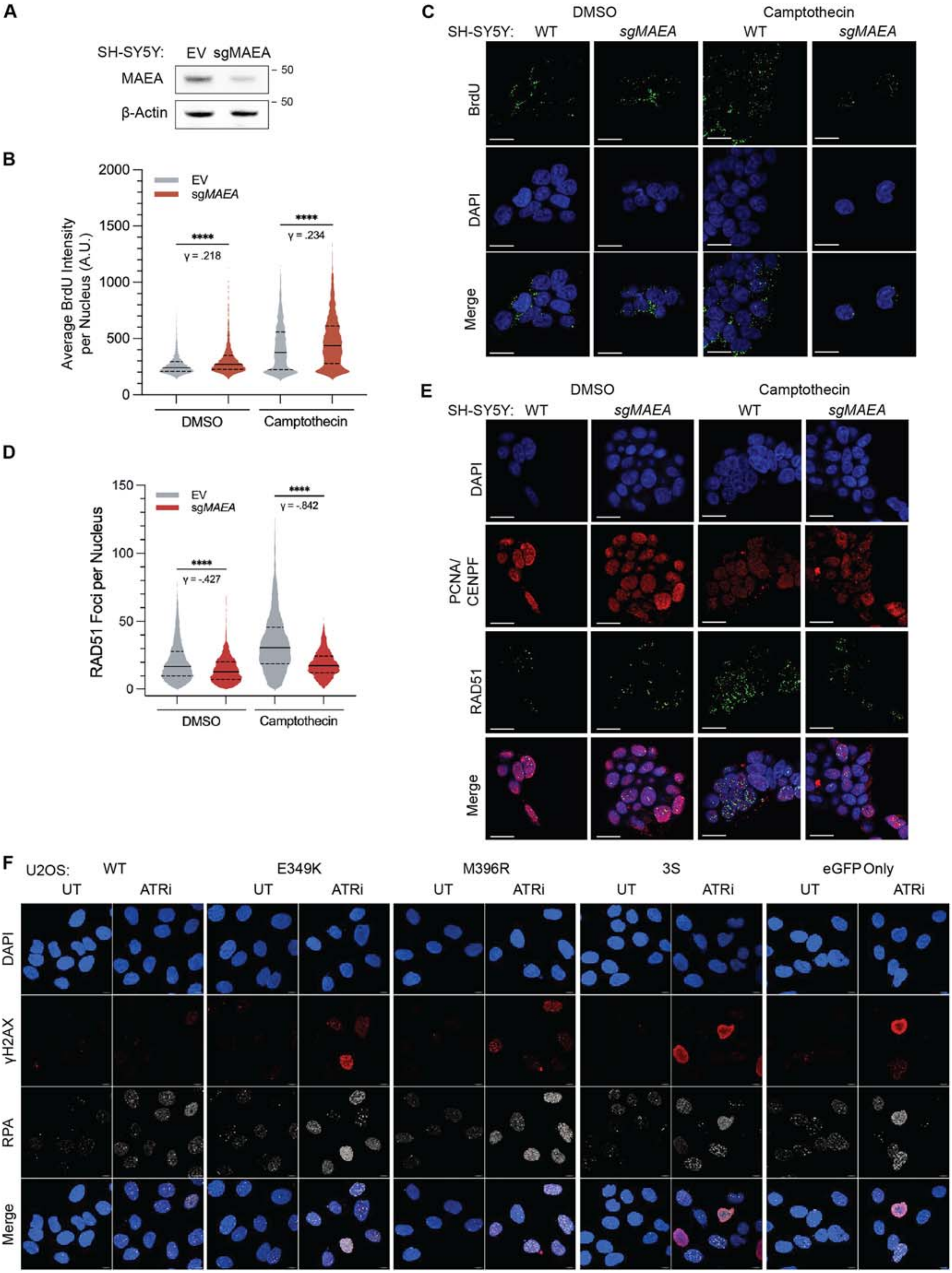
A
SH-SY5Y: EV sgMAEA
MAEA
β-Actin
50
50
B
Average BrdU Intensity per Nucleus (A.U.)
EV
sgMAEA
γ = .218
γ = .234
DMSO
Camptothecin
C
DMSO
Camptothecin
SH-SY5Y: WT sgMAEA WT sgMAEA
BrdU
DAPI
Merge
D
RAD51 Foci per Nucleus
γ = -.427
γ = -.842
E
PCNA/ CENPF
RAD51
F
U2OS: WT E349K M396R 3S eGFP Only
UT ATRi
γH2AX
RPA

Figure EV4. RAD51 loading defects in MAEA-deficient SH-SY5Y cells.

(**A**) Immunoblot for MAEA depletion in polyclonal SH-SY5Y cells following CRISPR-Cas9 editing; $n = 3$ independent experiments. (**B**) Quantification and (**C**) representative images of BrdU in SH-SY5Y cells treated with DMSO (1 h) or camptothecin (1 μM, 1 h). (**D**) Quantification and (**E**) representative images of RAD51 foci in S-phase SH-SY5Y cells treated with DMSO or camptothecin. Bars in (**B**, **D**) represent median and interquartile range. *P* values were generated by performing a two-tailed Kruskal–Wallis test. γ is a measure of effect size. The data represent three independent experiments. $^*P \leq 0.05$, $^{**}P \leq 0.01$, $^{***}P \leq 0.001$, $^{****}P \leq 0.0001$; exact *P* values can be found in Appendix Table S1. Scale bars = 10 μm. For (**B**, **C**), outliers were removed using ROUT analysis (Q = 1%). (**F**) Representative images of Fig. 5F. Source data are available online for this figure.

 © The Author(s)
